# Structured peer-led diabetes self-management and support in a low-income country: The ST2EP randomised controlled trial in Mali

**DOI:** 10.1371/journal.pone.0191262

**Published:** 2018-01-22

**Authors:** Xavier Debussche, Stéphane Besançon, Maryvette Balcou-Debussche, Cyril Ferdynus, Hélène Delisle, Laetitia Huiart, Assa T. Sidibe

**Affiliations:** 1 Department of Endocrinology Diabetology Nutrition, Felix Guyon University Hospital, Saint-Denis, La Réunion; 2 Centre d’Investigations Cliniques 1410 INSERM, Reunion University Hospital, Saint-Pierre, La Réunion; 3 Non-Governmental Organisation Santé Diabète, Bamako, Mali; 4 EA7389 Institut Coopératif Austral pour la Recherche en Education, University of Reunion, Saint-Denis, La Réunion; 5 UNIversités en Réseau pour l’éducation à la Santé, Clermont-Ferrand, France; 6 Unité de Soutien Méthodologique, CHU de la Réunion, Saint-Denis, La Réunion; 7 Department of Nutrition, Medicine Faculty of Montreal University, Montreal, Quebec, Canada; 8 Unité Mixte de Recherche 912 INSERM-IRD, "Sciences Économiques et Sociales de la Santé et Traitement de l'Information Médicale", Marseille, France; 9 Internal Medicine, University Hospital, Bamako, Mali; Florida International University Herbert Wertheim College of Medicine, UNITED STATES

## Abstract

**Objectives:**

Our objective was to evaluate the effectiveness of peer-led self-management education in improving glycaemic control in patients with type 2 diabetes in a low-income country (Mali).

**Methods:**

We conducted an open-label randomised controlled trial. A total of 151 adults (76% women, mean age 52.5) with type 2 diabetes (HbA1c≥8%), treated in the diabetes consultation units of two secondary health centres in Bamako, were allocated to peer-led structured patient education (n = 76) or conventional care alone (n = 75). The intervention group received 1 year of culturally tailored structured patient education (3 courses of 4 sessions) delivered in the community by five trained peer educators. Both groups underwent conventional diabetes monitoring and follow-up. Primary outcome was the mean absolute change in HbA1c from baseline to 12 months.

**Results:**

177 education sessions were delivered to the intervention group. Patient attrition was 8%. From baseline to 12 months, the decrease in HbA1c levels was 1.05% (SD = 2.0; CI95%: 1.54;-0.56) in the intervention group compared with 0.15% (SD = 1.7; CI95%: -0.56; 0.26) in the control group, p = 0.006. Mean BMI change was -1.65 kg/m2 (SD = 2.5; CI95%: -2.25; -1.06) in the intervention group and +0.05 kg/m2 (SD = 3.2; CI95%: -0.71; 0.81) in the control group, p = 0.0005. Mean waist circumference decreased by 3.34 cm (SD = 9.3; CI95%: -5.56;-1.13) in the intervention group and increased by 2.65 cm (SD = 10.3; CI95%: 0.20; 5.09) in the control group, p = 0.0003.

**Conclusions:**

Peer-led structured patient education delivered over 1 year to patients with poorly controlled type 2 diabetes in Mali yielded substantial improvements in glycaemic control and anthropometric parameters. This is of importance for the scaling up of efficient interventions in low-resource settings in the future.

**Trial registration:**

ClinicalTrials.gov NCT01485913

## Introduction

Type 2 Diabetes (T2D) is a worldwide challenge. According to the International Diabetes Federation, 415 million people had diabetes in 2015 [1]. Moreover, it is expected that by 2030, the burden of diabetes on mortality will be higher than that of malaria and HIV-AIDS combined, and that treatment will cost more than 465 billion dollars per year [2]. In Africa, the impact of diabetes on populations and the health system is huge, yet largely neglected in health research [3]. Africa’s chronic disease burden is largely attributed to increased sedentary lifestyle, modified diets with higher levels of added sugar, salt, total and saturated fat, as well as structural factors such as industrialisation, urbanisation and increasing food globalisation [4].

This is compounded by weak health systems that are unable to cope with the double impact of infectious and chronic diseases [5]. In Mali, life expectancy at birth is 58 years, and 79% of diabetes-related deaths occur in people under 60 [1]. In 2004, the country had among the lowest levels of health human resources in the world, limited access to health services, and extremely limited access to diabetes care services in particular [6]. From 2005 to 2010, the NGO *Santé Diab0078te* together with the Malian Ministry of Health developed an important programme to strengthen diabetes management services offered by the healthcare system. This led to the opening of 23 diabetes consultation units in health structures located in different regions of the country [7].

In order to improve the efficacy of this program, self-management education (SME) was introduced in 2009 to help patients cope with uncontrolled and often complicated diabetes. SME is a type of intervention that enables patients to acquire or maintain healthcare practices which help them manage life with a chronic disease as effectively as possible [8]. It has been recommended that SME interventions be based on theoretical approaches that take into account the complexity of the chronic disease [9–11] and that meet the specific needs of individuals in their social and cultural contexts [12–15]. In low- and middle-income countries (LMICs), SME led by community health workers and peer educators (PEs) has been reported to make major contributions in the areas of health promotion, undernutrition, maternal and child health, and epidemic infectious diseases [16]. However, in the case of non-communicable diseases (NCDs) such as cardiovascular complications and diabetes, the few studies performed on SME in low- and middle-income countries have revealed poor outcomes, contrary to what has been observed in high-income countries [17–19].

The Structured Type 2 Diabetes Self-Management Education by Peers (ST2EP) study in Bamako, the capital of Mali, was designed to supplement these findings. Our aim was to evaluate the 1-year efficacy of the SME intervention delivered in the community to poorly controlled T2D patients by *Santé Diabète*. This replicable, peer-led structured patient education intervention included quality-assessed course structure and materials adapted to learners’ cognitive, cultural and social characteristics [12]. It focused on four key objectives of secondary prevention in individuals with T2D: proper eating habits, regular physical activity, adherence to medical treatment, and regular monitoring checks [20]. The ST2EP study measured the efficacy of the intervention through comparing differences in blood glucose control (HbA1c levels) between patients who received SME and patients who were given standard care alone.

## Methods

### Study design

The ST2EP study is an open-label, randomised controlled trial designed to test the efficacy of a 1-year SME intervention in improving blood glucose control in patients with type 2 diabetes. Participants provided written informed consent in accordance with the WHO guidelines for good clinical practice. They were then randomised to two groups: The intervention group received peer-led structured patient education every three months for one year, and the control group was given conventional care alone. Participants in both groups continued to undergo conventional diabetes monitoring and regular follow-ups, which included individual counselling sessions, measurement of blood glucose, weight and blood pressure, data collection, clinical examination, and prescription or renewal of treatment. Clinical investigations were conducted according to the principles of the declaration of Helsinki. The study protocol was approved on June, 14^th^, 2011 by the institutional ethical committee of the *Faculté de Médecine de Pharmacie et d’Odonto-Stomatologie de Bamako*, # 55/2011/FMPOS. The authors confirm that all ongoing and related trials for this intervention are registered.

### Participants

Participants were recruited from the lists of active patients held by consultation units located in two secondary health centres of Bamako. Both sites selected offered structured care as well as medical treatment and included an association of diabetic patients.

Patients included in the study were aged between 30 and 80 years, underwent regular follow-ups and monitoring in Bamako consultation units for poorly controlled T2D (HbA1c ≥ 8%), accepted to attend all peer-led educational sessions, and agreed to have their clinical and biological measurements taken until completion of the protocol. Patients with type 1 diabetes were excluded from the study, as were those who suffered from severe complications in the 3 months preceding enrolment (infections, severe renal failure, coronary events, foot lesions) or from concomitant illnesses that threatened their functional or vital prognosis.

### Intervention

The ST2EP intervention included 3 courses delivered in the community by trained peer educators over 1 year. Each course was composed of 4 different thematic sessions (4–10 participants) offered over a period of 3 months (months 1–3, 7–9, and 10–12). The initial protocol stated 1.5 hour-long sessions, however, the actual duration of sessions during the trial with peer educators was 1.5–2 hours. The themes addressed were cardiovascular risk management, food intake, exercise, and blood glucose and insulin management. The content, approach and programme of each group session were detailed in specific booklets for learners (including learners with literacy difficulties) and culturally adapted for Mali (food habits, language specificities, occupational and environment issues). The cultural adaptation of the educational materials and booklets was performed with the help of the association of diabetic patients involved in the project since 2009. Peer educators were also given specific booklets that comprised the session programme in French (*Education Prévention des Maladies Chroniques* or EPMC booklets [12]), allowing for the replication of the educational intervention.

The peer-led structured patient education intervention drew on the ‘Learning Nests’ (*Nids d’apprentissage*) approach, which has been described elsewhere [12, 21, 22]. Briefly, this empowerment-based [23] approach, derived from socio-constructivist theory [24], takes into account the context of the illness, prevailing health practices [25], and the chronic dimension of the disease [26]. It promotes patients’ understanding of key concepts in their interactions with their social environment. Educational sessions are structured around 5 components: patients’ analysis of their own knowledge and practices, the recognition of individual contexts and problem-solving, individual heterogeneity as an asset for self-assessment and action, culturally-tailored educational materials, and the long-term dimension of disease management. The ‘Learning Nests’ approach incorporates and emphasises behavioural strategies as opposed to traditional didactic teaching. These strategies include the implementation of curricula and the distribution of hand-outs designed to meet the specific needs of target populations.

### Peer educators

The peer educators (PEs) were recruited from the local association of diabetic patients. This association offers counselling, education and support services to people with diabetes, and hence provides a key link between patients, educators and primary care services.

The following criteria were used to select 10 PEs from the list of association members: having diabetes, living in the locality, undergoing regular checks with a referent physician, volunteering to deliver educational sessions, and being fluent in both French and the local Bambara language. The recruited PEs attended an initial 4-day training program on how to facilitate and structure the different sessions (which focused on cardiovascular risk management, food intake, exercise, and blood glucose and insulin management). Training included for each thematic group sessions a thorough review of the process, content, educator role, learner’s actions, learning indicators, specificity of goal setting and problem solving taking into account individual, social and cultural context. At the end of this training, a grid test was conducted to assess whether the PEs had understood the methodology and were qualified to facilitate the sessions. All 10 PEs were selected for another round of training, during which a 2^nd^ assessment grid was used to evaluate the facilitation skills of the PEs, under the supervision of the research team. A final evaluation was performed with the same assessment grid to select the 5 best PEs, who were then actively involved in the project.

### Outcome measures

Outcome measures were taken at baseline and at 3, 6, and 12 months. Biomedical data were collected during practice visits in secondary health centres. The main outcome was the difference in HbA1c levels between baseline and 12 months for the two groups. The secondary outcomes were as follows: evolution of HbA1c levels within 12 months after baseline, differences in anthropometric indicators (weight and body mass index [BMI], waist circumference [WC]), systolic and diastolic blood pressure, anti-diabetic and anti-hypertensive treatment, knowledge score, and dietary practices between baseline and 12 months.

Data were collected according to standard operating procedures. HbA1c levels were measured on capillary whole blood using CloverA1c (Infopia Co., Ltd., South Korea). Reagents were carefully checked throughout the study so as to avoid deterioration in high temperatures.

Clinical data were collected by the 2 clinicians in charge for the medical management and follow-up of patients. Waist circumference (WC) was measured in the upright position, on a bare abdomen and at the top of the iliac crest using a simple tape measure. Diabetes knowledge was assessed via a 77-open items questionnaire (diabetes symptoms, anti-diabetic treatment, complications, hypo-hyperglycemia, exercise, diet, foot care) with a maximum total score of 11 S1 File. This questionnaire was developed by *Santé Diabète* (SB) together with the Bamako University Hospital (AST) and used in several field evaluations. Dietary practices at baseline and at 12 months were compared between the intervention group and the control group, as well as within each group using a non-quantitative 24-hour dietary recall and a food frequency questionnaire. A food diversity score was computed based on the presence/absence of 10 food groups in the 24-hour recall, with a maximum score of 10. The food-frequency questionnaire included the consumption frequency of 34 common foods and the number of meals and snacks eaten during the previous week [27]

### Patient involvement

Patients were not involved in setting the research question or the outcome measures; nor did they participate in the design or implementation of the study. Educational materials and booklets were culturally adapted with the help of the association of diabetic patients involved in the project since 2009. The association directly contributed to the recruitment of peer educators.

No patients were asked to advise on interpretation or writing up of results. We carefully assessed the effects and potential pitfalls of the intervention by collecting information on the time use of patients and peer educators. The results of the research were later provided to study participants, PEs, and the local association of patients, via meetings and information resources made available in consultation units and in *Santé Diabète* offices in Bamako.

### Eligibility and randomisation

Eligible T2D patients were identified among patients treated for diabetes. A random allocation sequence was generated to randomise participants to the intervention group or to the control group with a ratio of 1 to 1. The methodologist who performed the randomisation was not directly involved in the recruitment of patients. Recruiting physicians were blinded to the allocation sequence.

### Statistical analysis (sample size)

Our aim was to include 150 patients. The sample size for the study was calculated based on a power of 80% to detect a minimum absolute difference of 1.0% in HbA1c, assuming a basal value of 8.5% with a 2.0% standard deviation and a two-sided α of 0.05. According to our calculations, 60 subjects had to be included in each trial arm. Allowing for a dropout rate of 20%, we decided to recruit 75 patients per arm (150 patients in total) for the trial.

We described quantitative variables using means and standard deviations or medians and interquartile ranges, and categorical variables using proportions and 95% confidence intervals. We compared categorical variables using the Chi-Square test, and quantitative variables using the Student’s t test. Comparisons of paired categorical variables were performed using McNemar's chi-square test for paired samples.

The primary outcome was change in HbA1c levels between baseline and 12 months, and it was compared between the two trial arms using the Student’s t-test. Changes in BMI, WC, systolic blood pressure, diastolic blood pressure, and diabetes knowledge score between baseline and 12 months were also compared between the two arms using the Student’s t-test. For each parameter, Cohen’s d and its 95% Confidence Interval (95%CI) were calculated as a measure of effect size. The evolution of the different parameters at baseline, 3, 6 and 12 months was analysed using linear mixed models with a first-order autoregressive variance-covariance matrix. The mixed models included fixed effects for time (baseline, 3, 6 and 12 months), trial arms, interactions between time and arm, and random effects for patients.

Patients were analysed according to the trial arm to which they were randomised (intent-to-treat analyses). All tests were two-sided. Statistical analyses were performed using SAS 9.4 (SAS Institute, Cary Inc.).

## Results

**Fig 1:**
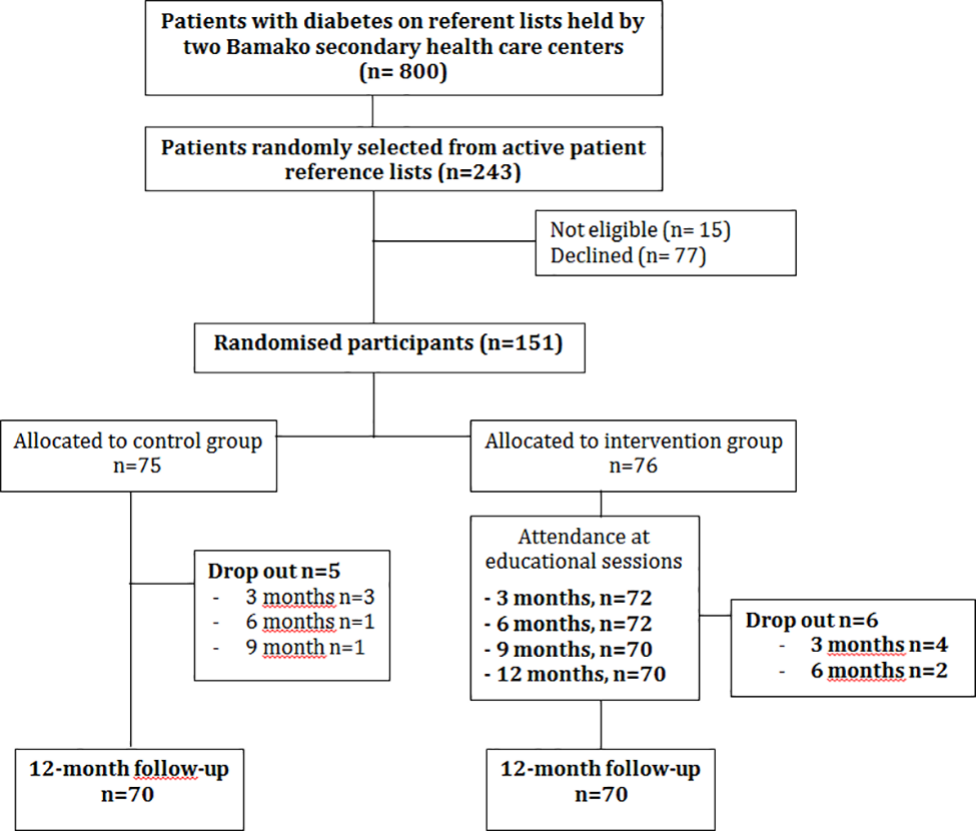
Flow chart of participants. ST2EP trial.

From July, 21st to September, 30^th^, 2011, we included 151 patients in the study: 76 in the intervention group and 75 in the control group (Fig 1). Study participants were mostly female (n = 115, 76.2%) and the majority were overweight (BMI> = 25: n = 110, 72.9%). A third of the sample presented high blood pressure (n = 51, 33.8%). The mean HbA1c level was 10.6% (standard deviation (sd = 1.8) and 10.8% (sd = 1.9) in the intervention arm and the control arm, respectively. At baseline, patients’ characteristics in the 2 groups were similar (Table 1). Educational courses took place between October and December 2011, April and July 2012, August-December 2012. The follow-up of participants from both groups took place between 1^st^ October 2011 and 20^th^ February 2013. The reasons for this slight delay in scheduled courses and follow-ups were that due to political events in Mali during 2012 we had to post pone the second and third education session courses in the intervention arm, and the corresponding follow-ups (February 2012, July 2012, January 2013). The patterns of withdrawal are documented in Fig 1. Overall, the 5 PEs facilitated 177 educational sessions with a mean of 6.4 patients per group session in the intervention group. Among intervention patients, 70 attended the 3 courses.

**Table 1:** Clinical and biological characteristics at baseline of participants allocated to peer-led structured patient education (intervention) or to conventional care alone (control).

	Total (n = 151)	Intervention (n = 76)	Control (n = 75)
Gender (female, n, %)	115 (76.2)	57 (75.0)	58 (77.3)
Age, years	52.5 (9.8)	53.9 (9.8)	51.1 (9.6)
BW, kg	78.2 (15.6)	77.7 (16.4)	78.7 (14.7)
BMI, kg/m^2^	28.6 (5.4)	28.3 (5.4)	28.8 (5.5)
Waist circumference, cm	93.9 (11.9)	93.7 (12.1)	94.1 (11.6)
BMI 25.0–29.9	48 (31.8%)	21 (27.6%)	27 (36.0%)
BMI≥30.0	62 (41.1%)	34 (44.7%)	28 (37.3%)
BP Diastolic, mm Hg	81.6 (10.6)	82.9 (10.5)	80.3 (10.6)
BP Systolic, mm Hg	129.9 (23.9)	132.8 (26.9)	127.1 (20.1)
HbA1c, %	10.7 (1.8)	10.6 (1.8)	10.8 (1.9)
No treatment / Diet only	13 (8.6%)	7 (9.2%)	6 (8.0%)
Oral anti-diabetic only	92 (60.9%)	45 (59.2%)	47 (62.7%)
Insulin only	32 (21.2%)	15 (19.4%)	17 (22.7%)
Insulin and oral anti-diabetic	14 (9.3%)	9 (11.8%)	5 (6.7%)
Knowledge score	5.2 (1.2)	5.2 (1.2)	5.2 (1.3)

Values are n (%) or mean (SD). BMI = Body Mass Index. BW = Body weight. BP = Blood pressure

As shown in Table 2, the reduction in HbA1c levels between baseline and 12 months was higher (p = 0.006) in the intervention group (-1.05 [sd = 2.0]) than in the control group (-0.15 [sd = 1.7]). The effect size was 0.48 (CI95%: 0.14–0.81). BMI and WC had also improved at 12 months in the intervention group. Similarly, in this latter group, the proportion of patients receiving insulin decreased slightly from 28.9% (n = 22) to 24.3% (n = 17) during follow-up in the intervention group (p = 0.0003) (Table 3).

**Table 2:** Changes in biomedical outcomes from baseline to 12 months. The ST2EP trial in Mali. Data are mean (CI 95%).

	Intervention group (n = 70)	Control group (n = 70)	p-value
Body Mass Index, kg/m^2^	-1.65 (-2.25; -1.06)	0.05 (-0.71; 0.81)	0.0005
Waist circumference, cm	-3.34 (-5.56;-1.13)	2.65 (0.20;5.09)	0.0003
Systolic BP, mm Hg	-6.46 (-11.63;-1.28)	3.57 (-0.17;7.31)	0.003
Diastolic BP, mm Hg	0.40 (-2.27;3.07)	2.00 (-0.77;4.77)	0.36
HbA1c, %	-1.05 (-1.54; -0.56)	-0.15 (-0.56; 0.26)	0.006
Knowledge score	1.06 (0.70;1.42)	0.61 (0.23;0.99)	0.17

**Table 3:** Anti-diabetic and anti-hypertensive treatment in intervention group and in control group at baseline and at 12-month follow-up (FU).

	Total	Intervention group	Control Group	p-value
**At baseline**	**n = 151**	**n = 76**	**n = 75**	
Insulin	43 (28.5%)	22 (28.9%)	21 (28.0%)	0.90
OAD only*	108 (71.5%)	55 (72.4%)	53 (70.7%)	0.82
Combination of 2 OADs**	108 (71.5%)	55 (72.4%)	53 (70.7%)	0.82
Calcium channel blockers	7 (4.6%)	5 (6.6%)	2 (2.7%)	0.44
Beta Blockers	4 (2.6%)	2 (2.6%)	2 (2.7%)	0.99
Angiotensin Receptor Blockers	23 (15.2%)	15 (19.7%)	8 (10.7%)	0.12
Central	1 (0.7%)	1 (1.3%)	0 (0.0%)	1.00
Diuretics	1 (0.7%)	1 (1.3%)	0 (0.0%)	1.00
**At the end of FU**	**n = 140**	**n = 70**	**n = 70**	
Insulin	36 (25.7%)	17 (24.3%)	19 (27.1%)	0.70
OAD* only	102 (72.9%)	51 (72.9%)	51 (72.9%)	1.00
Combination** of 2 OADs	100 (71.4%)	50 (71.4%)	50 (71.4%)	1.00
Calcium channel blockers	11 (7.9%)	6 (8.6%)	5 (7.1%)	0.75
Beta Blockers	6 (4.3%)	3 (4.3%)	3 (4.3%)	1.00
Angiotensin Receptor Blockers	36 (25.7%)	22 (31.4%)	14 (20.0%)	0.12
Central	0 (0.0%)	0 (0.0%)	0 (0.0%)	NC
Diuretics	3 (2.1%)	1 (1.4%)	2 (2.9%)	1.00

Data are n (%)

*OAD: Oral Antidiabetic therapy comprising metformin and/or sulfonylurea and/or alpha-glucosidase inhibitors.

**Combination of 2 OAD: Combination of metformin and sulfonylurea

**Fig 2:**
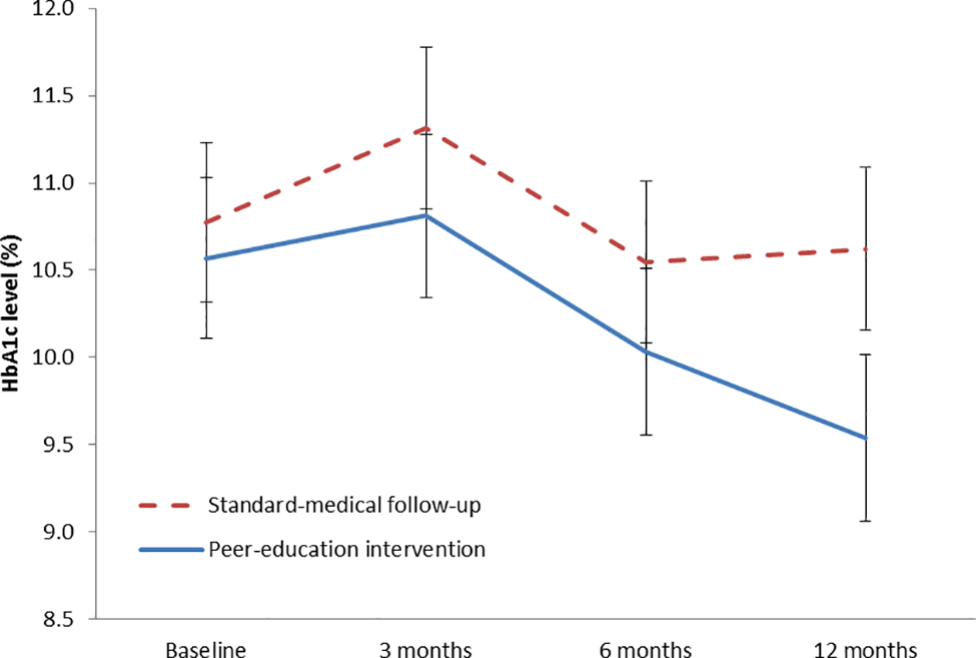
Evolution of HbA1c levels between baseline and 12 months–levels in % and 95% confidence interval.

**Fig 3:**
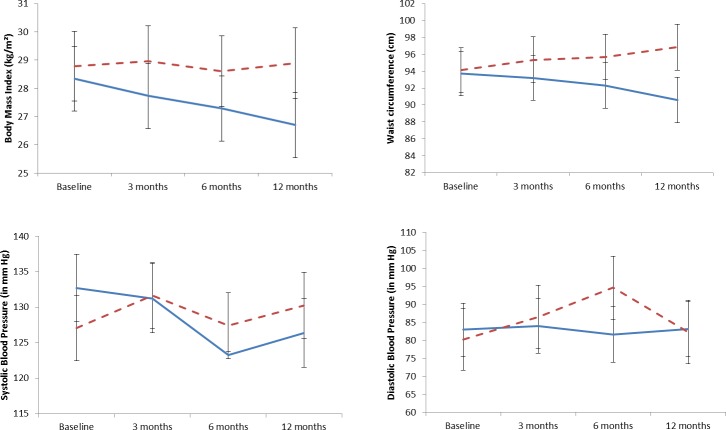
Evolution of body mass index, waist circumference and blood pressure between baseline and 12 months– 95% confidence interval.

The evolution of HbA1c levels between baseline and 12 months is presented in Fig 2. The decrease in HbA1c levels was more important in the intervention group, especially after month 6. Similarly, the evolution of BMI and WC was more favourable in the intervention group than in the control group (p = 0.001 for BMI and WC; Fig 3). Patients’ knowledge scores improved slightly, mostly due to their increased knowledge of symptoms, treatment and hypoglycaemia management (Table 4). No positive change in the diet diversity score as a crude index of diet quality was recorded in intervention patients. The number of meals remained the same. However, qualitative changes in the diet were noted from dietary recalls at baseline and at the end of follow-up in the various meals and snacks eaten during the day: meat and fish, as well as peanut and onion sauce, consumption was more abundant at baseline, the number of patients who mentioned having a morning snack decreased by more than half at follow-up,

**Table 4:** Knowledge score changes between baseline (T0) and end of follow-up (12 months) in intervention group (n = 70) and in control group (n = 70).

Knowledge score domains (EFU–T0)	Total	Intervention group	Control Group	p-value
Symptoms	0.04 (0.40)	0.13 (0.39)*	-0.06 (0.39)	0.003
Treatments	0.00 (0.47)	0.03 (0.46)	-0.04 (0.47)	0.39
Treatments adherence	0.10 (0.24)*	0.12 (0.22)*	0.07 (0.26)*	0.17
Sign of gravity	0.00 (0.24)	0.00 (0.24)	0.01 (0.25)	0.73
Symptoms of hypoglycemia	0.12 (0.31)*	0.12 (0.31)*	0.12 (0.31)*	0.94
Hypoglycemia management	0.04 (0.43)	0.09 (0.43)	0.00 (0.44)	0.26
Diabetic foot	0.02 (0.22)	0.02 (0.23)	0.02 (0.22)	0.92
Physical activity	0.39 (0.54)*	0.40 (0.53)*	0.37 (0.55)*	0.78
Diet	0.11 (0.21)*	0.13 (0.22)*	0.10 (0.20)*	0.40

Changes are scores at end of follow-up minus scores at baseline. Data are mean (SD).

* p<0.05 for increase between baseline and end of FU.

## Discussion

The ST2EP study is the first randomised controlled trial (RCT) to report positive results on peer-led structured patient education in Africa. Our SME intervention in Mali led to improvements in HbA1C levels and to a decrease in body weight and waist circumference in the intervention arm compared to the control arm. However, although our study brought evidence for the usefulness of the intervention, the mean values of HBA1c (%) at 12 months post intervention was still high.

These positive results may be due to the high-quality training and coaching provided to PEs by *Santé Diabète* staff, but also to the fact that the educators’ active involvement guaranteed high levels of attendance at the educational sessions. The peer-led sessions were delivered as a complement to structured medical follow-ups in urban community health centres, which suggests that the potential for reaching those suffering from diabetes complications was high. Four key elements of success have to be highlighted: the rigorous and careful process of recruiting, selecting and training peer educators; the adaptation of the Learning nests approach to the African context; the strong involvement of diabetic patients in education sessions and the strong involvement of the local diabetic patient association; and the development of patient education sessions despite the low number of human resources. The involvement of peers has made possible to circumvent the weakness of human resources in the Malian health system and thus to really implement and set up education for patients.

In the present study, the mechanisms that were triggered by the peer groups leading to the decrease in HbA1C and better BMI are not clear. However, increased knowledge of symptoms and some behavioral changes were noted in the qualitative composition of the diet. These changes are in line with the activities of learners during education sessions (making practical choices about snacks, fatty meals, meat quantity, and confronting to contextual familial and social issues). Unfortunately, two other key potential factors of changes have not been assessed in this study: the level of physical activity, and the adherence to antidiabetic therapy.

A review of the literature reveals that peer educators and community health workers have been increasingly involved in health promotion and therapeutic education. This has led to improvements in biomedical and patient related outcomes, especially among underserved populations and ethnic minorities in high- or middle-income countries [16]. The meta-analysis of peer support intervention, by Zhang *et al*. [28], examined 20 RCTs, among which 17 were performed in the US or the UK and 3 in LMICs (1 in Argentina, 1 in China and 1 in Iran). The pooled effect on HbA1c was -0.16%. “Peer leader interventions” and “curriculum combined reinforcement intervention” exhibited better outcomes with pooled effects of -0.49% and -0.24%, respectively. Studies of type 2 diabetes SME programmes implemented in developing countries were conducted mainly in low mortality countries and seldom involved peer educators [29]. Only 17% of these programmes provided data on the training of providers, and 39% were designed to be accessible for people with low literacy. The few studies conducted in high-mortality countries were quasi-experimental for the most part, and they were rarely tailored to specific cultural contexts [29]. In low-income countries, and especially in Africa, peer-led educational interventions have led to improvements in clinical outcomes and dietary behaviour, as reported in the initial assessment of the Peers for Progress projects in Cameroon, Uganda and South Africa [18]. However, these studies may contain biases as they were performed on a before-after basis. By contrast, Mash *et al*. [19] conducted a cluster-randomised trial on a group education intervention led by health promoters in South Africa. This study based on motivational interviewing techniques reported negative results, including poor attendance at sessions (<60%) and low adherence of health promoters. While blood pressure slightly improved, there was no improvement in glycaemic control or weight.

Several factors may help explain the positive results obtained in the present ST2EP study: First, the intervention was culturally tailored with the help of patients, health professionals and local PEs. Secondly, the “dosage” of planned and effectively conducted group education was high, as it included 3 quarterly courses of 4 sessions each. The importance of “dosage” and maintenance duration for SME effectiveness has been highlighted in other contexts [30]. Thirdly, the Learning nests approach incorporates and emphasizes behavioral strategies, unlike a more traditional didactic approach, including curricula and handouts to meet the specific needs of the target population, and the needs of peer educators. It also involves psychosocial dimensions, taking into account the context in which the chronic illness is dealt with on a daily basis. The patient has the possibility to modify his everyday practices (nutrition, physical activity, taking medication) while considering his/her existence in relation with the others, with his environment, his resources and his culture [12,22]; Peer educators have been trained to conduct interactive and constructive sessions in which learners work on knowledge elements, and as well confront the practical applications in the context of everyday life. The PE does not explain the mechanisms that learners (patients) will be led to understand by other means. On the contrary: he prepares the material layout of the situation; he gives directions and instructions; he helps the patient to acquire the knowledge; he observes what patients do and listens to what they say; he educates the attention ability of the learner; he helps patients who have particular difficulties (fluency in the French language, written language, embarrassment in social relations); and he stimulates and supports the activity of participants. The reality of these activities of PEs was confirmed in a qualitative assessment of sessions [31].

The present study has some limitations. First, cluster randomisation was not feasible because of the insufficient number of health centres we could include in the study. Randomisation consequently had to be performed at the participant level, which may have led to contamination between groups. Yet such contamination would not alter our conclusions, as it would cause positive results in the intervention group to be underestimated. Second, the study could not be blinded, which means that biases may have occurred in the comparative evaluation of diabetes management and follow-up between the two groups. It should nonetheless be noted that there was no difference in the provision of anti diabetic and blood pressure treatment or in patients’ attendance at follow-up between the two groups. Third, we conducted our study in the capital of Mali, and hence in an urban setting that markedly differs from the country’s rural and remote areas and from other regions of Africa, suggesting that one should be cautious in extrapolating our results. Yet, while the course structure and materials were specifically adapted to the African and Malian contexts, the pragmatic and culturally sensitive nature of this intervention allows for its replication in other areas. Indeed, it has already been tested in Reunion Island, Burundi, and Mayotte with positive results in terms of feasibility and patient involvement [12, 21, 22, 31].

Overall, the results of the ST2EP study are encouraging, even though the long-term effects of this study have yet to be evaluated [28, 29]. The ‘Learning Nests’ approach was successfully adapted to the African context. The procedure put in place for the recruitment, selection and training of patients and peer educators was feasible and realistic, and included a preliminary qualitative study based on the observation of educational sessions with trained educators [31]. Despite the scarcity of human resources in the Malian health system, diabetic patients were strongly involved in the study and the educational sessions were effectively delivered. The above had a strong impact on glycaemic and anthropometric outcomes, indicating that our intervention should be tested in other low- and middle-income countries. Successful scaling up will nevertheless require consideration of organisational issues at the regional and national levels, assessment of local needs and resources, as well as community engagement and the involvement of staff and stakeholders [32].

## Supporting information

S1 File Knowledge questionnaire. French original questionnaire (questionnaire de connaissance) and English translation.
(DOC)

S2 File Study protocol. French protocol Version 1 (v1) and Version 2 (v2); English translation of the protocol (v2); Acceptation letter from the Malian Ethical Comitee.
(ZIP)

S3 File CONSORT checklist. (DOC)

S4 File TIDieR checklist. (DOC)
