# Supplementary material for: Structured peer-led diabetes self-management and support in a low-income country: The ST2EP randomised controlled trial in Mali
Source: PLoS One. 2018 Jan 22;13(1):e0191262. doi: 10.1371/journal.pone.0191262 (PMC5777645; doi:10.1371/journal.pone.0191262)
Supplement: S1 File — French original questionnaire (questionnaire de connaissance) and English translation. (DOC) [file pone.0191262.s001.doc]

**Questionnaire de connaissance**

**Etude ST2EP Bamako**

| 1 | **symptomes du diabète** |  |
| --- | --- | --- |
| 1  q1 Quels sont les symptômes du diabète ?   - Polyurie (miction excessive) - Polydipsie (soif excessive) - Polyphagie (faim excessive) - Fatigue (asthénie) | | 3 symptôme = 100%  2 symptômes = 50%  1 symptômes =0% |
| 2  q2 Quels sont les moyens de traitement du diabète sucré ?   - Régime alimentaire - Insuline - ADO - Insuline + ADO | | 3 moyens = 100%  2 moyens = 50%  1 moyen = 0% |
| 3  Q3 Il existe des médicaments pour guérir le diabète faux  Q4 Il existe des médicaments pour stabiliser le diabète vrai  Q5 Certains diabétiques doivent obligatoirement faire   des injections. vrai  Q6 Connaissez-vous l’insuline ?  Q7 A quoi sert l’insuline ?   - Diminuer la glycémie   Q8 Un diabétique doit s’abstenir de manger  certains aliments. faux  Q9 Une alimentation équilibrée est un moyen de  traitement du diabète vrai | | Plus de 5 bonnes réponses = 100%  3 à 4 bonnes réponses = 50%  1 à 2 = 25% |
|  | **sous total 1** | **3** |
| 4 | **suivi des médicaments** |  |
| Q1 Quels sont les sites d’injection de l’insuline que vous  connaissiez ?   - Cuisses - Bras - abdomen   Q2 Comment doit on conserver nos insulines ?   - frais   Q3TOUS les médicaments antidiabétiques doivent être pris  avant les repas faux  Q4 Un diabétique peut changer son insuline en comprimés  s’il le désire faux  Q5 Certains diabétiques peuvent se passer de médicaments vrai  Quelles sont les facteurs de risque cardio-vasculaire ?   - **diabète** - **HTA** - **Excès de graisse** - **Obésité** | | 6 bonnes réponses = 100%  4 à 5 bonnes réponses = 75%  2 à 3 bonnes réponses = 50%  1 bonne réponse = 25%  0 bonne réponse = 0% |
|  | **sous toal 2** | **1** |
| 5 | **signes de gravités** |  |
| Q1 Quels sont les signes qui peuvent vous faire penser à  une aggravation de votre diabète ?   - Néphropathie - Neuropathie - rétinopathie   Q2 L’apparition d’une abondance des urines,  d’une perte de poids, d’une fatigue est un signe  d’aggravation du diabète vrai  Q3 Une fièvre chez un patient diabétique ne nécessité pas  de consultations immédiate chez un médecin faux | | 2 bonnes réponses avec 3 signes = 100%  2 bonnes réponses avec 2 signes = 75%  2 bonnes réponses avec 1 signe = 50%  2 réponses sans signe d’aggravation=25%  moins de 2 réponse sans signes d’aggravation=0% |
| 6  Q4 Chez les diabétiques, le taux de sucre dans le sang peut  descendre en dessous de la normale, ce qui n’est pas bon.  Quels sont les symptômes annonciateurs de cette chute  du taux de sucre sanguin ?   - tremblement - sueur - étourdissement - pâleur du visage - somnolence - manque de concentration - incohérence dans le discours | | 4 symptômes hypoglycémies = 100%  3 symptômes = 75%  2 symptômes = 50%  1 symptôme=25% |
| 7  Q5 Comment peut-on éviter la chute du taux de sucre dans le sang ?   - suivre le régime alimentaire - respecter les doses des médicaments prescrites   Q6 Comment doit-on corriger cette hypoglycémie  (chute du taux de sucre dans le sang) ?   - morceau de sucre ou sucrerie | | Prévention+traitement =100%  Prévention = 50%  Traitement = 25% |
|  | **sous total 3** | **3** |
|  |  |  |
| 8 | **le diabétique et ses pieds** |  |
| Q1 Le diabétique peut marcher pieds nus. faux  Q2 Le diabétique doit toutes les nuits vérifier l’intérieur de  la plante de ses pieds avant de dormir vrai  Q3 une plaie apparaît sur le pied du diabétique il doit faire  lui-même un pansement immédiatement faux  Q4 Les plaies ou douleur des pieds nécessitent une  consultation vrai  Q5 Le diabétique doit protéger ses pieds contres la  macération, les sources de chaleurs et les objets tranchants vrai | | 5 bonnes réponses= 100%  3 à 4 bonnes réponses= 50%  1 à 2 réponses = 25% |
|  | **sous total 4** | **1** |
|  | **hygiène de vie et diabète** |  |
| 9 | **activité physique** |  |
| Q1 diabétique doit consacrer un peu de temps à la marche  par jour vrai  Q2 Les personnes âgées doivent éviter de marcher pour  ne pas avoir des problèmes de cœur faux  Q3 Combien de temps par jour minimum devez vous faire  de l’activité physique chaque jour?   - 30mn | | 3 bonnes réponses = 100%  2 bonnes réponses = 50%  1 bonne réponse = 25%  0 bonne réponse = 0% |
| **Sous total 5** | | **1** |
| Q4 Pouvez-vous classer ces activités physiques :  Faible Modérée Importante  Q4-1 Faire le ménage   - Modéré   Q4-2 Conduire   - faible   Q4-3 Faire de la marche à pied   - importante   Q4-4 Travailler comme secrétaire   - faible   Q4-5 Courir   - important   Q4-6 Danser  important | | Plus de 5 bonnes réponses = 100%  4 à 5 bonnes réponses = 50%  2 à 3 bonnes réponses = 25%  Moins de 2 bonnes réponses = 0% |
|  | **sous total 6** | **1** |
| 10 | **Diététique** |  |
| Q1 Manger beaucoup est un signe de bonne santé faux  Q2 Les graisses sont aussi nocives que les sucres rapides  pour le corps vrai  Q3 Un diabétique peut manger les fruits vrai  Q4 Les diabétiques peuvent manger toutes les céréales vrai  Q5 Les diabétiques ne doivent manger que du fonio faux  Q6 Le diabétique doit manger de la salade et des légumes vrai  Q7 Citer 2 féculents  Q8 Citer 2 produits laitiers  Q9 Citer 2 fruits et 2 légumes  Q10 Citer 2 produits gras  Q11 Citer 2 produits sucrés  Q12 Pourquoi faut-il manger moins salé ?   - problème HTA - pour éviter les problèmes cardiovasculaires   Q13 Quels aliments doit on contrôler pour manger moins salé ?   - les conserves   Q14 Pourquoi faut-il manger moins gras ?   - pour éviter les problèmes cardiovasculaires   Q15 Quels aliments doit on contrôler pour manger moins gras ?  Q16 Pourquoi faut-il manger moins sucré ?   - Pour éviter les complications du diabète   Q17 Quels aliments doit on contrôler pour manger moins sucré ?  Q18Quels aliments doit contenir un repas équilibré ?   - Les féculents - Fruits - Les produits laitiers - Viandes et poissons - Eau   Q19 Un diabétique doit il grignoter entre les repas   - Non | | plus de 15 bonnes réponse = 100%  plus de 10 bonnes de réponse = 75 %  plus de 5 bonnes réponses = 50%  2 à 4 bonnes réponses = 25%  moins de 2 bonne réponse= 0 |
|  | **sous total 7** | **1** |

- 100%=1

- 75%=0,75

- 50%=0,50

- 25%=0,25

- 0%=0

|  | Totaux | **11** |
| --- | --- | --- |
| 1 | sous total 1 | 3 |
| 2 | sous total 2 | 1 |
| 3 | sous total 3 | 3 |
| 4 | sous total 4 | 1 |
| 5 | Sous total 5 | 1 |
| 6 | sous total 6 | 1 |
| 7 | sous total 7 | 1 |

>= 10 : très satisfaisant

5 à 10 : Satisfaisant

< 5 Insatisfaisant

**Knowledge questionnaire – English translation**

| 1 |  |  |
| --- | --- | --- |
| 1  q1 What are the symptoms of diabetes ? • Polyuria (excessive urination) • Polydipsia (excessive thirst) • Polyphagia (excessive hunger) • Fatigue (asthenia) | |  |
| 2  q2 What are the ways to treat diabetes mellitus? • Diet • Insulin • ADO • Insulin + ADO | |  |
| 3  Q3 There are medicines that cure diabetes (false) Q4 There are drugs to stabilize diabetes (true) Q5 Some diabetics imperatively need insulin injections. (true) Q6 Do you know insulin? Q7 What is insulin for? • Reduce blood sugar Q8 A diabetic person must refrain from eating  certain foods. (false) Q9 A balanced diet is a way to treat diabetes (true) | |  |
|  | **sub total 1** | **3** |
| 4 |  |  |
| Q1 What are the insulin injection sites that you you know? • Thighs • Arms • abdomen Q2 How should we keep our insulins? • fresh Q3 ALL antidiabetic drugs should be taken before fake meals (false) Q4 A diabetic person can change his insulin into tablets   if he wants it (false) Q5 Some diabetics can do without medications (true)  What are the cardiovascular risk factors? • diabetes • HTA • Excess fat • Obesity | |  |
|  | **subtotal 2** | **1** |
| 5 |  |  |
| Q1 What are the signs that can make you think diabetes is worsening? • nephropathy • neuropathy • retinopathy Q2 The appearance of urine abundance, weight loss, fatigue is a sign worsening of diabetes (true) Q3 A fever in a diabetic patient is not a reason for immediate consultation with a doctor (false) | |  |
| 6  Q4 In diabetics patients, the blood sugar level may go below normal, which is not good. What are the warning signs of this fall in blood sugar? • tremor • sweat • dizziness • pallor of the face • drowsiness • lack of concentration • inconsistency in speech | |  |
| 7  Q5 How can you avoid hypoglycemia (drop in blood sugar level)? • follow the diet • respect the doses of prescribed drugs Q6 How should this hypoglycemia be corrected?? • piece of sugar or candy | |  |
|  | **sub total 3** | **3** |
|  |  |  |
| 8 | **feet** |  |
| Q1 The diabetic can walk barefoot. (false) Q2 The diabetic must every night check inside the the soles of his feet before sleeping (true) Q3 If a wound appears on the diabetic's foot, he has to make himself immediately a bandage (false)  Q4 Wounds or pain in the feet require consultation (true) Q5 The diabetic must protect his feet against the maceration, heat sources and sharp objects (true) | |  |
|  | **sub total 4** | **1** |
|  | **lifestyle** |  |
| 9 | **exercise** |  |
| Q1 A diabetic needs to spend some time walking   per day (true) Q2 Older people should avoid walking in order not to have heart problems false) Q3 How much time per day should you do   physical activity every day? • 30mn | |  |
| **Sous total 5** | | **1** |
| Q4 Can you classify these physical activities: Low Moderate Important Q4-1 Clean house • Moderate Q4-2 Driving • low Q4-3 Walking • important Q4-4 Work as a secretary • low Q4-5 Run • important Q4-6 Dancing important | |  |
|  | **sub total 6** | **1** |
| 10 | **Diet** |  |
| Q1 Eating a lot is a sign of health (false) Q2 Fats are as harmful as fast sugars  for the body (true) Q3 A diabetic can eat fruits (true) Q4 Diabetics can eat all cereals (true) Q5 Diabetics should only eat fonio (false) Q6 The diabetic must eat salad and vegetables (true) Q7 Cite 2 starchy foods Q8 Cite 2 dairy products Q9 Cite 2 fruits and 2 vegetables Q10 Cite 2 fat products Q11 Give 2 sweet products Q12 Why should you eat less salty? • HTA problem • to prevent cardiovascular problems  Q13 Which foods should we control to eat less salty? • the canned goods Q14 Why should you eat less fat? • to prevent cardiovascular problems Q15 Which foods should we control to eat less fat? Q16 Why should you eat less sweet? • To avoid the complications of diabetes Q17 Which foods should we control to eat less sweet? Q18 Which foods should compose a balanced meal? • Starchy foods • Fruits • Dairy products • Meat and fish • Water Q19 A diabetic must nibble between meals • No  Haut du formulaire | |  |
|  | **sub total 7** | **1** |
