# Supplementary material for: Structured peer-led diabetes self-management and support in a low-income country: The ST2EP randomised controlled trial in Mali
Source: PLoS One. 2018 Jan 22;13(1):e0191262. doi: 10.1371/journal.pone.0191262 (PMC5777645; doi:10.1371/journal.pone.0191262)
Supplement: S2 File — French protocol Version 1 (v1) and Version 2 (v2); English translation of the protocol (v2); Acceptation letter from the Malian Ethical Comitee. (ZIP) [file pone.0191262.s002.zip › S2_file/Acceptation_letter.pdf]

D. DICKO

MINISTERE DE L'ENSEIGNEMENT
SUPERIEUR ET DE LA RECHERCHE SCIENTIFIQUE

▸▸▸ **UNIVERSITE DE BAMAKO** ▸▸▸

FACULTE DE MEDECINE DE PHARMACIE ET
D'ODONTO-STOMATOLOGIE / BP 1805

☎ : (223) 20 22 52 77 | 🖷 : (223) 20 22 96 58

BAMAKO – MALI

N°2011__55__/FMPOS

Le Président du Comité
D'Ethique de la FMPOS

Bamako, le 14 juin 2011

/-) **Monsieur le Directeur de l'ONG "Santé Diabète Mali"**

Cher Directeur,

J'ai le plaisir de vous informer que votre projet de recherche intitulé : **«Essai comparatif randomisé de l'apport d'une intervention éducative structurée par des pairs sur l'amélioration de l'HbA1c chez des patients diabétiques de type 2 dans la Commune 1 du District de Bamako au Mali»** a été examiné par le Comité d'Ethique de la Faculté de Médecine, de Pharmacie et d'Odonto-Stomatologie de l'Université de Bamako au cours de sa séance du samedi 11 juin 2011.

Le Comité d'Ethique a décidé de vous donner son accord pour l'exécution de vos travaux car vous avez satisfait aux conditions suivantes :

1. Fournir le résumé.
2. Fournir la liste des sigles et abréviations.
3. Mode opératoire de l'appareil DCA 2000.
4. Protocole d'accord avec les pairs éducateurs.
5. Reprendre les contacts du Comité d'Ethique FMPOS.

Le Comité d'Ethique de la FMPOS vous souhaite plein succès dans vos recherches.

LE PRESIDENT
DU COMITE D'ETHIQUE

Le President

Prof. Mamadou Marouf KEITA

Comité d'Ethique de la FMPOS
