# Supplementary material for: Structured peer-led diabetes self-management and support in a low-income country: The ST2EP randomised controlled trial in Mali
Source: PLoS One. 2018 Jan 22;13(1):e0191262. doi: 10.1371/journal.pone.0191262 (PMC5777645; doi:10.1371/journal.pone.0191262)
Supplement: S2 File — French protocol Version 1 (v1) and Version 2 (v2); English translation of the protocol (v2); Acceptation letter from the Malian Ethical Comitee. (ZIP) [file pone.0191262.s002.zip › S2_file/Protocol_English_v2.pdf]

Malian Ethical committee:

Research project

« A randomized trial of an intensive education intervention using a network of involved diabetic patients (peer educators) to improve glycemic control of type 2 diabetic patients »

HISTORY OF PROTOCOL UPDATES

VERSION	DATE	RAISON DE LA MISE À JOUR
**1.0**	**June 2011**	**Version submitted to the Malian ethical committee, Bamako**
**2.0**	**2016/03/13**	**Error corrected in the paragraph page 11 (Sample size calculation)**: It is assumed that in the reference group, the value of HbA1c is 8.5% and with a **standard deviation of 2.0**.

ONG Santé Diabète Délégation au Mali
Hippodrome rue 237 porte 122 – BP 2736 - Bamako (Mali)
0022320219566 – contact@santediabete.org

1. Introduction

I - Principal Investigator for the project

Surname : Besançon<br>*MSc Biologie, Nutrition et physiopathologie de la nutrition (specialized in developing countries)*

First Name(s): Stéphane

Name of Institution/organization: NGO Santé Diabète Mali (SDM)

Position in the Institution/organization: Program director NGO Santé Diabète Mali

Street Address : Hippodrome - rue 254 porte 69

Post/Zip Code: BP 2736	City/Province: Bamako

Country: Mali

Tel: + 00 223 20 21 95 66	Fax: + 00 223 20 21 95 66

Mobile: +00 223 77 81 26 70	Email: santediabetemali@wanadoo.fr

PI (Stéphane Besançon) and Co-PI (Pr Sidibe) Mali

Stéphane Besançon is a biologist, nutritionist specialized in pathophysiology and nutrition, specialist for developing countries. After a research carried out in Mali in 2000, with the International Agronomic Research Centre for Development (CIRAD) on a cereal, fonio (Digitaria Exilis), and diabetes, he founded in 2001, the NGO Santé Diabète Mali and became its programs director in 2003. Since 2003, with the NGO Santé Diabète Mali, he multiplies researches on the problem of diabetes mellitus in Africa and projects to improve prevention and care of diabetes mellitus in Mali. His international expertise has enabled him to take part in various projects led by the International Diabetes Federation (IDF) and the World Health Organization (WHO). This expertise also allowed him to conduct international expertise to support various governments (Mali, Guinea Conakry, Burkina Faso, Madagascar…) to write or implement the national policy of prevention and fight against diabetes.

In Mali, Stéphane Besancon, in close collaboration with Professor Sidibe (head of the endocrinology and diabetes unit at the national hospital Bamako), has conducted many research projects and assessment of the impact of actions on Malian diabetic patients. He also led several projects aimed at improving prevention and care of diabetes mellitus in Mali.

Finally, he developed a strong expertise in projects involving peer educators. Indeed, since 2004 he has developed in Mali a methodology of primary prevention of risk factors for chronic non-communicable diseases using a network of peers educators. This pilot methodology has been replicated in several areas of Mali. It will be evaluated before publication at the end of 2009.

Other investigator (s)*s*

Santé Diabète

Pr Sidibé Traoré (Pr aggregate of endocrinology, Head of unit of endocrinology and diabetology national hospital of Bamako)
Hôpital national du Point G – Unité d'endocrinologie et de diabétologie – Bamako – Mali
Cell : (223) 672 21 79 – sidibe2050@yahoo.fr

Dr Maryvette Balcou-Debussche, PhD of Sociology and Educational Sciences, PAEDI [Process of Actions in Education: Determinants and Impacts], EA Equipe d'accueil 4281. IUFM-Université d'Auvergne. 36, avenue Jean-Jaurès 63407 Chamalières Cedex, France

Dr Xavier Debussche – MD, MSc, Head of Depatment, Endocrinology and Metabolism, Regional research coordinator – CH Felix Guyon – CHR de La Réunion
97405 St Denis Cedex (Réunion and dependencies) - x.debussche@wanadoo.fr

Dr xavier Debussche and Dr Maryvette debussche

The scientific work of Maryvette Balcou-Debussche questions the construction, socialization and the contextualization of knowledge in various fields: adults in training, health professionals, chronic disease. Focused on knowledge differentiated reports and social variations of these reports, the main research has resulted in several scientific articles, chapters in collective books and two major books: one on the training of health professionals, The other on the education of patients suffering of chronic disease. The ethno sociologic approach of the author is requested for several development projects in France and abroad. Designer and director of the collection « Nids d'apprentissage », she developed an integrative, pragmatic and theoretically located approach of the therapeutic education (Ile de la Reunion, France, Mauritius, Burundi), in close articulation with the training of health professionals. She works with the INPES on several projects, including the publication of a book on the skills of professionals in therapeutic education, to be published in October 2008.

Referral person for the pole Chronic Pathologies and Metabolic Diseases and responsible for the research at the CHR of Ile de la Reunion, Xavier Debussche is carrier of several projects. He took part in epidemiological research (REDIA-1 and 2, REDIA-PREV2) which gave rise to several publications in scientific journals. Focused on the research of coherence and quality in the care course of patient, he participated actively in the development of care networks for patients presenting cardiovascular risk (Ile de la Reunion, Mauritius, Burundi) articulating the actions led in hospital and physicians in town, medical care and built and continuing education approach. Board Member of the ALFEDIAM and member of the ANCRED (Association of Diabetes networks in France), he is involved in the structuring of health care networks and in the search for a global integrative approach of the patient suffering of a chronic disease. Current projects focus on studying the feasibility of educational activities and their impact, crossing the quantitative and qualitative approaches, in collaboration with ethno sociologists and health psychologists.

Name and postal address of the main institution/organization involved

Name of Institution/organization: NGO Santé Diabète Mali (SDM)

Street Address : Hippodrome - rue 254 porte 69 – BP2736 - Bamako

Country:Mali

Tel: + 00 223 20 21 95 66 Fax: + 00 223 20 21 95 66

Mobile: +00 223 77 81 26 70 Email: santediabetemali@wanadoo.fr

Website (if any): www.santediabetemali.org

II - Describe the main institution/organization responsible for the project

NGO "Appui au développement, Santé Diabetes Mali (SDM)" was created in 2001, following the first work completed, in Mali, with the International Centre in Agronomic research for Development (CIRAD), on the diabetes Mellitus in Mali. SDM is an association of international solidarity. SDM is a non governmental organization (NGO), is a sustainable development association favouring projects that use a participative step.
SDM works in very close partnership with the Ministry of Health, the national direction of health, medical specialists and associations of diabetic patients in Mali. SDM is recognized and supported by the Malian government. This support was officialized by the signature of a master/ executive agreement with the Malian government on September 23, 2003.
"Appui au développement, Santé diabète Mali" is implementing different projects in Mali to improve the management of diabetes in Mali:

- Prevention of diabetes « Sensitizing, information of the public Malian on the diabetes Mellitus» will have as its aim to increase awareness and inform the general population about diabetes, its causes, consequences and how it can be treated. This will be done by using various tools to inform, educate and communicate specific messages to improve the knowledge of the general public and community and family leaders. One axis target specifically awareness in schools
-Training health professional and care restructuring: this axe is designed to train health professional to decentralize the management of diabetes in 5 regions in Mali. It is also aimed at providing the drug and testing equipment to ensure that these consultations may be decentralized.
- "Strengthening the associations of diabetic patients." This axe aims to boost existing diabetic patients associations and to form associations in areas with decentralized care for diabetes and without associations.

In addition to these axis, the NGO Santé Diabète Mali has developed several research projects on the problem of diabetes in Mali. This research has concerned:
- Research on the property of food eaten in Mali
- Research on the impact of traditional medicines
- Research on metabolic complications in the long term for the ARV
- Development of work on the economic impact of diabetes

Our NGO currently manages more than 15 contracts with various international donors including the European Union and various international cooperation agencies. Our NGO has therefore the organization, procedure manuals and mechanisms for the activities monitoring and the management control needed.

The NGO Santé Diabète Mali (SDM) has very deep relations with the International Diabetes Federation. SDM has organized workshops for the IDF Africa Region (example: in 2006 SDM has organized the seminar on policies to fight against diabetes in Africa that gathered 75 participants from 17 African countries). It also takes part in several projects for the IDF (guideline for africa, economic impact study).

III - Name and postal address of other supporting institution(s)/ organization(s) involved

Name: National hospital Point G

Street Address: Unit of endocrinology and diabetology – Bamako (Mali)

Email: sidibe2050@yahoo.fr - Tel: +(223) 66 72 21 79

IV - Describe the supporting institutions

National hospital Point G – endocrinology and diabetology unit – Bamako – Mali
The hospital will realize the implementation and monitoring of the project in Mali in collaboration with the NGO Santé Diabète Mali (SDM). The strict collaboration between national hospital and University Hospital will permit to have support by the epidemiology department of the Faculty for the processing and data analysis

PAEDI Research Center – Clermont-Ferrand – France PAEDI [Process of Actions in Education: Determinants and Impacts] works on adult learning and context influences: professionalisation, practices' analysis, learning in plri-linguistic context, intergration of sociocultural, psychological, and language specificities in learning. In the field of Health and patient education, PAEDI works on the differentiated access to knowledge and educational tools in chronic diseases and the problematics of social variations, low literacy and precarity. .This team has developed the methodology of education using the ”Learning Nests” and will support the university hospital in Bamako and the NGO Santé Diabète Mali to adapt the tools to the Malian and the development of this approach in health education.

CHR of La Reunion island - Endocrinology and Metabolism (Felix Guyon Hospital) Endocrinology and Diabetology in La Reunion
Dr Xavier Debussche and his team has implemented in different outpatient settings (in La Reunion, France and Mauritius) the development of self management courses for patients, using the Learning Nests approach intimately coupled with medical care and follow-up. This team brings the expert support in diabetes, training and organization of the implementation of actions in diabetes education.

2. Non-technical summary

The project will test the implementation of a specific methodology for education of type 2 diabetic patients that will be set up using patients involved (peer educators) and 3 guides developed specifically for the therapeutic education of type 2 diabetic patients. This project will take place in 1 sites in mali : the capital Bamako. 75 diabetic patients will be subjected to intervention with this methodology and 75 other diabetic patients will be the control group. At various stages of the project, we will analyze the impact on biological, anthropological constants, etc ... of the group undergoing the intervention compared to the control group.

3. Scientific abstract of proposed study

Title of project:
A randomized trial of an intensive education intervention using a network of involved diabetic patients (peer educators) to improve glycemic control of type 2 diabetic patients

Research question (s)/hypothesis:
Main objective of the research: compare the improvement of HbA1c 1 year after an intervention led by peer educators, versus a conventional care in health centre
Secondary objectives:
Study the evolution of bio clinical parameters: fasting glucose, weight, BMI, blood pressure, waist size

ONG Santé Diabète Délégation au Mali
Hippodrome rue 237 porte 122 – BP 2736 - Bamako (Mali)
0022320219566 – contact@santediabete.org

Method (s) :
This study will be conducted by a multidisciplinary team that brings together high level skills in research projects and in peer education projects. It will be conducted in one site in one country Mali. This site was chosen because they meet a functional care, access to medicines and a dynamic association of diabetic patients. Peer educators and persons targeted by the project will be recruited through the local association of patients. This association will be a social support that provides on going prevention counseling, education and support services to people who have diabetes. It represents an important link between patients, educators and general medical services. The procedure will start by the training of peer educators who were identified and recruited to facilitate the sessions Then, the 4 educational sessions: control of cardiovascular risk (blood pressure, waist circumference, smoking, cholesterol, glycaemia), control of Food (balance, fat, carbohydrates), physical activity management, insulin management, will be carried out and patients will be able to:
- Analyze knowledge that appears in various forms, including forms adapted to illiterate patients (colour codes, presence of photos)
- Act on knowledge: the patient observes, makes hypothesis, he experiments, compares, deduces, analyzes, makes relations.
- Work in interaction with other learners, which gives education a social dimension.
- Decide on the implementation of actions taking into account individual, cultural, social, economic context

For each situation, a day of training will be conducted by trained peer educators with theoretical contributions on the issue, practical scenarios, analyzes of the issues out of frame and put in "educational security ". These training days will be implemented with 8-10 patients (1Hour and an half) and the 4 sessions will take place over a period of two months starting from the inclusion of patients.

2 groups of 75 persons will be formed. One group will have specific education using methodology of learning nests. The other group will be a control group not subject to this specific education.
To evaluate, we propose a classical randomized controlled experimental (RCT) design, with randomization at the person-level. For outcome measures, we propose change in HbA1c, increase of social and emotional support and increase of linkage to clinical care. In addition to HbA1c, we propose measuring changes in systolic, diastolic blood pressure, and weight.

Public health significance : It is very important to find an education methodology adapted to the African context. This line of diabetes management is essential to improve the management of African diabetic patients.

Sustainability plan: If the evaluation of this methodology is conclusive, the Malian state will implement the methodology in the 22 diabetes units of the country.

4. Details of application

Main objective
Compare the improvement of HbA1c 1 year after an intervention led by peer educators during 12 months associated with conventional care, versus standard care alone in clinic, for 120 people with type 2 diabetes in Sikasso Mali.

Secondary objectives
1 - To compare the intermediate evolution of the HbA1c (3, 6 and 12 months)
2 - To compare the evolution of the bio-clinical parameters at 3.6, and 12 months: weight, BMI, blood pressure, waist measurement

ONG Santé Diabète Délégation au Mali
Hippodrome rue 237 porte 122 – BP 2736 - Bamako (Mali)
0022320219566 – contact@santediabete.org

Background information about project including current evidence in the field and literature and Indicate how the project addresses the community's need for diabetes care prevention.
Education on diabetes is known through several names: 'education of the patient', ' patient's therapeutic education ', 'self management education' etc…[1]. All these terms question about the diabetic person's place in a continuous progress involving permanent communication with a care team and the coordination of a care program [2]. Therapeutic education is a necessary component of an integrated care process for people with diabetes. Diabetes is a complex condition, which requires an effective medical management from caregivers and a good autonomous management from the person with diabetes. This dual approach helps to promote choice of healthy lifestyles, improve the quality of life and reduce direct and indirect health costs for society [3]. Educational approaches set up must recognize the critical and fundamental role of psychosocial factors for effective diabetes education [4], [5]. In 1914, Elliott Joslin published a book in which he stressed the importance of training nurses to participate to the education on diabetes and its management [6]. In 1936, insulin appeared and specialized nursing in diabetes had expanded. It became clear that nurses should develop their skills when it comes to education and counselling as well as expertise in clinical care [7]. The publication of the DCCT studies results in 1993 and UKPDS in 1998 amended the diabetes care, from the simple reduction of glycaemia and blood pressure, to the active prevention of complications and in to the intensification of treatment [12] [13]. It was a turning point in the role of diabetes educator. In developed countries, diabetes educators began by considering the key aspects of the insulin doses adaptation, of the changes in eating habits and of the screening for complications, paving the way for the work of specialised nurses and of other caregivers, such as dieticians, chiropodist in diabetes education [8]. Several studies have highlighted, in developed countries, the challenge for health professionals to implement education activities for patients. Above the consideration of the different cultural contexts, the barriers often described by professionals for the establishment of effective patient education are the availability of time and the inadequacy of the initial and continuing training [9]. At hospital, the particular context in which education situations took place do not necessarily encourage the effective setting up of prevention and health practice in every day life [10][15]. In the context of African health systems that combine low human and financial resources with a very important turn over of staff, it is very difficult to establish specific curricula for diabetes educators, but also to mobilize health staff specifically for this task [16]. In these particular settings, the involvement of peers in the assistance in daily management and living with diabetes, the Social and emotional support and the Linkage to clinical care seems to be a perfect tighten to complement the organization of diabetes care in these countries. The reviews of several studies have shown the very positive results of this approach that can be implemented by following several methodological models [11] [12] [13]. For this study we have chosen to implement and evaluate the impact of the methodology: The Learning Nests approach: Group Education focusing on understanding of key concepts and interactions with social context [14] [15] [16]. This approach developed by health professionals and human sciences seems to be the best suited for the socio economic and cultural contexts which are encountered in Africa. It is based on 5 principles- the building and setting up of learning situations which are based on scientific data of the medical and social/human sciences frame (socioconstructivism, knowledge construction, adult learning principles): - The integration of the context of life in developing the educational situation - The work on the operational dimension (use of the knowledge for the action, decision making by the learner), with the support of individual booklets (for the teacher and for the learner) -The intimate coupling of the educators training and the educational situations for learners - The implementation in a long-term program, with precise indicators of results

Aims: To evaluate the effectiveness and sustainability of diabetic patient-based (peer educators) education program in Bamako (Mali) using a randomized trial, with 150 type 2 diabetic patients

Global method: the purpose of this methodology is to set up coordinated training and education activities targeting populations with diabetes based on a pragmatic approach (the learning nests methodology) of appropriation and construction of knowledge that takes into account the individual, social, economic, cultural context. This work is being implemented on the basis of group modules

ONG Santé Diabète Délégation au Mali
Hippodrome rue 237 porte 122 – BP 2736 - Bamako (Mali)
0022320219566 – contact@santediabete.org

specifically designed in their running: are studied the concrete elements of knowledge to mobilize, patients' actions, the role of health the educator, indicators of progress and monitoring during sessions and on the long term. Each patient is gotten to consider the elements involved in his/her disease, and the actions that can be achieved taking into account the feasibility in his/her own context. The education monitoring is envisaged through a data compendium present in the individual booklets that are given to patients at the end of each education session. The animations sessions will allow:
- Patients to appropriate knowledge that is essential to better manage chronic disease.
- Patients to understand diabetes and reduce cardiovascular risk by learning how to manage their treatment, diet and regular physical expenditure.
- Patients to stake and analyse the elements of their environment, their representations and ordinary habits with the knowledge coming from the medical field. This analysis of elements in presence should allow everyone to take relevant and appropriate decisions (family, culture, economic, social network).

In practice the implementation of the methodology takes place in 4 stages:
1 - The training of peer educators who have been identified and recruited to lead the animations
2 - An initial cycle on the themes of knowledge and control of cardiovascular risk (BP, cholesterol, blood sugar, waist measurement, diabetes, treatment and complications).
3- An initial cycle on the subject of food controlling (balance, fat, carbohydrates).
4- An initial cycle on the themes of physical activity.

During each education session, interactive workshops will allow to work on: a balanced diet, carbohydrates in the diet, cardiovascular risk and understanding of diabetes. For each situation, a one-day training will be conducted by trained peer educators with theoretical input on the issue dealt with, put in practice situation, analyses of issues outside the framework and put in "safety education". These training days will be implemented with 8-10 patients (1H 30) and 3 meetings will take place over a period of 2 to 3 months. An educational replay will be made 6 months after, 12 months after and 18 months after. The educational booklets will be available for patients at the end each meeting. It is important to note that the tools used in this methodology allow for accessibility of learning by people with low literacy rates. It allows also the reproducibility of the education sessions because these sessions are designed and supervised, with brochures for patients, guides for teachers, and indicators of learning during the course.

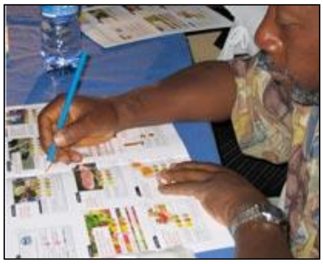

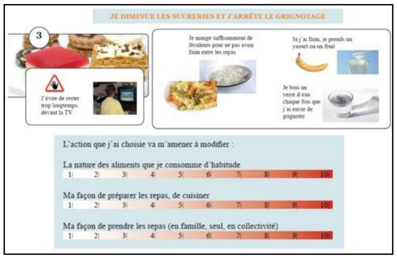

Sessions' stakes:

Cognitive stakes
- Distinguish variables on which we can (not) act
- Identify the decisive variables and the difference of their impact on the CVD
- Evaluate one own RCV from personal data (BP, HDL…)

Activities of the learners:
=> Observation
=> Experimentations
=> Comparisons, analyses
=> Awareness acquiring by everyone of the gap
(between the outcome and the optimized total health)
=> Setting relationship with the weakening of the atheroma
=> Calculation of total health (risk RCV)
=> Comparison to a optimized total health and to the total of other patients
=> Measure of the impact of the different actions on the variables CVR.
=> Choice of 2 actions (food, AP) potentially realizable.
=> Awareness of the possibility of increasing the total health
=> Reflection on the realization of actions, in context (social…)
=> Choice or not to keep the actions chosen and followed during 5 years
=> Performance indicators for each learner

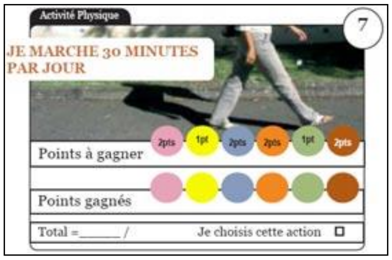

The implementation of this comprehensive methodology, including the selection of action areas, the recruitment of educators as well as patients and the implementation of education through the learning nests, will allow a support by:

ONG Santé Diabète Délégation au Mali
Hippodrome rue 237 porte 122 – BP 2736 - Bamako (Mali)
0022320219566 – contact@santediabete.org

Santé Diabète

1 • Assistance in daily management and living with diabetes : the provision for patients of supports understandable and discussed in situation facilitates the appropriation of knowledge over time: the written materials are discovered, approached and negotiated in situation, with the help of the educator. The patient takes the written materials at home, and keeps them carefully for the possibility of organizing the monitoring and the daily management of his diabetes and his life over the long term.
2 • Social and emotional support: the methodology of patients and educators recruitment through patients' associations and educational clubs will provide a very strong social and emotional support to patients. The group education methodology will also allow patients to support each other. Indeed, the work in interaction with other students, gives education a dimension allowing a very strong emotional support. The possibility of keeping the written material negotiated and worked in situation (booklets), will disseminate information within the family or/and in the social network of the trained patient (without risk of knowledge transformation, as the booklet is a reference). This possibility increases the number of people informed on the prevention of chronic diseases, prevention of complications but also allow bringing the necessary information to the family and social network of the formed patient which will strengthen the social and emotional support brought to the patient.
3 • Linkage to clinical care: The choice of intervention areas for Mali has been done taking into account the areas where the care of patients is complete with: an effective and operational diabetes consultation, medicines available and a dynamic diabetic patients association. In these areas, the approach is integrated. The addition of the education methodology by the educators will represent another step in the care pyramid of diabetes. All health workers of the area will be involved in the implementing and monitoring of the process. In addition to the role of educator, educators will also be trained to accompany patients in health facilities that manage diabetes in the intervention area. All peer educators will receive a tool representing the circuit of the patient in the intervention area and will give them to each patient during the animations. Finally, after the meetings patients may accompany each other in the health system.

Methods for the randomization
The patients will be selected from the list of diabetic patients followed in diabetes consultations of Bamako.
From this numbered list, a selection will be will randomly conducted with a number table to constitute the intervention group. We will verify that all the patients randomly selected to form this group meet the inclusion criteria of the group (see below).
The randomization list will be kept by the principal researchers of the survey.

Eligibility criteria
The study will be conducted among patients with type 2 diabetes followed in the decentralized diabetes units of Bamako. The methodology used to choose patients is described in the paragraph above. The recruitment will last during four months.

Inclusion criteria
- Patients followed in the diabetes units of the project area and doing regular consultations
- Patients with type 2 diabetes or treated or not with insulin
- Poorly controlled diabetes with HbA1c ≥ 8%
- Patients who accepted to undergo the whole process of peer education
- Patients who agreed to perform all biological measures included in the protocol
- Patients aged from 30 to 70 years old

Non-inclusion criteria
- Diabetic patients not carrying out their monitoring in diabetes units of the intervention area
- Patients followed in the diabetes units of the intervention area but not realizing their consultations regularly
- Patients with type 1 diabetes
- Severe complications within the preceding 3 months: infection, coronary complications, severe renal failure

ONG Santé Diabète Délégation au Mali
Hippodrome rue 237 porte 122 – BP 2736 - Bamako (Mali)
0022320219566 – contact@santediabete.org

- Concomitant illnesses threatening the functional or vital prognosis

Comparison group
The group subjected to educations will follow the whole process of peer education described in Part 1 of this Protocol.
The control group will perform these "classic" individual consultations but will not follow the whole process of peer education.
The classical management in diabetes consultations consists of:
- A counselling session
- A measure of blood glucose
- A measurement of blood pressure
- A measure of weight and size
- A complete clinical examination
- A prescription or a renewal of treatment (diabetes pills, insulin, IEC, statins etc ...)

Main judgment criteria
The main judgement criteria on which the analysis will be done to comply with the superiority of the contribution of a group educational approach by the peer will be the evolution of the HbA1c from the inclusion to 1 year:
- the HbA1C gives a reliable reflection of the glycemic control that is not subject to short-term variations or bias caused by the social aspects that may influence self-evaluation of data;
- it responds directly to all practices that peer education aims to change (diet and exercise, self glucose monitoring, proper use of drugs etc ...)
- It is the most important physiological parameter to improve for people with diabetes, as it is related to the probability of occurrence of micro-and macro vascular complications.

For the measurement of glycated hemoglobin, the apparatus used will be the machine “Infopia”. The preservation of the reagent throughout the study will be precisely monitored to avoid, with high temperatures, deterioration of reagents and therefore invalid action. The reagents will be stored in reagents storage structures of the laboratories of the health facilities involved. The measures will be undertaken by the laboratory assistants of the structures involved in the project and who will have been beforehand trained.

SECONDARY JUDGMENT CRITERIA
The secondary judgement criteria will be of 2 types:

- Clinical and biological criteria:
☐ intermediate evolution of the HbA1c, between 0, 3 months, 6 months and 12 months
☐ evolution at T = 0, 3 months, 6 months and 12 months of the systolic and diastolic blood pressure, weight, BMI and waist measurement.

To measure the blood pressure, armbands of different sizes (normal and obese) are available. The adequate armband will be placed on the upper arm and inflated with a manometer, to measure blood pressure using a stethoscope placed in the hollow of the arm on the artery. The systolic and diastolic pressure will be measured. The blood pressure will be measured on the right arm after a rest period of 5 min. The average of the two measurements taken at two minute intervals will be used.
To measure the weight, the scale 'seca' will be placed on a wooden board. As it is not possible to ask the patient to undress, we will estimate the weight of his/her clothes and it will be subtracted from the recorded measurement on the survey form.
To measure the size, a portable measuring apparatus will be installed on a flat surface. Patients must remove their shoes and their chech (headscarf). The legs will be joined and heels placed against the back wall of the apparatus. The patient should stand up straight and look ahead.
All equipment used will be new and in duplicate. This will enable us to ensure a quality control of the measures conformity, comparing the results between the two copies of the same equipment.

ONG Santé Diabète Délégation au Mali
Hippodrome rue 237 porte 122 – BP 2736 - Bamako (Mali)
0022320219566 – contact@santediabete.org

Santé Diabète

The waist measurement will be done with reference methods using a simple tape measure. Clear the abdomen of the client from clothing and accessories. Ask the client to stand up, with feet at the width of shoulder and arms folded across chest without tension. Place one knee on the floor on the right side of the client. The waist is measured at the upper edge of the iliac crest. To locate this bony landmark, palpate the upper part of the right hip until you locate the upper edge of the iliac crest. Mark a horizontal line to mark the midline of the body. Place the ribbon around the waist so that the bottom edge of the ribbon is aligned with the horizontal line. Using the technique of folded arms; superpose the tape held in one hand above the zero shown on the tape held into your other hand. Make sure the ribbon is aligned in a horizontal plane around the waist. Apply enough tension on the ribbon to keep it straight, without pressing into the flesh of the abdomen. At the end of a normal expiration, take the measure to the nearest 5 mm.

CALCULATION OF THE SIZE OF STUDY
In this trial, the patients were randomized into 2 groups:
- Group 1: group educational intervention by peer educators associated with classical management
- Group 2: classical management without peer education

This is a randomized trial comparing open impact on HbA1c of a 1 year peer education compared to a conventional monitoring alone in health centre.
The comparison group is on HbA1c, the primary judgement criteria. The test is a test of superiority.
It is assumed that in the reference group, the value of HbA1c is 8.5% and with a **standard deviation of 2.0**, (previous study conducted in the framework of the NGO Santé Diabète Mali under publication) and we consider that the strategy would be interesting if the assessed value of the mean HbA1c decreased by 1 percentage point of HbA1c and the standard deviation of 0.5 .
In this situation, with a risk α of 5% and a power 1-β of 80%, we must include 60 patients in each group.
in order to take into account a proportion up to 20% of patients lost of sight, we decided to recruit 75 patients per group that is to say 150 patients in total.

STATISTICAL METHODS USED
The data will be analyzed by a medical epidemiologist with the Epi Info software.
Initially, we will proceed to the description of the included patients and compare the characteristics of both groups of patients to detect any differences at inclusion. These characteristics are expressed as percentage for qualitative variables and as mean and median and their dispersions parameters, respectively, standard deviation and interquartile 25 and 75. The comparison of characteristics between the two groups will be performed using appropriate tests, the exact X2 or Student's t-test. Then, a uni-variable analysis of variance analysis type will be conducted to identify variables to include in the multivariable explanatory models. The inclusion threshold chosen will be 0.25. Data will be expressed as means ± SD unless indicated otherwise. Comparisons within groups were performed using analysis of variance by comparing the averages using the comparison test of averages or the Student's t-test.
The comparison between groups will be conducted by analysis of variance, using the Bonferroni correction where appropriate. The results will be statistically significant at $p < 0.05$.

Direct beneficiaries in Mali: The 150 diabetic patients directly reached by the activities / The 15 peer educators trained in the action areas selected in Mali. The 25 health agents of the action areas selected in Mali / Families and relatives of patients reached by animations (10 000 persons)
Indirect beneficiaries in Mali : All the diabetic patients of Mali (3% of the adult population) / All the health workers involved in the management of diabetes in Mali / The Malian health systems / The Malian population by reducing the risk factors of diabetes and increasing the quality of life
Indirect beneficiaries in Africa: Diabetic patients of French and English speaking African countries which will benefit from the methodology and tools developed

ONG Santé Diabète Délégation au Mali
Hippodrome rue 237 porte 122 – BP 2736 - Bamako (Mali)
0022320219566 – contact@santediabete.org

Santé Diabète

Sustainability plan: The project implements an inclusive approach in existing systems. Ability building will be targeting diabetic people of the country and professionals of the public service included in the health pyramid. The institutionalization of the project will be done through the capitalization of the action in the national programs of prevention and fight against diabetes and in national research programs. Strengthening will be provided to local partners through training of its members to project management and raise funds so that the partner can invest in the sustainability of the action. A protocol for monitoring and evaluation was prepared for the results of that action can be validated and published. The expertise at the regional level will enable the IDF Africa to develop a sub regional strategy, this project represents an opportunity to bring new financial and technical partners on this issue.

Chronogram

	Month 1 - 5	Month 6	Month 7	Month 8	Month 9	Month 10	Month 11	Month 12
	Preparation	Randomization	Pre inclusion T-X (*)	Inclusion T 0	Visit T 3	Visite T 6	Visite T 12	
Identification of patients	✓	✓						
Patient Education experts	✓							
Informed Consent			✓					
Clinical examination			✓	✓	✓	✓	✓	
Laboratory tests			✓	✓	✓	✓	✓	
Knowledge			✓	✓	✓	✓	✓	
Data Processing								✓
Report								✓

Bibliography

[5] D'Ivernois JF, Gagnayre R. Health education and therapeutic education: what kind of specialised education and training are needed?
Sante Publique. 2006 Sep;18(3):351-2
[6] Assal JP, Jacquemet S, Morel Y. The added value of therapy in diabetes: the education of patients for self-management of their disease. Metabolism 199; 1(Suppl 1): 61- 4.
[7] Peyrot M, Rubin RR, Lauritzen T, et al; on behalf of the International DAWN Advisory Panel. Psychosocial problems and barriers to improved diabetes management: results of the Cross-National Diabetes Attitudes, Wishes and Needs (DAWN) Study. Diabet Med 005; 10: 139-85.
[8] Mulcahy K, Maryniuk M, Peeples M, et al. Diabetes self-management education core outcomes measures. Diabetes Educ 003; 5: 68-0, 3-84, 8-8 passim.
[9]Balcou-Debussche M, editor. L’éducation des malades chroniques. Une approche ethnosociologique. Paris: Éditions des archives contemporaines; 2006.
[10]Allen N. The History of Diabetes Nursing, 1914-1936. The Diabetes Educator 2003; 9: 96-89.
[11] Diabetes Control and Complications Trial/Epidemiology of Diabetes Interventions and Complications Study Research Group, Jacobson AM, Musen G, Ryan CM, Silvers N, Cleary P, Waberski B, Burwood A, Weinger K, Bayless M, Dahms W, Harth J. Long-term effect of diabetes and its treatment on cognitive function. N Engl J Med. 2007 May 3;356(18):1842-52.
[12] Nicollerat JA.Implications of the United Kingdom Prospective Diabetes Study (UKPDS) results on patient management. Diabetes Educ. 2000 Nov-Dec;26 Suppl:8-10.
[13]Dunning P. The diabetes educator: evolution of a nurse specialist. Practical Diabetes 199; 6:220-2.
[14]Balcou-Debussche M, Debussche X. Type 2 diabetes patient education in Reunion Island: Perceptions and needs of professionals in advance of the initiation of a primary care management network. Diabetes&Metab. (2008), doi:10.1016/j.diabet.2008.03.002

[15]Balcou-Debussche M., Debussche X. Hospitalisation for type 2 diabetes: The effects of the suspension of reality on patients, subsequent management of their condition. Qualitative Health Research, accepted for publication.
[16] Beran, D. Besançon. S. Report of the International Insulin Foundation on the assessment protocol for insulin access in Mali. Bamako: International Insulin Foundation, 2004.
[17] Feeny DH, Furlong WJ, Boyle M, Torrance GW. Multi-attribute health status classification systems: Health Utilities Index. Pharmacoeconomics. 1995;7:490-502.
[18] Furlong WJ, Feeny DH, Torrance GW, Barr RD. The Health Utilities Index (HUI) system fo rassessing health-related quality of life in clinical studies. Ann Med. 2001;33:375-384.
[19] Torrance GW, Feeny DH, Furlong WJ, Barr RD, Zhang Y, Wang Q. Multi-attribute preference functions for a comprehensive health status classification system: Health Utilities Index Mark 2. Medical Care. 1996;34:702-722.
[20] Sadana R, Mathers CD, Lopez AD, Murray CJL, and Iberg KM. Comparative analysis of more than 50 household surveys on health status (abridged version). 12-23-2000. Geneva, Global Programme on Evidence for Health Policy, World Health Organization.
[21] Hibbard JH, Mahoney ER, Stockard J, Tusler M. Development and testing of a short form of the Patient Activation Measure. Health Services Research. 2005;40:1918-1930.

5. Risk management procedures

Study of the social risks and benefits
This study will enroll human subjects in a controlled experiment that seeks to substantially alter the knowledge, feelings, behavior and health of persons who are assigned to active treatment.
For all subjects, the study will collect and store sensitive personal information and expose every subject to the risks and discomfort of blood tests.

Ethical consideration
The main ethical considerations in this study are the lack of access to peer education, randomly, of 50% of subjects of the study. While we hope that exposure to peer education is beneficial, we remain cautious about the concrete results of this action for diabetic people because such a program has never been tested before. If the evaluation of this study is conclusive, this methodology could significantly improve and prolong the lives of hundreds of thousands of diabetic people in Africa. Therefore, we believe it is ethical to refuse access to the program to half of the subjects in order to determine its value in sight to spread it to hundreds of thousands of diabetic people in Africa.

6. Potential risks to participants and co

Risk minimization

The risk of breach of confidentiality and the risks linked to biological tests are minimal, as long as measures are taken to maintain data security and avoid the risk of infections and pain during samplings. To ensure confidentiality, all the study data will be stored separately and the files will be protected by password.
Only people with an obvious need for data access, such as PI, will have the passwords. To strengthen protection measures, data forms will be stored in protected cabinets. It will be important to encode these forms in order to avoid displaying data such as names, addresses, phone numbers and other personal identification data. Only the principal researcher, the co-researchers and the analysts will have access to keys linking the study identifiers to respondents' personal data. The survey forms will be kept for 5 years after the conclusion of the project to enable data validation, and then destroyed.

ONG Santé Diabète Délégation au Mali
Hippodrome rue 237 porte 122 – BP 2736 - Bamako (Mali)
0022320219566 – contact@santediabete.org

To minimize the risks associated with the samplings, only new and sterile equipment with disposable needles will be used.
To minimize the risks directly associated with the intervention of peer education, subjects must be fully informed about all aspects of the intervention. This information will be done through an informed consent form. All subjects will be required to sign this declaration of consent as a condition of participation in the study. In order not to exclude the illiterate people, the consent document will be read aloud to all subjects and translated by a translator for people who do not speak French. Subjects will be encouraged to ask questions and will sign the consent only after they have asked all the questions they have.
The participation of subjects will be entirely voluntary. The right of access to medical care will not be affected by the participation or not to this study.
Topics will be able to withdraw at any time.
Before starting the study, the team will have the full receipt of the ethics committee of the Faculty of Medicine, Pharmacy and Dental Medicine in Mali (FMPOS). This receipt will ensure that all commitments will be reviewed, approved and monitored by a national ethics committee. The recruitment of subjects will not begin before the reception of this receipt.

Information form (in French)

Cette étude a pour objectif de mesurer l'impact biologique et anthropométrique d'une intensification de l'éducation de patients diabétiques de type 2.
Je suis informé à travers ce document et la lecture qu'il m'en est fait que :
- le diabète (ou maladie du sucre) est une maladie chronique, dont les principaux symptômes sont une fatigue excessive, une envie d'uriner régulière, une perte de poids subite, une envie de boire et de manger excessive. La prise en charge optimale de cette maladie associe de l'activité physique régulière, un régime alimentaire adapté et la prise de médicament comme des antidiabétiques oraux ou de l'insuline.
- l'hypertension est également une maladie chronique souvent due à un régime alimentaire riche en sels. Les symptômes sont des maux de tête, des vertiges, etc… Une prise en charge optimale associe un régime alimentaire moins riche en sels, une perte de poids en cas d'obésité et une prise de médicaments antihypertenseurs.
- qu'une piqûre au bout du doigt (pouvant produire une très légère douleur de quelques secondes) sera réalisée afin de prélever quelques gouttes de sang pour mesurer la glycémie à jeun et l'hémoglobine glyquée
- qu'un brassard sera gonflé pendant quelques secondes sur le haut de mon bras afin de mesurer la tension artérielle, cela ne provoquant aucune douleur,
- que les mesures tour de taille et tour de hanche seront réalisées par une femme pour les femmes et par un homme pour les hommes.
- qu'il existe un médecin au sein de l'équipe du Csréf de la commune 1 du district de Bamako à qui je peux m'adresser en cas de problèmes de santé.
- que cette étude pourra comporter le risque de découvrir une complication de mon diabète inconnue de moi-même. Mon bénéfice étant qu'en suivant le protocole d'éducation intensif je pourrai agir préventivement si un risque de développer une complication du diabète apparaît ou, avoir accès à une prise en charge optimale si un problème de complication du diabète est diagnostiqué.

Consent form (in French)

Formulaire de consentement libre et éclairé Fiche n° : ……

ONG Santé Diabète Délégation au Mali
Hippodrome rue 237 porte 122 – BP 2736 - Bamako (Mali)
0022320219566 – contact@santediabete.org

Santé Diabète

Mon nom est ……………………………………………………………. et je reconnais avoir été informé, dans la langue locale :

- des objectifs de l'étude intitulée «Essai comparatif randomisé de l'apport d'une intervention éducative structurée par des pairs sur l'amélioration de l'HbA1c chez des patients diabétiques de type 2 dans la région de Sikasso au Mali», réalisée par l'unité d'endocrinologie et de diabétologie du CHU du point G et de l'ONG Santé Diabète Mali (SDM).

- des bénéfices et des risques de cette étude

- que le responsable de l'étude est le Pr Assa Sidibé endocrinologue travaillant au CHU du point G (téléphone 66722179)

- que cette étude a été soumise pour validation au comité d'éthique de la faculté de médecine. Celui-ci peut être contacté via le secrétaire principal Pr Idrissa A. Cisse (76132011) ou le président Pr Mamadou M. Keita au numéro (66722022)

- que les résultats de cette étude seront gardés confidentielles.

- que les mesures biologiques seront réalisées gratuitement et qu'il n'y aura pas de compensation financière d'aucune sorte.

En connaissance de cause, j'accepte de participer à l'étude et donc de fournir toutes les informations nécessaires au formulaire d'enquête et permettre à l'équipe responsable de l'enquête de mesurer mon poids, ma taille, mon tour de taille/tour de hanche, ma glycémie, mon hémoglobine glyquée ainsi que ma pression artérielle.

Fait à Bamako Le 17/06/2011

Signature (ou empreinte) Le responsable de l'étude

ONG Santé Diabète Délégation au Mali
Hippodrome rue 237 porte 122 – BP 2736 - Bamako (Mali)
0022320219566 – contact@santediabete.org
