# Supplementary material for: Structured peer-led diabetes self-management and support in a low-income country: The ST2EP randomised controlled trial in Mali
Source: PLoS One. 2018 Jan 22;13(1):e0191262. doi: 10.1371/journal.pone.0191262 (PMC5777645; doi:10.1371/journal.pone.0191262)
Supplement: S2 File — French protocol Version 1 (v1) and Version 2 (v2); English translation of the protocol (v2); Acceptation letter from the Malian Ethical Comitee. (ZIP) [file pone.0191262.s002.zip › S2_file/Protocol_French_v2.pdf]

Centre hospitalo-universitaire (CHU) du point G
Service de Médecine Interne
Unité de diabétologie et d'endocrinologie
B.P. 609
Bamako, Mali

« Essai comparatif randomisé de l'apport d'une intervention éducative structurée par des pairs sur l'amélioration de l'HbA1c chez des patients diabétiques de type 2 dans la commune 1 du district de Bamako au Mali»

Version N° 2
Du 13/03/2016

Chercheur principal : Pr Sidibe assa Traore

Sponsor : bourse de recherche Bridges de la Fédération Internationale du Diabète (FID)

Etude menée en 2011 et 2012

HISTORIQUE DES MISES À JOUR DU PROTOCOLE

VERSION	DATE	RAISON DE LA MISE À JOUR
**1.0**	**Juin 2011**	**Version soumise au Comité d'éthique du Mali (Bamako)**
**2.0**	**13-03-2016**	**Correction du** paragraphe « calcul de taille de l'échantillon » (page 7) qui comportait une erreur et a été corrigé avec les éléments de calcul suivants :<br>On fait l'hypothèse que, dans le groupe de référence, la valeur de l'HbA1c est de 8,5 % avec un écart type de 2.0. et on considère que la stratégie évaluée serait intéressante si la valeur de l'HbA1c moyenne diminuait de 1 point de pourcentage d'HbA1c.

SOMMAIRE

RESUME DE L'ETUDE 3
I – DESCRIPTION DU PROJET 4
II - OBJECTIFS DE LA RECHERCHE 5
1 – OBJECTIF PRINCIPAL 5
2 – OBJECTIFS SECONDAIRES 5
III - METHODOLOGIE DE LA RECHERCHE 5
1 - CONCEPTION DE LA RECHERCHE 5
2 – SCHEMA DE LA RECHERCHE 6
3 – ZONE D'INTERVENTION 6
4 – POPULATION D'ETUDE 6
4.1 - critères d'Éligibilité 6
4.2 Méthodes pour la Randomisation 7
4.3 - Calcul de la taille de l'échantillon 7
5 – DEROULEMENT DE LA RECHERCHE 7
5.1 – Calendrier de la recherche 7
5.2 – Description des méthodes d'intervention 7
5.3 – Conduite de suivi 11
5.4 – Critères de jugement 11
6– ANALYSE ET GESTION DES RESULTATS 13
6.1 Méthodes statistiques employées 13
6.2 Surveillance de la recherche 13
6.3 Contrôle et assurance qualité 13
7– CONSIDERATIONS ETHIQUES 14
7.1 Approbation par le comité d'éthique et consentement éclairé des participants à l'étude 14
IV – DESCRIPTION DU GROUPE DE RECHERCHE 15
V - BIBLIOGRAPHIE 16
VI – FORMULAIRE DE CONSENTEMENT INDIVIDUEL 17
Formulaire de consentement libre et éclairé 18
VII - BUDGET 19
Annexe 1 : CV : 21

ABREVIATIONS

OMS : organisation mondiale de la santé
Cscom : centre de santé communautaire
Csref : centre de santé de référence
PMA : paquet minimum d'activités
IEC : information et éducation par la communication
DRS : direction régionale de la santé
SDM : ONG « Santé Diabète Mali »
IMC : indice de masse corporelle
TT : tour de taille
TH : tour de hanche
RTH : rapport tour de taille/tour de hanche
Hba1c : hémoglobine Glyquée
HTA : hypertension
DCCT : Diabetes Control and Complications Trial
UKPDS : UK Prospective Diabetes Study
RCV : risque cardiovasculaire
PVD : pays en voie de développement
FID : fédération internationale du diabète

RESUME DE L'ETUDE

TITRE

Essai comparatif randomisé de l'apport d'une intervention éducative structurée par des pairs sur l'amélioration de l'HbA1c chez des patients diabétiques de type 2 dans la commune 1 du district de Bamako au Mali

Nom et coordonnées du chercheur principal et des chercheurs associes

Chercheur principal :
Pr Sidibé Traoré (Pr agrégé en endocrinologie, chef de l'unité d'endocrinologie et de diabétologie de l'hôpital national du point G)
Hôpital national du Point G – Unité d'endocrinologie et de diabétologie – Bamako – Mali
Tel : (223) 66 72 21 79 – sidibe2050@yahoo.fr

***Chercheurs associés*:**
Mr Besancon Stéphane (MSc Biologie, Nutrition et physiopathologie de la nutrition (spécialisé pour les pays en voie de développement)
ONG Santé Diabète Mali - Bp 2736 - Bamako – Mali
Tel: (223) 66 76 86 85 - santediabetemali@wanadoo.fr / site: www.santediabetemali.org

Dr Nientao Ibrahim (Diabétologue)
Centre National de Lutte Contre le Diabète – Quartier du fleuve – Bamako (Mali)
Tel : (223) 66 72 99 49 - ibnientao@yahoo.fr

Partenaires en appui / expertise :

Dr Maryvette Balcou-Debussche, PhD of Sociology and Educational Sciences, PAEDI [Process of Actions in Education: Determinants and Impacts], EA Equipe d'accueil 4281. IUFM-Université d'Auvergne. 36, avenue Jean-Jaurès 63407 Chamalières Cedex, France

maryvette.balcou@wanadoo.fr

Dr Xavier Debussche – MD, MSc, Chef de department endocrinology et métabolisme,Coordinateur régional de recherche – CH Felix Guyon – CHR de La Réunion
97405 St Denis Cedex (Réunion) - x.debussche@wanadoo.fr

Dr Daou Sissoko
1, Route de Montgaillard
97400 Saint Denis, La Réunion, France
Tél : 05 47 74 84 48
daouda.sissoko@gmail.com

Pr Serge Halimi
CHU de Grenoble – service d'endicrinologie diabétologie
Avenue de Kimberley - 38130 Echirolles
SHalimi@chu-grenoble.fr

I – DESCRIPTION DU PROJET

Le diabète n'est plus aujourd'hui une maladie des pays et des hommes riches. L'Organisation mondiale de la Santé (OMS), estime qu'on passera de 171 millions de personnes atteintes en 2000 à 366 millions en 2030, soit une prévalence de 2,8 % à 4,4 % dans l'hypothèse favorable où l'obésité ne progresse pas. Les PVD compteront alors 76 % des patients diabétiques dans le monde. Cette croissance a justifié le rapport alarmant publié par l'OMS en 2005 pour une mobilisation rapide sur ces nouveaux besoins de santé. L'accès aux soins, au traitement, à l'éducation et à la prise en charge des complications liées au diabète est un axe essentiel de la résolution votée aux Nations-Unies en décembre 2006 sur le diabète pour éviter que les personnes diabétiques ne connaissent une situation d'exclusion et une vulnérabilité accrue.
Dans les PVD, l'explosion de l'épidémie de diabète, ainsi que des autres maladies chroniques, est la conséquence directe de l'apparition massive du surpoids et de l'obésité. Ce sont principalement les villes de ces pays qui sont touchées. En Afrique, une femme sur quatre et un homme sur six sont touchés en milieu urbain, taux qui devient inférieur à 10 % en milieu rural. Pour les PVD, cet accroissement majeur de l'obésité qui implique autant les classes aisées que les classes populaires, est essentiellement relié à l'apparition d'une transition nutritionnelle progressive définie comme une modification progressive des régimes alimentaires, avec notamment une nette augmentation de la consommation de matières grasses d'origine animale. Enfin l'accroissement de l'obésité dans les pays du sud, donc de l'apparition de maladies chroniques comme le diabète, est renforcé par l'augmentation de l'espérance de vie, ainsi que par la croissance de la sédentarité.
L'éducation au diabète est connue sous plusieurs appellations : 'éducation au patient', 'formation à la gestion autonome du diabète' et 'éducation thérapeutique du patient'. Toutes ces expressions ont en commun la place de la personne atteinte de diabète dans une intervention permanente impliquant la communication avec une équipe de soins et la coordination d'un programme de prise en charge. L'éducation au diabète devient de plus en plus un modèle de soins pour les personnes atteintes de diabète. En effet, le diabète est une condition complexe, qui nécessite une gestion médicale efficace de la part des prestataires de soins et une bonne gestion autonome de la part de la personne atteinte de diabète. Cette double approche permet de promouvoir des choix de styles de vie sains, d'améliorer la qualité de vie et de réduire les coûts de santé directs et indirects pour la société. Les approches éducatives mises en place doivent reconnaître le rôle critique et fondamental des facteurs psychosociaux pour une éducation au diabète efficace.
En 1914, Elliott Joslin publiait The Treatment of Diabetes Mellitus: With Observations Upon the Disease Based Upon One Thousand Cases (9), un ouvrage dans lequel il insistait sur l'importance de former le

personnel infirmier à participer à l'éducation au diabète et à sa gestion. En 1936, l'insuline avait fait son apparition et les soins infirmiers spécialisés en diabète s'étaient élargis. Il devenait évident que le personnel infirmier devait développer ses compétences en matière d'éducation et de conseil ainsi que son expertise dans les soins cliniques. La publication des résultats des études DCCT (Diabetes Control and Complications Trial) en 1993 et UKPDS (UK Prospective Diabetes Study) en 1998 a modifié les soins du diabète, passant de la simple réduction des taux de glycémie et de la pression artérielle, à la prévention active des complications et à l'intensification du traitement. Cela a été un tournant dans le rôle de l'éducateur en diabète. Dans les pays développés, les éducateurs en diabète ont commencé à prendre en charge des aspects clés de l'adaptation des doses d'insuline, de la modification des habitudes alimentaires et du dépistage des complications, préparant le terrain pour les rôles des infirmiers de pratique avancée et d'autres prestataires de soins, comme les diététiciens, les podologues, les psychologues spécialisés en éducation au diabète.

De nombreuses études ont mis en évidence, dans les pays développés, la difficulté pour les professionnels de santé de mettre en œuvre des activités d'éducation du patient. Les barrières souvent décrites par les professionnels pour la mise en place effective d'éducation du patient sont la disponibilité en temps, les différences des contextes culturels et l'inadaptation de la formation initiale et continue. Dans le contexte des systèmes de santé Africains qui cumulent de faibles ressources humaines et financières avec un turn over très important du personnel, il est très difficile de mettre en place des curriculums spécifiques pour des éducateurs diabète, mais aussi de pouvoir mobiliser des personnels de santé uniquement pour cette tâche. Dans ces cadres particuliers l'implication des pairs dans l'Assistance à la gestion quotidienne du diabète, au soutien social et émotionnel support en lien avec les cliniques semble être un ancrage parfait pour compléter l'organisation de la prise en charge du diabète dans ces pays. Les revues de différentes études ont montré les résultats très positifs de cette approche qui peut être implémentée suivant de très nombreux modèles méthodologiques.
Pour cette étude nous avons choisi de mettre en œuvre et d'évaluer l'impact de la méthodologie : des nids d'apprentissage. Cette méthodologie développée par des professionnels de santé et des sciences humaines semble être bien adaptée pour les contextes socio économiques et culturels que l'on rencontre en afrique. Elle est basée sur 4 principes :
- L'utilisation d'un modèle théorique construit sur une approche socio-constructrice et à partir des principes de l'apprentissage des adultes
- L'intégration du contexte de vie dans l'élaboration de la situation éducative
- L'intime couplage de la formation des éducateurs et des situations éducatives pour les apprenants
- La mise en œuvre dans un programme à court terme

II - OBJECTIFS DE LA RECHERCHE

1 – OBJECTIF PRINCIPAL

Comparer l'amélioration de l'Hba1c à 1 an après une intervention menée par des pairs éducateurs durant 12 mois associée à une prise en charge classique, contre une prise en charge classique seule en centre de santé, chez 150 personnes diabétiques de type 2 dans la commune 1 du district de Bamako, au Mali.
.

2 – OBJECTIFS SECONDAIRES

1 - Comparer l'évolution intermédiaire de l'HbA1c à 3, 6 mois et 12 mois
2 – Comparer l'évolution des paramètres biocliniques (poids, IMC, Pression artérielle, tour de taille) à 3, 6 et 12 mois
3 - Etudier l'évolution de la qualité de vie et de l'état de santé par questionnaires validés

III - METHODOLOGIE DE LA RECHERCHE

1 - CONCEPTION DE LA RECHERCHE

Compte tenu de la valeur que peut avoir l'éducation par les pairs pour les 240 millions de personnes atteintes de diabète dans le monde dont la grande majorité d'entre elles vivent dans les pays en voie de développement, il serait très dommage de perdre l'occasion d'évaluer le potentiel de l'éducation par les pairs en Afrique, en utilisant un modèle qui n'est pas immédiatement convainquant pour toutes les parties concernées (chercheurs, organismes donateurs, gouvernement etc.…) à qui il sera demandé, par la suite, de

financer une grande partie de la future mise en place de l'éducation par les pairs. Une validation scientifique de ces résultats par les cliniciens, les donateurs et les dirigeants africains ne pourra venir que de la publication dans un journal scientifique reconnu qui exige donc le modèle expérimental le plus fort possible.

2 – SCHEMA DE LA RECHERCHE

Pour cette étude nous avons choisi un essai clinique comparatif randomisé au niveau de la personne.
Ce modèle assurera la plus grande validité interne. La randomisation par site d'étude est trop risquée en raison des changements inattendus au niveau du personnel soignant, des ressources disponibles, de la disponibilité des médicaments et de la compétition entre les programmes de santé. Il n'est jamais possible de corriger ces changements après qu'ils se soient produits. Un modèle de randomisation au niveau de la personne est possible au Mali. En effet, dans le site choisi, nous avons déjà atteint le bon fonctionnement d'un système clinique de soins pour le diabète qui permet d'atteindre les directives de soins souhaités en Afrique (sur la base des directives régionales africaines sur le diabète et les directives de la Fédération Internationale du Diabète (FID) pour les pauvres et les pays en développement). Par conséquent, l'étape de construction du système de prise en charge du diabète, avec l'introduction d'un programme d'éducation par les pairs, peut être accomplie sans provoquer des contaminations croisées significatives. En effet, la création de groupes de soutien par les pairs ne causera pas de changements majeurs dans les prestations de soins de santé qui déborderont sur le groupe des sujets témoins. En outre, le temps de rencontre avec les médecins et leurs assistants est tellement limité dans le cadre des systèmes de prise en charge du diabète en Afrique, que nous ne prévoyons pas de possibilités pour les médecins de modifier le traitement des contrôles d'une manière compensatoire. Toutefois, pour éviter cela, nous allons former tous les soignants sur l'importance de maintenir la séparation expérimentale, et nous allons suivre tous les traitements liés au diabète pour détecter les éventuels biais qui pourraient apparaître.

3 – ZONE D'INTERVENTION

Le choix de la zone d'intervention pour le Mali s'est porté sur la commune 1 du district de Bamako. Ce choix a été fait en tenant compte de la possibilité de prise en charge complète des patients dans la commune avec : une consultation diabète effective et opérationnelle, les médicaments disponibles et une association de patients diabétique dynamique.

4 – POPULATION D'ETUDE

4.1 - critères d'Éligibilité

L'étude sera conduite auprès de patients diabétiques de type 2 suivis dans l'unité diabète décentralisée de la commune 1 du district de Bamako au Mali. La méthodologie de choix des patients est décrite dans le paragraphe ci-dessus. Le recrutement se déroulera durant les mois de juin et juillet 2011.

Critère d'inclusion

- Patients suivis dans l'unité diabète de la zone d'intervention et réalisant régulièrement leurs consultations ;
- Patients diabétiques de type 2 insulino-traités ou non
- Diabète mal contrôlé avec une HbA1c $\geq$ 8%
- Patients ayant acceptés de se soumettre à l'ensemble du processus d'éducation par les pairs
- Patients ayant accepté de réaliser toutes les mesures biologiques inclus dans le protocole
- Patients âgés de 30 à 80 ans

Critère de non inclusion

- Patients diabétiques ne réalisant pas leur suivi dans les unités diabète de la zone d'intervention ;
- Patients suivis dans les unités diabète de la zone d'intervention mais ne réalisant pas régulièrement leurs consultations ;
- Patients diabétiques de type 1 ;
- Complications évolutives sévères dans les 3 mois qui précèdent : infection, complication coronarienne, insuffisance rénale sévère ;

- Pathologies associées menaçant le pronostic fonctionnel ou vital ;
- Refus de participer à l'étude.

4.2 Méthodes pour la Randomisation

Les patients seront choisis dans la liste des patients diabétiques suivis dans les consultations diabète du CSREF de la commune.
A partir de cette liste numérotée un tirage au sort sera réalisé grâce à une table de nombre au hasard pour constituer le groupe d'intervention.
A partir de cette liste numérotée un tirage au sort sera réalisé grâce à une table de nombre au hasard pour constituer le groupe contrôle.
Nous vérifierons que tous les patients tirés au sort pour former ces groupes respectent les critères d'inclusion des groupes (cf. ci-dessous).
La liste de randomisation sera conservée par le chercheur principal de l'enquête.

4.3 - Calcul de la taille de l'échantillon

Dans cet essai, les patients sont randomisés en 2 groupes :
- groupe 1 : Intervention éducative de groupe par des pairs associée à la prise en charge classique
- groupe 2 : prise en charge classique sans éducation par les pairs

Il s'agit d'un essai randomisé ouvert comparant l'impact sur l'HbA1c d'une éducation par des pairs pendant 1 an par rapport à un suivi classique seul en centre de santé.

La comparaison des groupes porte sur l'HbA1c, critère de jugement principal. L'essai est un essai de supériorité.

On fait l'hypothèse que, dans le groupe de référence, la valeur de l'HbA1c est de 8,5 % avec un écart type de 2.0 et on considère que la stratégie évaluée serait intéressante si la valeur de l'HbA1c moyenne diminuait de 1 point de pourcentage d'HbA1c.
Dans cette situation, avec un risque α de 5% et une puissance 1-β de 80%, il faut inclure 60 patients dans chaque groupe.
Afin de tenir compte d'une proportion de patients perdus de vue pouvant atteindre 20%, on décide de recruter 75 patients par groupe, soient 150 patients au total.

5 – DEROULEMENT DE LA RECHERCHE

5.1 – Calendrier de la recherche

Les inclusions débuteront au mois de juin 2011.La période d'inclusion sera de 2 mois (juin 2011 à juillet 2011). Les patients participeront à l'étude durant 12 mois après cette inclusion.

5.2 – Description des méthodes d'intervention

<u>Intervention éducative de groupe par les pairs</u>

La première étape du processus sera le recrutement et la formation des pairs éducateurs.

Le recrutement des patients qui pourront devenir pairs éducateurs a été fait à partir de ces critères :
- Etre une personne atteinte de diabète (PAD) vivant à Bamako ;
- Etre suivi régulièrement par le médecin de l'unité diabète du de la commune 1 ;
- Etre prêt à assurer les séances d'éducation ;
- Pouvoir écrire et lire les lettres et les chiffres ;

- Avoir une bonne connaissance de la langue nationale de la région.

La phase initiale de formation passe par l'organisation d'un atelier de 3 jours de présentation et de travail sur les 4 livrets ainsi que regroupant les personnes atteintes de diabète pré sélectionnées. Suite à la première formation, les participants à la formation initiale maitrisant la méthodologie et les 4 livrets, seront invités à suivre le cycle de formation final pour devenir pairs éducateurs. Les 4 livrets apprenants (maitrise du risque cardiovasculaire, maitrise des graisses dans l'alimentation, et la gestion de l'activité physique) seront repris entièrement durant une journée par livret en langue nationale par le chef de projet IEC de l'ONG Santé Diabète Mali (SDM). Le chef de projet a été formé avant le démarrage du processus global.
Les animations tests sont l'étape finale de sélection et se déroulent en deux étapes:

Etape 1 :
Chaque patient pair éducateur animera une séance complète en utilisant les autres pairs éducateurs comme patients sur chaque livret. Cette mise en situation permettra au chef de projet de juger de la maitrise des outils mais aussi de la capacité à animer des séances complètes à partir des 3 livrets (maitrise du risque cardiovasculaire, maitrise des graisses dans l'alimentation, et la gestion de l'activité physique).

Etape 2 :
A la fin de ces 3 semaines qui auront permis à chaque patient pair éducateur d'animer une séance complète par livret, le chef de projet de l'ONG Santé Diabète Mali (SDM) retiendra en utilisant les grilles d'analyse les patients pairs éducateurs qui seront animateurs pour la suite de l'activité. 5 patients pairs éducateurs seront finalement retenus.

La mise en œuvre de l'approche par les nids d'apprentissage se déroule en 4 étapes :

1- Un cycle initial sur les thèmes de la connaissance et de la maîtrise de la « santé des artères » (glycémie, tension, cholestérol, tour de taille, traitements et complications) : les patients travaillent sur des éléments modifiables qui influent sur leur santé vasculaire, ils identifient les actions qui ont un effet positif sur ces éléments, en tenant compte des réalités de leur vie, afin de choisir une action réaliste à mettre en œuvre

2- Un cycle initial sur les thèmes de la maîtrise de l'alimentation (équilibre, graisses, glucides) : analyser et comparer les aliments contenant diverses quantités de lipides, identifier ceux contenant des acides gras saturés et non saturés, évaluer et corriger la quantité de lipides dans l'alimentation, si nécessaire, comprendre la relation entre les lipides, l'adiposité viscérale et le contrôle de la glycémie.

3- Un cycle initial sur les thèmes de l'activité physique. Il s'agit d'amener le patient à quantifier sa propre quantité d'activités physiques sur une base hebdomadaire, à comparer celle-ci aux recommandations, à analyser les conditions et la faisabilité d'une modification de la quantité d'activités physiques.

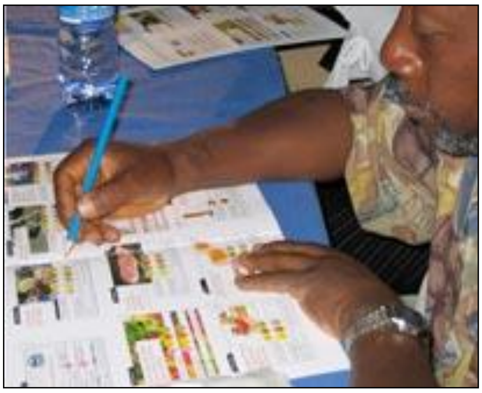

4 – Un cycle initial sur le thème de l'insuline**.** À travers deux séances complémentaires, les patients sont amenés à comprendre les enjeux de la mise sous insuline et à maîtriser les savoir-faire et savoirs être mobilisés dans la gestion du diabète de type 2 en contexte.[1] Le travail proposé lors des séances permet aux patients d'analyser les savoirs médicaux relatifs à la mise sous insuline ainsi que les dimensions psychosociologiques et contextuelles à prendre en compte.

Dans le cadre des 4 sessions d'éducation : maîtrise du risque cardiovasculaire (tension artérielle, tour de taille, tabac, cholestérol, glycémie), maîtrise de l'alimentation (équilibre, graisses, glucides), gestion de l'activité physique, gestion de l'insuline, les patients sont amenés à:

- analyser des connaissances qui apparaissent sous des formes diverses, dont certaines adaptées aux patients analphabètes et illettrés (codes de couleurs, présence de photographies)
- agir sur les savoirs : le patient observe, émet des hypothèses, expérimente, compare, déduit, analyse, met en relations.

[1] Voir « Education et prévention des malades chroniques » sur : http://www.archivescontemporaines.com/

- travailler en interaction avec d'autres apprenants, ce qui donne à l'éducation sa dimension sociale.

Lors de chaque session d'éducation, des ateliers interactifs permettront de travailler sur : l'équilibre alimentaire, les glucides dans l'alimentation, le risque cardiovasculaire, la compréhension de la maladie diabétique
Pour chaque situation, une journée de formation sera réalisée par les pairs éducateurs dûment formés avec des apports théoriques sur la question traitée, des mises en situation pratiques, des analyses des questions hors-cadre et des mises en « sécurité éducative ». Ces journées de formation seront mises en œuvre avec 8-10 patients (1H 30) et les 4 séances se dérouleront sur une période de 2 mois à partir de l'inclusion des patients.
Chaque patient sera soumis au cycle complet 5 fois durant les 12 mois d'intervention éducative. Les livrets éducatifs seront mis à disposition des patients en fin de séance.

Les enjeux des séances :

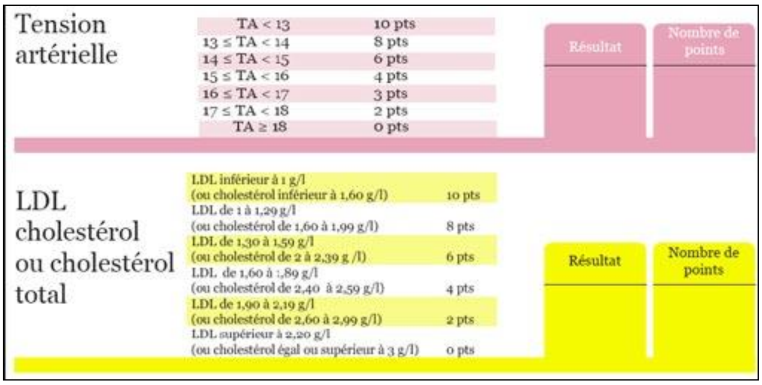

Enjeux cognitifs
- Distinguer les variables sur lesquelles on (ne) peut (pas) agir
- Repérer les variables déterminantes et leur différence d'impact sur le Risque Cardio Vasculaire (RCV)
- Évaluer son RCV à partir des données personnelles (TA, HDL…)

Activités des apprenants :
=> Observation,
=> Expérimentations,
=> Comparaisons, analyses,
=> Prise de conscience par chacun de l'écart (entre le résultat et le total santé optimisé),
=> Mise en relation avec la fragilisation de la plaque d'athérome,
=> Calcul du total santé (risque RCV),
=> Comparaison à un total santé optimisé et au total des autres patients

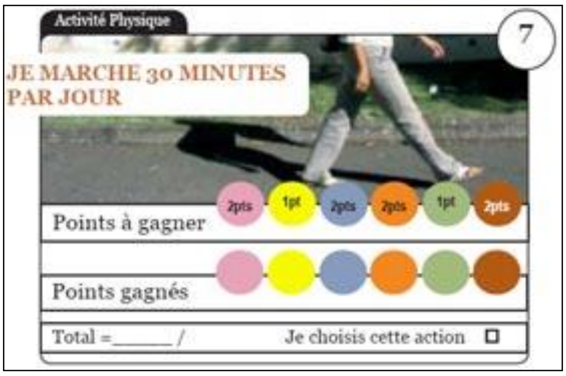

=> Mesure de l'impact de différentes actions sur les variables RCV.
=> Choix de 2 actions (alimentation, Activité Physique) potentiellement réalisables.
=> Prise de conscience de la possibilité d'augmenter le total santé

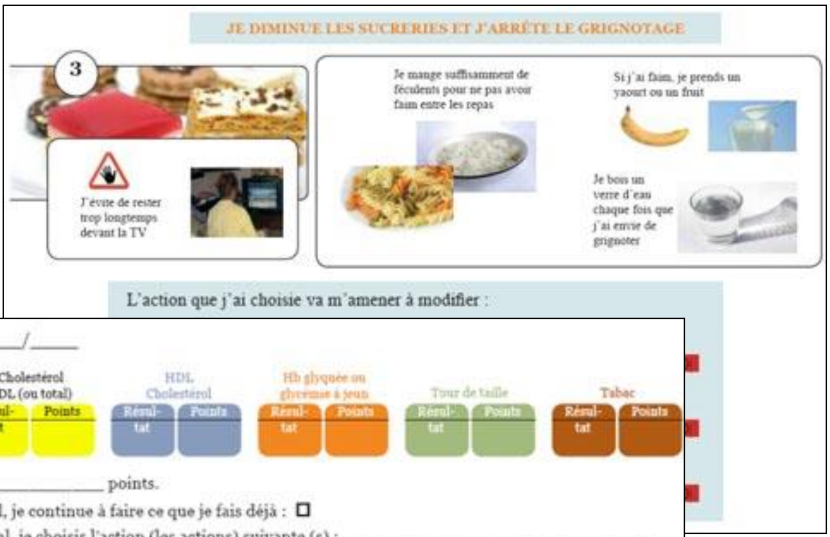

=> Réflexion sur l'opérationnalisation des actions, en contexte (social…)

=> Choix ou non de maintenir les actions choisies et leurs suivis sur 5 ans
=> Indicateurs de résultats pour chaque apprenant

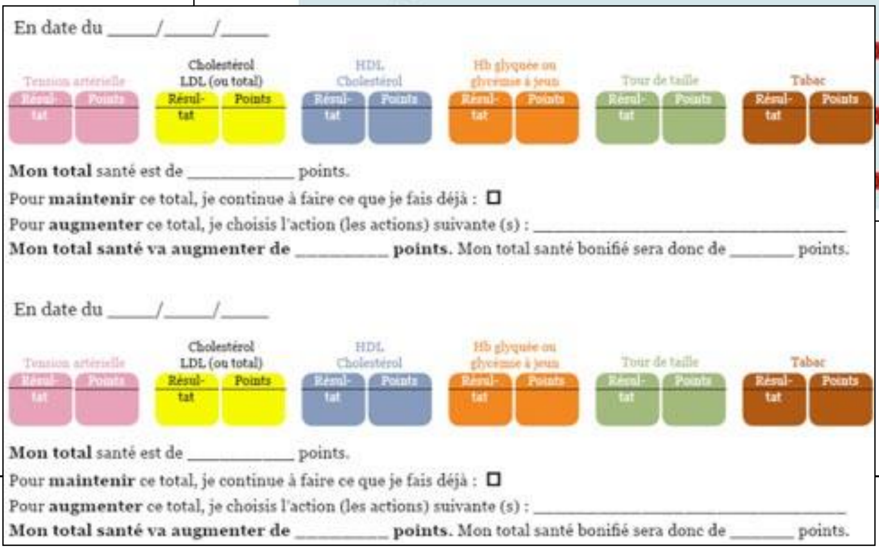

Synthèse

La mise en œuvre de cette approche par les nids d'apprentissage, comprenant le choix des zones d'action, le recrutement des pairs ainsi que des patients et la mise en œuvre des éducations grâce aux nids d'apprentissage, permettra une éducation par les pairs par :

1 • Assistance à la gestion quotidienne et la vie avec le diabète
La mise à disposition pour les patients de supports compréhensibles et discutés en situation facilite l'appropriation des connaissances dans la durée : les supports écrits sont découverts, appréhendés et négociés en situation avec l'aide de l'éducateur. Le patient emporte les supports écrits chez lui, et les conserve précieusement en vue d'organiser le suivi de la gestion quotidienne de son diabète et de sa vie sur le long terme.

2 • Soutien émotionnel et social
La méthode de recrutement des patients et des pairs à travers les associations de patients permettra d'apporter un soutien social et affectif très fort aux malades. L'approche par l'éducation de groupe permettra aussi aux patients de se soutenir mutuellement. En effet, le travaille en interaction avec d'autres apprenants, donne à l'éducation une dimension permettant un soutien affectif et émotionnel fort. La possibilité de conserver les supports écrits négociés et travaillés en situation (livrets), permettra de diffuser l'information à l'intérieur de la sphère familiale et ou dans le réseau social du patient formé (sans risque de transformation des savoirs, puisqu'ils restent en référence dans le livret). Cette possibilité multiplie le nombre de personnes sensibilisées à la prévention des maladies chroniques, à la prévention des complications mais aussi permet d'apporter l'information nécessaire à la sphère familiale et au réseau social du patient formé ce qui permettra de renforcer le soutien social et émotionnel apporté au patient.

3 • Lien avec les consultations diabète
Le choix de la zone d'intervention pour le Mali, la commune 1 du district de Bamako, a été fait en tenant compte de la possibilité de prise en charge complète des patients avec : une consultation diabète effective et opérationnelle, les médicaments disponibles et une association de patients diabétique dynamique. Dans cette zone, l'approche est intégrée. L'ajout de la méthodologie d'éducation par les pairs représentera une étape de plus dans cette pyramide de prise en charge du diabète. L'ensemble des agents de santé de la zone sera impliqué dans la réalisation et le suivi du processus. En plus du rôle d'éducateur, les pairs seront aussi formés pour accompagner les patients dans les structures sanitaires prenant en charge le diabète dans la zone d'intervention. Tous les pairs éducateurs recevront un outil représentant le circuit du malade dans la zone d'intervention qui sera distribué à chaque patient durant les animations. Enfin, les patients entrent eux après les séances pourront être des accompagnants les uns pour les autres dans le système de santé.

Groupe de comparaison

Le groupe soumis aux éducations suivra l'ensemble du processus d'éducation par les pairs décrit en partie 1 de ce protocole.
Le groupe témoin réalisera ces consultations individuelles « classiques » mais ne suivra pas l'ensemble du processus d'éducation par les pairs.

La prise en charge classique dans les consultations diabète se compose :

- d'une séance de conselling

- d'une mesure de la glycémie
- d'une mesure de la tension artérielle
- d'une mesure du poids et de la taille
- d'un examen clinique complet
- d'une prescription ou d'un renouvellement des traitements (Anti diabétiques oraux, insuline, Inhibiteurs de l'Enzyme de conversion, statine etc…)

5.3 – Conduite de suivi

En cas de rupture du suivi et ou du processus d'éducation une phase d'une semaine de relance et de recherche sera réalisée. En cas de rupture totale le patient sera exclu du protocole de recherche et considéré comme un perdu de vue.
En cas de complications sévères, d'hospitalisations longues ou de décès, les patients ne feront plus partie de l'étude.
A chaque arrêt définitif d'un participant une information complète sur cet arrêt sera réalisée et cette information sera transmise à tous les membres du comité de suivi de la recherche.
Ces patients arrêtés seront comptés avec les patients surnuméraires en cas d'arrêt ou de perdu de vue.

Visite de pré-inclusion

La visite de pré-inclusion est assurée par l'équipe décrite ci dessus. La visite de pré-inclusion aura lieu au mois de juin 2011. Durant cette visite l'équipe recueillera le consentement libre, éclairé et écrit du patient.

Visite d'inclusion et visites de suivi

La visite d'inclusion se fera dans les consultations diabète du CSReF de la commune 1 du district de Bamako. Cette visite d'inclusion comportera un examen clinique avec les mesures décrites précédemment, le bilan biologique décrit dans les chapitres précédents, la réalisation du questionnaire de connaissance et du questionnaire HUI3.
Les visites de suivi comporteront les mêmes éléments et se dérouleront à T= 3 mois, 6 mois et 12 mois.

Tableau récapitulatif de suivi patient

	Pré inclusion T-*X* (*)	Inclusion T 0	Visite T *3 mois*	Visite T *6 mois*	Visite T *12 mois*
Consentement éclairé	✓				
Examen clinique	✓	✓	✓	✓	✓
Bilan biologique	✓	✓	✓	✓	✓
Connaissances	✓	✓	✓	✓	✓

Toutes ces visites seront réalisées lors de consultations individuelles aux périodes données par une équipe comprenant un des chercheurs impliqués dans le projet, un médecin stagiaire de la faculté de médecine, de pharmacie et d'odontostomatologie (FMPOS) de la république du Mali et le médecin formant la consultation diabète de la commune 1 du district de Bamako.

5.4 – Critères de jugement

Critère principal de jugement

Le critère de jugement principal sur lequel portera l'analyse pour affirmer la supériorité de l'approche éducative de groupe par les pairs sera l'évolution du taux d'HbA1c entre l'inclusion et 1 an :

- l’Hba1c donne un reflet fiable du contrôle glycémique qui n'est pas soumise aux variations à court terme ou au biais causé par les aspects sociaux qui peuvent influencer l'autoévaluation des données ;
- l’Hba1c répond directement à l’ensemble des pratiques que l’éducation par les pairs a pour objectif de modifier (alimentation et l'exercice physique, auto surveillance glycémique, bonne utilisation des médicaments etc…)
- l’Hba1c est le paramètre physiologique le plus important à améliorer pour les personnes atteintes de diabète, car relié à la probabilité de survenue des complications micro- et macroangiopathiques.

Pour la mesure de l’hémoglobine glyquée, l’appareil utilisé sera le DCA 2000[2]. La conservation des réactifs tout au long de l’étude sera précisément surveillée pour éviter, avec les fortes chaleurs, la détérioration des réactifs et donc la non validité des mesures. Les réactifs seront conservés dans les structures de stockage des réactifs des laboratoires des structures de santé impliquées. Les mesures seront réalisées par les laborantins des structures impliquées dans le projet qui auront été préalablement formés.

Critères secondaires de jugement

Les critères de jugement secondaires sont de 2 ordres :

- ***critères clinico-biologiques*** :
    - évolution intermédiaire des taux d’HbA1c, entre 0, 3 mois, 6 mois et 12 mois
    - évolution de la pression artérielle systolique et diastolique, du poids, de l’IMC et du tour de taille à T=0, 3 mois, 6 mois et 12 mois

Pour la mesure de la tension artérielle, des brassards de taille différente (normale et obèse) seront disponibles. Le brassard adéquat sera disposé sur le haut du bras et gonflé avec un manomètre afin de pouvoir mesurer la tension artérielle à l’aide d’un stéthoscope placé dans le creux du bras au niveau de l’artère. La pression systolique et diastolique sera mesurée. La tension artérielle sera mesurée, au bras droit, suite à une période de repos de 5 min. La moyenne de deux mesures prises à deux minutes d’intervalle sera utilisée.

Pour la mesure du poids, le pèse personne « seca » sera posé sur une planche en bois. Comme il ne sera pas possible de demander au patient de se déshabiller, nous estimerons le poids des vêtements portés et celui-ci sera soustrait de la mesure notée sur la fiche d’enquête.

Pour la mesure de la taille, une toise portable sera installée sur une surface plane. Les patients devront enlever leurs chaussures ainsi que leur chech (foulard sur la tête). Les pieds seront joints et les talons disposés contre la paroi arrière de la toise. Le patient devra se tenir bien droit et regarder à l’horizon.

Tous les appareils utilisés seront neufs et en double exemplaire. Cela nous permettra d’assurer un contrôle qualité de la conformité des mesures, en comparant les résultats obtenus entre les deux exemplaires d’un même appareil.

Le tour de taille sera mesuré avec des méthodes de référence en utilisant un simple mètre ruban. Dégagez l’abdomen du client de tout vêtement et accessoire. Demandez au client de se tenir debout, les pieds à la largeur des épaules et les bras croisés sans tension devant la poitrine. Posez un genou par terre à la droite du client. Le tour de taille est mesuré au niveau du bord supérieur de la crête iliaque. Pour localiser ce repère osseux, palpez la partie supérieure de la hanche droite jusqu’à ce que vous localisiez le bord supérieur de la crête iliaque. Marquez d’un trait horizontal ce repère à la ligne médiane du corps. Placez le ruban autour de la taille de telle sorte que le bord inférieur du ruban soit aligné sur le trait horizontal. Au moyen de la technique des bras croisés, superposez le ruban tenu d’une main au-dessus du zéro indiqué sur le ruban tenu de l’autre main. Assurez-vous que le ruban soit aligné dans un plan horizontal autour de la taille. Appliquez assez de tension sur le ruban pour le maintenir droit. À la fin d’une expiration normale, prenez la mesure à 5 mm près.

[2] http://www.ascensia.ch/pub/fr/produits/dca2000.asp

6– ANALYSE ET GESTION DES RESULTATS

.

6.1 Méthodes statistiques employées

Les données seront analysées par un médecin épidémiologiste à l'aide du logiciel Epi info.
Dans un premier temps, nous procéderons à la description des patients inclus et comparerons les caractéristiques des deux groupes de patients afin de détecter d'éventuelles différences à l'inclusion. Ces caractéristiques seront exprimées sous forme de proportion pour les variables qualitatives et sous forme de moyennes et médians ainsi que leurs paramètres de dispersions, respectivement écart-type et interquartiles 25 et 75. La comparaison des caractéristiques entre les deux groupes sera effectuée à l'aide de tests appropriés, le X2 exact ou le test t de Student. Puis, une analyse univariable de type analyse variance sera conduite afin d'identifier les variables à inclure dans les modèles explicatifs multivariables. Le seuil d'inclusion retenu sera de 0,25. Les données seront exprimées en tant que moyen ± SD, sauf si indiqué autrement. Les comparaisons au sein de groupes ont été effectuées en utilisant l'analyse de la variance en comparant les moyennes grâce au test de comparaison des moyennes soit le test t de Student.
La comparaison entre les groupes sera effectuée par analyse de variance, en utilisant la correction de Bonferroni le cas échéant. Les résultats seront statistiquement significatifs à $p<0,05$.

6.2 Surveillance de la recherche

Un comité de pilotage sera crée pour suivre cette recherche avec le représentant du ministère de la santé en charge du programme national de lutte contre le diabète, un représentant du comité d'éthique, du groupe de recherche impliqué dans ce protocole et des médecins des consultations diabète de la ville de Bamako. Ce groupe se réunira trimestriellement pour suivre la progression et la validité des travaux effectués.

6.3 Contrôle et assurance qualité

6.3.1 Consignes pour le recueil des données

Toutes les informations requises par le protocole doivent être consignées sur des cahiers d'observation papiers (cf annexe) et une explication doit être apportée pour chaque donnée manquante. Les données devront être recueillies au fur et à mesure qu'elles sont obtenues, et transcrites dans ces cahiers de façon nette et lisible.
Les données erronées relevées sur les cahiers d'observation seront clairement barrées et les nouvelles données seront copiées, à côté de l'information barrée, accompagnées des initiales, de la date et éventuellement d'une justification par l'investigateur ou la personne autorisée qui aura fait la correction.

6.3.2 Suivi de la recherche

Le suivi de la recherche sera assuré par un coordinateur général qui sera chargé, auprès du chercheur principal de :

- la logistique et la surveillance de la recherche,
- l'établissement des rapports concernant son état d'avancement,
- la vérification de la mise à jour du cahier d'observation (demande d'informations complémentaires, corrections,…),
- la qualité des données recueillies dans le cahier d'observation : exactitude, données manquantes, cohérence des données avec les documents source,
- la gestion des disponibilités en matériels éducatifs.

6.3.3 Gestion des données

L'ensemble du protocole d'enquête (recueil des données, mesure biologique, questionnaire etc…) sera soumis à un pré-test avant le début de l'enquête, ceci afin d'optimaliser au mieux la réalisation de cette enquête. Les données issues des questionnaires seront enregistrées aux périodes choisies par les responsables de l'étude. Ces données seront vérifiées avant que les questionnaires rejoignent le site de saisie.

Les données biologiques seront remises par les laboratoires aux responsables de l'étude qui les vérifieront et les compileront avant que celles-ci rejoignent le site de saisie.
Les données de l'étude seront saisies et vérifiées (re-entrée) par une personne responsable de la saisie dans le logiciel Epi info. Les copies papiers des formulaires de l'étude seront conservées dans des armoires fermées à clés et sécurisées qui seront placées sous la surveillance du chercheur principal de l'étude à Bamako.

7– CONSIDERATIONS ETHIQUES

.

7.1 Approbation par le comité d'éthique et consentement éclairé des participants à l'étude

Etude des risques et des avantages sociaux.

Cette étude recrutera des sujets humains dans une expérience contrôlée qui vise à modifier sensiblement les connaissances, les sentiments, les comportements et la santé des personnes qui reçoivent un traitement éducatif actif.
Pour tous les sujets, l'étude permettra de recueillir et de stocker des informations personnelles sensibles et exposer tous les sujets à des risques et l'inconfort des analyses sanguines. Ainsi, toutes les informations utiles contenues dans le formulaire d'information seront données aux futurs participants pour requérir leur accord pour participer à l'étude.

Considération éthique

La principale considération éthique de cette étude est le non accès à l'éducation par les pairs, de manière aléatoire, de 50% des sujets de l'étude. Bien que nous espérions que l'exposition à l'éducation par les pairs sera bénéfique, nous restons prudents sur les résultats concrets de cette action pour les personnes atteintes de diabète car une telle approche n'a jamais été testé auparavant. Si l'évaluation de cette étude est un succès, cette méthodologie pourrait sensiblement améliorer et prolonger la vie de centaines de milliers de personnes atteintes de diabète en Afrique. Par conséquent, nous pensons qu'il est éthique de refuser, pour un départ, l'accès au programme de la moitié des sujets avec l'intention de déterminer sa valeur en vu de l'étendre à des centaines de milliers de personnes atteintes de diabète en Afrique. Les patients du groupe témoin seront à la fin du protocole les premiers à bénéficier des actions d'éducation.

Minimisation des risques

Les risques de violation de la confidentialité et les risques liés aux tests biologiques sont minimes, tant que des mesures sont prises pour maintenir la sécurité des données et d'éviter les risques d'infections et les douleurs lors des prélèvements. Pour assurer la confidentialité, toutes les données de l'étude seront stockées séparément et les fichiers seront sécurisés par mot de passe.
Seules les chercheurs principaux et associés auront accès aux données, tel que les chercheurs de l'étude, auront les mots de passe. Pour renforcer les mesures de protection, les formulaires de données seront stockés et sécurisés. Il est important que ces formulaires soient encodés et donc qu'ils ne présenteront ni noms, adresses, numéros de téléphone et d'autres données d'identification personnel. Seul le chercheur principal, les co-chercheurs et l'analyste auront accès à des clés qui relient les identifiants de l'étude aux données personnelles des enquêtés. Les données d'enquête seront conservées pendant 5 ans après la conclusion du projet pour permettre la validation des données, puis scellés.
Pour minimiser les risques associés aux prélèvements, seul le matériel neuf et stérile avec des aiguilles à usage unique sera utilisé.
Pour minimiser les risques liés directement à l'intervention de l'éducation par les pairs, les sujets devront être pleinement informés sur tous les aspects de l'intervention. Cette information se fera par l'intermédiaire d'un formulaire d'information qui restera en possession des participants. Il sera demandé à tous les patients de signer cette déclaration de consentement éclairé, comme condition de participation à l'étude. Pour ne pas exclure les personnes analphabètes, le document de consentement sera lu à voix haute pour tous les sujets et traduit dans la langue locale pour les personnes ne parlant pas français. Les sujets seront encouragés à poser des questions et signeront leur consentement éclairé, seulement une fois qu'ils estiment qu'ils n'ont plus d'autres questions à poser.
La participation des sujets sera entièrement volontaire et éclairée. Le droit d'accéder aux soins médicaux ne sera pas affecté par la participation ou la non-participation à cette étude.
Les sujets seront en mesure de se retirer à tout moment.
Avant le démarrage de l'étude, l'équipe devra avoir le quitus du comité d'éthique de la Faculté de Médecine, de Pharmacie et d'Odontostomatologie du Mali (FMPOS). Ce quitus fait obligation pour les

chercheurs de respecter tous engagements examinés, approuvés et suivis. Le recrutement des sujets ne pourra commencer tant que ce quitus écrit n'a pas été reçu.

IV – DESCRIPTION DU GROUPE DE RECHERCHE

Chercheurs principaux

Pr Sidibé : est professeur agrégée en endocrinologie et diabétologie. Après avoir exercé de nombreuses années au CHU de Marseille, elle rentre au Mali pour intégrer le service de médecine interne de l'Hôpital national du point G. En parallèle, elle assure l'enseignement de l'endocrinologie et de la diabétologie à la faculté de médecine, de pharmacie et d'odontostomatologie du Mali (FMPOS). Elle fait partie des rares spécialistes de sa discipline en Afrique. Cette expertise lui a permis de nombreux travaux reconnus internationalement. Aujourd'hui, le Pr Sidibé dirige l'unité d'endocrinologie et de diabétologie créée au sein du service de médecine interne de l'hôpital du point G et travaille sur la création d'un diplôme d'étude spécialisé en endocrinologie et diabétologie en Afrique de l'ouest, tout en développant une unité de pointe pour la prise en charge des patients et un pôle de recherche clinique sur le diabète.

Dr Ibrahim Nientao : est diabétologue. Il est le plus jeune spécialiste en diabétologie en Afrique de l'Ouest. Il travaille depuis 3 ans comme médecin consultant au centre national de lutte contre le diabète et pilote la décentralisation des soins du diabète mise en place par l'ONG Santé Diabète Mali en partenariat avec le gouvernement malien et l'unité du Pr Sidibé. Son expérience de clinicien, les travaux de recherche sur la prise en charge nutritionnelle des personnes atteintes de diabète et sur la décentralisation des soins du diabète au Mali lui a permis de devenir assistant de recherche sur de nombreux travaux internationaux menés en collaboration avec l'ONG Santé Diabète Mali (SDM). Il a pu présenter ses travaux dans différents colloques internationaux ou réaliser la présentation pays pour le Mali au forum de la CEDEAO sur la nutrition avec son travail sur les index glycémiques des aliments consommés au Mali. Aujourd'hui, il poursuit ses travaux de cliniciens et son appui pour la mise en place d'un système de prise en charge du diabète aux différents niveaux de la pyramide sanitaire du Mali

Stéphane Besançon : est biologiste et nutritionniste. Il possède, également, une spécialisation en physiopathologie de la nutrition et une en développement international. Après différents travaux en physiologie cardiovasculaire et sur la nutrition en Afrique, il fonde l'ONG Santé diabète Mali (SDM), structure dont il prendra la direction des programmes en 2003. Son expertise sur les systèmes de santé, les maladies chroniques et le diabète en Afrique lui permet de participer à de nombreuses expertises et rapports internationaux pour des gouvernements, des organisations internationales (OMS, IDF…) ou pour des campagnes internationales (unitesfordiabetes, rapport sur les maladies négligées au parlement européen …). Ses travaux sur les systèmes de santé, les maladies chroniques, la nutrition et le diabète en Afrique ont fait l'objet de nombreuses publications internationales. Aujourd'hui, il dirige l'ONG Santé Diabète Mali, poursuit ses travaux internationaux et a réalisé un ouvrage sur ces différentes problématiques qui a été publié en Février 2010.

Partenaires

Dr Maryvette Balcou-Debussche: Le travail scientifique de Maryvette Balcou-Debussche questionne la construction, la socialisation et la contextualisation des connaissances dans divers domaines: les adultes en formation, les professionnels de la santé, les maladies chroniques. Son axe de recherche principale a donné lieu à plusieurs articles scientifiques, chapitres dans des ouvrages collectifs et deux ouvrages majeurs: l'un sur la formation des professionnels de la santé, l'autre sur l'éducation des patients souffrant de maladies chroniques. L'approche ethno sociologique du Dr Balcou-Debuscche a été sollicitée pour plusieurs projets de développement en France et à l'étranger. Designer et directeur de la collection «EPMC (JNV d'apprentissage)», elle a développé une approche de l'éducation thérapeutique (Ile de la Réunion, France, Guyane, Ile Maurice, Burundi), en articulation étroite avec les formations des professionnels de la santé. Elle travaille avec l'INPES sur plusieurs projets, y compris la publication d'un livre sur les compétences des professionnels de l'éducation thérapeutique, publié en 2008.

Dr Xavier Debussche: référent pour le pôle pathologies chroniques et maladies métaboliques et responsable de la recherche au CHR de l'Ile de la Réunion, Xavier Debussche est porteur de plusieurs

projets. Il a pris part à la recherche épidémiologique (Redia-1 et 2, Redia-PREV2) qui a donné lieu à plusieurs publications dans des revues scientifiques. Axé sur la recherche de cohérence et de qualité dans le cadre des soins du patient, il a participé activement à l'élaboration de réseaux de soins pour les patients présentant un risque cardio-vasculaire (Ile de la Réunion, Maurice, Burundi) en articulant les actions menées à l'hôpital, par les médecins en ville en construisant une approche continue de l'éducation. Membre du Conseil de l'ALFEDIAM et membre de l'ANCRED (Association des réseaux diabète en France), il est impliqué dans la structuration des réseaux de soins et dans la recherche d'une approche d'intégration globale du patient atteint d'une maladie chronique.

Pr Serge Halimi: Professeur Halimi est le chef du département d'endocrinologie à l'Hôpital Universitaire de Grenoble. Ce service accueille des patients de toutes les formes de diabète pour réaliser les examens à des fins diagnostiques ou thérapeutiques. Il prend en charge l'initiation ou l'ajustement de leur traitement et les soins. En parallèle, le Pr. Halimi a dirigé de nombreuses revues médicales, y compris «médecine des maladies métaboliques", a présidé la Société francophone du diabète (SFD) et vient de publier le livre blanc sur le diabète en France.

V - BIBLIOGRAPHIE

[1] Wild S, Roglic G, Green A, Sicree R, King H. Global prevalence of diabetes estimates for the year 2000 and projections for 2030. Diabetes Care 2004 ; 27 : 1047-53.
[2] WHO. Preventing chronic diseases : a vital investment. Geneva : World Health Organization, 2005.
[3] Maire B, Delpeuch F. La transition nutritionnelle, l'alimentation et les villes dans les pays en développement. Santé 2001 ; 11:23-30.
[4] Maire B, Lioret S, Gartner A, Delpeuch F. Transition nutritionnelle et maladies chroniques non transmissibles liées à l'alimentation dans les PVD. Santé 2002; 12:45-55.
[5] Assal JP, Jacquemet S, Morel Y. The added value of therapy in diabetes: the education of patients for self-management of their disease. *Metabolism* 199; 1(Suppl 1): 61- 4.
[6] Peyrot M, Rubin RR, Lauritzen T, et al; on behalf of the International DAWN Advisory Panel. Psychosocial problems and barriers to improved diabetes management: results of the Cross-National Diabetes Attitudes, Wishes and Needs (DAWN) Study. Diabet Med 005; 10: 139-85.
[7] Mulcahy K, Maryniuk M, Peeples M, et al. Diabetes self-management education core outcomes measures. Diabetes Educ 003; 5: 68-0, 3-84, 8-8 passim.
[8]Balcou-Debussche M, editor. L'éducation des malades chroniques. Une approche ethnosociologique. Paris: Éditions des archives contemporaines; 2006.
[9]Allen N. The History of Diabetes Nursing, 1914-1936. *The Diabetes Educator 2*003; 9: 96-89.
[11] Diabetes Control and Complications Trial/Epidemiology of Diabetes Interventions and Complications Study Research Group, Jacobson AM, Musen G, Ryan CM, Silvers N, Cleary P, Waberski B, Burwood A, Weinger K, Bayless M, Dahms W, Harth J. Long-term effect of diabetes and its treatment on cognitive function. N Engl J Med. 2007 May 3;356(18):1842-52.
[12] Nicollerat JA.Implications of the United Kingdom Prospective Diabetes Study (UKPDS) results on patient management. Diabetes Educ. 2000 Nov-Dec;26 Suppl:8-10.
[13]Dunning P. The diabetes educator: evolution of a nurse specialist. *Practical Diabetes* 199; 6: 220-2.
[14]Balcou-Debussche M, Debussche X. Type 2 diabetes patient education in Reunion Island: Perceptions and needs of professionals in advance of the initiation of a primary care management network. Diabetes&Metab. (2008), doi:10.1016/j.diabet.2008.03.002

[15]Balcou-Debussche M., Debussche X. Hospitalisation for type 2 diabetes: The effects of the suspension of reality on patients' subsequent management of their condition. Qualitative Health Research, 2009, *19*,1100-1115.
[16] Beran, D. Besançon. S. Report of the International Insulin Foundation on the assessment protocol for insulin access in Mali. Bamako: International Insulin Foundation, 2004.
[17] Kyrouz EM, Humphreys K, Loomis C. A review of research on the effectiveness of self-help mutual aid groups. *J Am Geriatr Soc* 199; : 14-50.
[18] Norris SL, Chowdhury FM, Van Le K, et al. Effectiveness of community health workers in the care of persons with diabetes. *Diabetic Med* 006; 5: 544-56.
[19] Promising approaches to diabetes self-management: lessons from the diabetes initiative of the Robert Wood Johnson Foundation. *The Diabetes Educator* 00; 6 (Special Issue): S1-S4.
[20] Balcou-Debussche M. Une approche ethnosociologique de l'éducation thérapeutique : les nids d'apprentissage dans le diabète de type 2 . In : *L'éducation thérapeutique en France : pratiques, modèles, évaluations*. Bury J., Foucaud J., Eymard C., Balcou-Debussche M eds, Paris : INPES (à paraître).
[21] Debussche X., Balcou-Debussche M., Cardiovascular risk in diabetes : group education focusing on understanding of key concepts and interactions with social context, 6th IDF-WPR congress, Bangkok, 2005
22] Balcou-Debussche M., Debussche X., Implementation of patient-centred group education in primary care aiming at knowledge appropriation in type 2 diabetes. Therapeutic Patient Education, Florence, Italy, 2006, 27-30th april
[23]Glauber HS, Brown JB. Use of HMO pharmacy databases to study the quality of care and resource use in diabetes mellitus. Diabetes Care. 1992;15:870-876.
[24] Brown JB, Nichols GA, Glauber HS. Case-control study of 10 years of comprehensive diabetes care. West J Med. 2000;172:85-90.
[25]Brown JB, Beck A, Boles M, Barrett P. Practical methods to increase used of advance medical directives: A randomized controlled trial. Journal of General Internal Medicine. 1999;14:21-26.

VI – FORMULAIRE D'INFORMATION

<u>Formulaire d'information</u>

Cette étude a pour objectif de mesurer l'impact biologique et anthropométrique d'une intensification de l'éducation de patients diabétiques de type 2.

Je suis informé à travers ce document et la lecture qu'il m'en est fait que :

- le diabète (ou maladie du sucre) est une maladie chronique, dont les principaux symptômes sont une fatigue excessive, une envie d'uriner régulière, une perte de poids subite, une envie de boire et de manger excessive. La prise en charge optimale de cette maladie associe de l'activité physique régulière, un régime alimentaire adapté et la prise de médicament comme des antidiabétiques oraux ou de l'insuline.
- l'hypertension est également une maladie chronique souvent due à un régime alimentaire riche en sels. Les symptômes sont des maux de tête, des vertiges, etc… Une prise en charge optimale associe un régime alimentaire moins riche en sels, une perte de poids en cas d'obésité et une prise de médicaments antihypertenseurs.
- qu'une piqûre au bout du doigt (pouvant produire une très légère douleur de quelques secondes) sera réalisée afin de prélever quelques gouttes de sang pour mesurer la glycémie à jeun et l'hémoglobine glyquée
- qu'un brassard sera gonflé pendant quelques secondes sur le haut de mon bras afin de mesurer la tension artérielle, cela ne provoquant aucune douleur,
- que les mesures tour de taille et tour de hanche seront réalisées par une femme pour les femmes et par un homme pour les hommes.
- qu'il existe un médecin au sein de l'équipe du Csréf de la commune 1 du district de Bamako à qui je peux m'adresser en cas de problèmes de santé.

- que cette étude pourra comporter le risque de découvrir une complication de mon diabète inconnue de moi-même. Mon bénéfice étant qu'en suivant le protocole d'éducation intensif je pourrai agir préventivement si un risque de développer une complication du diabète apparaît ou, avoir accès à une prise en charge optimale si un problème de complication du diabète est diagnostiqué.

VI – FORMULAIRE DE CONSENTEMENT INDIVIDUEL

Formulaire de consentement libre et éclairé Fiche **n°** : ……

Mon nom est ………………………………………………………………. et je reconnais avoir été informé, dans la langue locale :

- des objectifs de l'étude intitulée «Essai comparatif randomisé de l'apport d'une intervention éducative structurée par des pairs sur l'amélioration de l'HbA1c chez des patients diabétiques de type 2 dans la région de Sikasso au Mali», réalisée par l'unité d'endocrinologie et de diabétologie du CHU du point G et de l'ONG Santé Diabète Mali (SDM).

- des bénéfices et des risques de cette étude

- que le responsable de l'étude est le Pr Assa Sidibé endocrinologue travaillant au CHU du point G (téléphone 66722179)

- que cette étude a été soumise pour validation au comité d'éthique de la faculté de médecine. Celui-ci peut être contacté via le secrétaire principal Pr Idrissa A. Cisse (76132011) ou le président Pr Mamadou M. Keita au numéro (66722022)

- que les résultats de cette étude seront gardés confidentielles.

- que les mesures biologiques seront réalisées gratuitement et qu'il n'y aura pas de compensation financière d'aucune sorte.

En connaissance de cause, j'accepte de participer à l'étude et donc de fournir toutes les informations nécessaires au formulaire d'enquête et permettre à l'équipe responsable de l'enquête de mesurer mon poids, ma taille, mon tour de taille/tour de hanche, ma glycémie, mon hémoglobine glyquée ainsi que ma pression artérielle.

Fait à Bamako Le 17/06/2011

Signature (ou empreinte) Le responsable de l'étude

VII - BUDGET

Budget Global				
**RUBRIQUES (dépenses)**	**Quantité**	**Prix unitaire**	**Total Fcfa**	**Total Euros**
**A - Coûts directs**				
**1 - Phase préparatoire**				
Validation étude prospective par comité éthique				
Frais passage comité éthique	1	300 000	300 000	457
**Sous Total 1**			**300 000**	457
**2- Développement et mise en œuvre du suivi de cohorte prospectif**				
Mise en place (recrutement de 212 patients)				
Test biologique mesure de l'Hba1c	250	3 000	750 000	1 143
Défraiement des patients pour leur déplacement vers les consultations	250	2 000	500 000	762
Indemnités infirmier prélevement	1	30 000	30 000	46
Médecins et Formation des laborantins pour la mesure de l'HBA1C	1	250 000	250 000	381
Visites de suivi à T=3 mois , T= 6 mois et T= 12 mois de cohorte pendant 27 mois				
Visite T=3 mois				
Test biologique mesure de l'Hba1c	150	3 000	450 000	
Mesure de la glycémie à jeun	150	354	53 100	

Défraiement des patients pour leur déplacement vers les consultations	150	2 000	300 000	
Indemnités infirmier prélevement	1	30 000	30 000	
Indemnités consultation médicale	150	1 000	150 000	
Visite T=6 mois				
Test biologique mesure de l'Hba1c	150	3 000	450 000	
Mesure de la glycémie à jeun	150	354	53 100	
Défraiement des patients pour leur déplacement vers les consultations	150	2 000	300 000	
Indemnités infirmier prélevement	150	30 000	4 500 000	
Indemnités consultation médicale	150	1 000	150 000	
Visite T=12 mois				
Test biologique mesure de l'Hba1c	150	3 000	450 000	
Mesure de la glycémie à jeun	150	354	53 100	
Défraiement des patients pour leur déplacement vers les consultations	108	2 000	216 000	
Indemnités infirmier prélevement	1	30 000	30 000	
Indemnités consultation médicale	150	1 000	150 000	
**Sous Total 2**			**8 865 300**	
**3- Matériel**				
Ordinateur portable + imprimante bureau + scanner suivi étude	1	600 000	600 000	
Balance Séca	2	46 000	92 000	
Toise	2	55 000	110 000	
Mètre Ruban	10	100	1 000	
Glucomètre Accu chek active	2	27 500	55 000	
Tensiomètre spengler	2	49 000	98 000	
Petit matériel kit de prélévement	4	75 000	300 000	
Papéterie reproduction questionnaires, piles glucometres etc…	12	35 500	426 000	
**Sous total 3**			**1 682 000**	
**4- Frais de suivi de l'étude**				
Téléphone pour le suivi de l'étude	12	20 000	240 000	366
Téléphone convocation pour les analyses	4	100 000	400 000	610
Essence déplacement suivi processus	12	16 000	192 000	293
**Sous total 5**			**832 000**	**1 268**
**6- Personnel**				
Chercheur principal Pr Sidibé (50% full time)	12	300 000	3 600 000	

Chercheur secondaire / épidémiologiste Dr Nientao et épidémio	12	250 000	3 000 000	
Participation salaire coordinateur sur la zone	12	100 000	1 200 000	
**Sous total 6**			**7 800 000**	
**TOTAL COUTS ACTIVITES**			**19 479 300**	

Annexe 1 : CV :

NAME	POSITION TITLE
Assa Sidibe Traore	Pr in Endocrinology<br>Head service of endocrinology unit in national hospital Point G

EDUCATION (Begin with baccalaureate or other initial professional education, and include postdoctoral training.)

INSTITUTION AND LOCATION	DEGREE	YEAR CONFERRED	FIELD OF STUD
Faculty of Medicine Marseille France	Faculty medecine	1978	Medicine
Faculty of Medicine Marseille France	Diploma Medicine Doctor	1980	Medicine
Faculty of Medicine Marseille France	Certification forensic	1983	Medicine
Faculty of Medicine Marseille France	CES endocrinology	1985	Endocrinolog and diabetolog
Faculty of Medicine of Bamako Mali	Professor Agregation	2007	Endocrinology and diabetology

RESEARCH AND PROFESSIONAL EXPERIENCE:

Titles and hospital functions:

1971-1978: External hospitals in Marseille

1979-1980: Training in the service of Anesthesia and Resuscitation of Professor FRANCOIS, hospital Timone in Marseille (France), and in the clinic Endocrinology Professor Jean WAVE, Timone hospital in Marseille (France)
1980-1985: Training in specialty Endocrinology, Professor J.L. CODACCIONI, hospital design, Marseille (France)
Juin1986-juillet 1991: Physician assistant in the department of neurology Professor Moussa Traore at the National Hospital Point G, Bamako (Mali)
September 1987-mars1988: Head of Service Acting Service Neurological Hospital Point G, Bamako (Mali)
12 juillet1991: Physician assistant in the department of Medicine AB Professor Ali Nouhoum Diallo, Hospital Point G, Bamako (Mali)
February 1995-aout 1995: Doctor committed partner in the university hospital in Angers (CHUR), France, in the service of Rheumatology Professor Maurice AUDRAN
1998: head of clinical assistant in the department of Internal Medicine by Professor Hamar A TRAORE, Hospital Point G, Bamako (Mali)
July 2001: Lecturer (CAMES), Faculty of Medicine and Dentistry Pharmacy (FMPOS), Bamako (Mali)
2004: Head of Internal Medicine Acting 30/04/04 to 30/05/04, Hospital Point G
2006: Head of Internal Medicine Acting 09/01/06 to 13/01/06
November 2006: Pr in endocrinology and metabolic diseases (CAMES)

Titles and university functions:

1991 (July): Assistant Endocrinology at the National School of Medicine and Pharmacy in Mali (ENMP)
1995 (February to August) Chief of Associated Universities clinic in Rheumatology, Rheumatology Service Professor M. AUDRAN, CHUR Angers (France)
1998 (January): Head of Clinical Endocrinology at the Faculty of Medicine and Pharmacy and dentistry (FMPOS) MALI on competition: Order No 98/MESSRS-MSPAS.
2001 (July): Teacher assistant (CAMES) endocrinology at the FMPOS: CTS from 16 to 23 July in Ouagadougou (Burkina Faso).
2006 (November): Maitre conference agregé endocrinology and metabolic diseases (CAMES)

Selection of publications

- Dembele M, Sidibe A.T. Association HTA – Diabète sucré dans le service de médecine interne de l'hopital du point G - Bamako. Médecine d'Afrique Noire : 2000, 47 (6) Besançon. S. Diarra. A. 2004
- Sidibé.A.Traoré.H.A.Liman-Ali.I.T.Dembélé.M.Traoré.AK.Cissé.I.Diallo.D.Keita.M.M, Le diabète juvénile au Mali. Revue Française d'Endocrinologie Clinique, 1999. 40(6): p. 513-521.
- Masson. C. Sidibé. A. Lombalgie chronique : de l'inorganicité supposée à l'organicité prouvée : A propos d'une illustration de syndrome Sapho = Chronic lombalgia. Est médecine. 1996, no43, pp. 10-11
- AT Sidibé, M Dembélé, A Cisse, F Alwata, MY Ahmedou Oulda, T Coulibaly, AK Traore, HA Traore. A Toure and D. Diallo. Diabetic hand infections in hospital practice in Bamako, Mali. Diabetes & Metabolism Volume 32, Issue 1, February 2006, Page 89
- AT Sidibé. Hyperthyroïdie chez l'enfant. Expérience d"un service de médecine interne au Mali Annales d"endocrinologie. Volume : 68 Numéro : 2-3, 2007, p. : 177 -180
- AT SIDIBE, I. CISSE, M. DEMBELE, AI BOCOUM, S. AG ABOUBACRINE, S. DIARRA, AK TRAORE, HA TRAORE. Urgences en diabétologie. Place de l'hyperosmolarité. Médecine d'Afrique Noire - n° 5210 - Octobre 2005 - pages 552-554
- DIALLO D. A. BABY M. DEMBELE M. KEITA A. SIDIBE A. T. CISSE I. Ah. DIOP C. T. MAÏGA I. I. TRAORE A. K. TRAORE H. A. Fréquence, facteurs de risque et valeur pronostique de l'anémie associée au VIH/sida chez l'adulte au Mali. = Frequency, risk factors, prognosis value of hiv associated anaemia in adult in Mali. Bulletin de la Société de pathologie exotique. 2003, vol. 96, no2, pp. 123-127

Research Support..

- A 3-years project was completed and evaluated to measure the impact of the use of peer educators from the community in the prevention of diabetes in Mali.

Founded by Sanofi Aventis, the World Diabetes foundation and the European Union
- A 4-year project has been completed and evaluated to measure the impact on patients of the introduction of a decentralization of management for diabetics through the creation of "diabetes unit" in Mali
Founded by Sanofi Aventis, the World Diabetes foundation, the European Union, the Swiss cooperation and the French ministry
- A 2-years project to map the GI of mean Malian food and assess the impact on Hba1c of diabetic patients with new dietary recommendations resulting from this research.
Founded by the Wolrd Diabetes Foundation
- A 1-year project on the evaluation of the hypoglycaemic effect of a traditional plant (Scleroccaria Birrea). Founded by the Swiss cooperation
- A 2-years project to assess the economic impact of diabetes mellitus in Africa (project in 6 countries on the continent)
Founded by the International Diabetes Federation

NAME<br>Besançon Stéphane	POSITION TITLE<br>Biologist, nutritionist and physiopathologist of the nutrition (specialized for developing countries) (MSc)<br>Programme Director NGO Santé Diabète Mali

EDUCATION (Begin with baccalaureate or other initial professional education, and include postdoctoral training.)

INSTITUTION AND LOCATION	DEGREE	YEAR CONFERRED	FIELD OF STUDY
University of Sciences of Grenoble (France)	Deug en science	1995-1997	Biology
University of Sciences of Grenoble (France)	first degree in science	1997-1998	Cellular biology and Physiology
University of Sciences of Grenoble (France)	master's degree in science	1999-2000	Physiology and physiopathology
University of Sciences of Montpellier (France)	master's degree in science	2000	Biology, physiology and nutrition specialized for developing countries
Faculty of medicine of Rennes (France)	Specialized university degree	2000-2002	Physiopathology of nutrition with specialization on diabetes

RESEARCH AND PROFESSIONAL EXPERIENCE:

Professional experience

2000 Realization of a research project, with the International Agricultural Research centre for Development, entitled « Intérêt de la consommation d'une céréale le fonio (Digitaria exilis) chez les patients diabétiques maliens ». This research project was carried out in the frame of the project "amélioration des technologies post-récolte du fonio" under the aegis of the IGC (International Grains Group on) and financed by the CFC (Common Fund for Commodities) of the united nations.
2000-02 Program Coordinator of a non-profit association in France.
Responsible for the development of new projects of the association, relations with donors and media.
Management of an annual budget of 250 000 Euros.

2003 Creator of the French Non-Governmental Organization (NGO) Santé Diabète Mali (SDM)
Since 2003: Program Director of the NGO Santé Diabète Mali (SDM) based in Bamako (Mali)
Installation of the delegation in Mali. Signature of the framework agreements and other agreements with the Malian government. In charge of the administrative and financial management (8 full-time employees and 45 temporary employee;) of the delegation (annual budget 750 000 Euros).
Responsible for the design, writing and implementation of the projects and relationships with donors and media.
Presentation of the NGO work in international congresses.

Other experiences
2004 - 2005: Member of the expert group for the drafting of the national policy of prevention and fight against diabetes in Mali
2006: Organization on 29 and 30 June 2006 in Bamako, of the workshop of the WHO and the International Diabetes Federation Africa Region on national strategies for the prevention and fight against diabetes in Africa. Workshop that gathered 75 participants from 17 countries.
2006 - 2007: Member of the expert group for the development of algorithms and training modules included in the national policy of prevention and care of diabetes mellitus in Mali
2006: Participation to the adaptation of the French versions of the guidelines developed by the International Diabetes Federation Africa Region:
- Training Manual for educators on diabetes in Sub Saharan Africa (International Diabetes Federation Africa Region)
- Guide of type 2 diabetes management in Sub Saharan Africa (International Diabetes Federation Africa Region)
2006 : Participation in the working group on the establishment of the inquiry Step Wise in Mali
2007: Realization of an expert mission for the Health Ministry of Guinea Conakry on the establishment of prevention and care policy of diabetes in Guinea Conakry
2007: : Realization of an expert mission in Burkina Faso on the possibility of establishing a decentralized care for diabetes in Burkina Faso
2008: Realization of an expert mission in Madagascar on the possibility of establishing a decentralized care for diabetes in Madagascar
2008 : Participation to the expert group for the preparation and drafting of a food guide for Mali

Projects implemented:
1 - 2000 : Realization of a research project, with the International Agricultural Research centre for Development, entitled « Intérêt de la consommation d'une céréale le fonio (Digitaria exilis) chez les patients diabétiques maliens ».
Research management, data processing and publication of results
Funding: UN
2 – 2004 : Realization of the Rapid Assessment protocol for insulin in Mali.
Co direction of research and joint publication of results
Financing: Diabetes Uk foundation
3 – 2003 – 2005: Project to improve the nutritional care of diabetic patients in Mali. This project consisted of three phases: 3 food surveys of diabetic patients (method: 24 hour recall), the measurement of the Glycaemia Index of the major cereals and sauces eaten in Mali (method of the WHO) and analysis of the biochemical composition of these foods.
Creation of the project, obtaining funding, direction of research, data processing and publication of results.
Supervision of the thesis which was presented on this topic at the faculty of medicine, pharmacy and odonto-stomatology of Mali
Financing: World Diabetes Foundation
4 – 2003 – 2005: establishment of a pilot methodology for primary prevention of diabetes mellitus using peer educators from the community in the commune 1 and 2 of the of the district of Bamako.
Creation of the project, obtaining funding, project management, development of methodology and tools and project implementation.
Financing: World Diabetes Foundation, Switzerland cooperation for development and the French Ministry of Foreign Affairs
5 – 2006 : project of clinical monitoring of diabetic patients in Mali to test the impact of new dietary recommendations coming from the researches preceding that work.
Creation of the project, obtaining funding, direction of research and data processing
Financing: World Diabetes Foundation and the French Ministry of Foreign Affairs
6 – 2006 – 2007 : intensification of the pilot project for primary prevention by peer educators in the commune 1 of the of the district of Bamako and pilot expansion test in the region of Sikasso
Creation of the project, obtaining funding, project management, development of methodology and tools and project implementation.
Financing: World Diabetes Foundation, Switzerland cooperation for development and the French Ministry of Foreign Affairs
7 – 2006 - 2007 : research project on the hypoglycaemic effect of a traditional plant: Sclerocarya Birrea This project included 2 parts: an anthropological investigation and a research phase 1 of the patients Creation of the project, obtaining funding, direction of research, data processing and publication of results. Supervision of the thesis which was presented on this topic at the faculty of medicine, pharmacy and odonto-stomatologie of Mali
Funding: Switzerland cooperation for development

8 – 2005 – 2007 : Pilot training project of caregivers and care restructuring for the care of diabetes mellitus in 3 regions of Mali (Bamako, Sikasso and Timbuktu) and the circle of Douentza
This project consisted of four components: the nursing staff training, access to medicines, access to consultation equipment and analysis equipment, patient education.
Creation of the project, obtaining funding, project management, development of methodology and tools and project implementation.
Financing: Sanofi Aventis, World Diabetes Foundation, Switzerland cooperation for development, the General Council of Essonne and region Rhône alpes.
9 – 2007 : prevention pilot project in primary schools of the district of Bamako.
Creation of the project, obtaining financing, development of methodology and tools, project management.
Financing: Sanofi Aventis and Switzerland cooperation for development.
10 – 2006 – 2007 : Project to support the creation or boosting of diabetic patients Association in areas in which decentralized consultations are operational
Creation of the project, obtaining funding, project management, development of methodology and tools and project implementation.
Funding: Switzerland cooperation for development and World Diabetes Foundation.
11 – 2007 : Creation of a 10 minutes film " Le diabète nouvel enjeu de santé publique pour l’Afrique ."
Elaboration of the script and drafting the comment
Financing: European Union, the General Council of Essonne and the General Council of Isere

Projects under way:
1 - 2008 – 2009 : Research Project "evaluate the impact of diabetes in Africa". This project is conducted in 6 countries of the African continent (South Africa, Cameroon, Kenya, Tanzania, Guinea Conakry and Mali).
Lead researcher for the implementation of the project in Mali
Financing: International Diabetes Federation (IDF)
2 - 2008 – 2009: Pilot Project of dissemination of the education and care of type 2 diabetes guidelines for Africa made by the International Diabetes Federation Africa. This project is conducted in 10 countries of the African continent.
Director of the implementation in Mali
Financing: World Diabetes Foundation
3 - 2008 – 2009 : Project to improve the care of diabetic children in Mali.
Creation of the project, obtaining funding, project management
Funding: International Diabetes Federation (IDF)
4 – 2008–2009 : project of assessment of the primary prevention methodology of risk factors for chronic non-communicable diseases by peer educators in the commune 1 of the district of Bamako, in the town of Sikasso and the town of Timbuktu (validation the methodology for a dissemination in different countries of the continent). Creation of the project, obtaining funding, project management, data processing and publication of results
Financing: European Union, World Diabetes Foundation, Switzerland cooperation for development, Rhone Alps, French mutuality, the General Council of Essonne and the French Ministry of Foreign Affairs
5 – 2008–2009 : project of assessment of the primary prevention methodology of risk factors for chronic non-communicable diseases in schools of the district of Bamako and the town of Sikasso (validation of the methodology for a dissemination in different countries continent)
Creation of the project, obtaining funding, project management, data processing and publication of results
Financing: European Union, Sanofi Aventis, Switzerland cooperation for development, Rhone Alps, French mutuality, the General Council of Essonne and the French Ministry of Foreign Affairs
6– 2008– 2009: project of assessment of the methodology of care restructuring for the care of diabetes mellitus in 6 of the 8 regions of Mali (District of Bamako, region of Sikasso, Timbuktu, Mopti, Kayes and Segou). (Validation of methodology for a dissemination in different countries of the continent)
This project consisted of four components: the nursing staff training, access to medicines, access to consultation equipment and analysis equipment, patient education.
Creation of the project, obtaining funding, project management, data processing and publication of results.
Financing: European Union, World Diabetes Foundation, Switzerland cooperation for development, Rhone Alps, French mutuality, the General Council of Essonne and the French Ministry of Foreign Affairs
7 – 2008 – 2009 : Project to improve the prevention and care of diabetic foot using the Step by Step methodology developed in Tanzania
Creation of the project, obtaining funding, Adaptation of tools and project management.
Financing: European Union, World Diabetes Foundation, Switzerland cooperation for development, Rhone Alps, French mutuality, the General Council of Essonne and the French Ministry of Foreign Affairs
8– 2008–2009 : project of assessment of the primary prevention methodology of risk factors for chronic non-communicable diseases through media (cartoons, messages on radios, etc…)
Creation of the project, obtaining financing, development of tools and project management.
Financing: World Diabetes Foundation, French mutuality, Switzerland cooperation for development and the French Ministry of Foreign Affairs

Publication (scientific, international report and book)
1 – Besançon. S. *Study of the influence of the consumption of fonio (Digitaria Exilis) in the treatment of diabetes mellitus in Mali*. Centre International en recherche agronomique pour le développement (CIRAD).100 pages, 2000.
2 - Beran, D. Besançon. S. *Report of the International Insulin Foundation on the assessment protocol for insulin access in Mali*. International Insulin Foundation. 40 pages, 2004.
3 - Besançon. S. Beran. D. *Diabète en Afrique : un nouvel enjeu de santé publique*. Equilibre. N°254, novembre - décembre 2006.
5 – Besançon. S. *Le rôle d'une association de patients : l'Association Malienne de Lutte contre le Diabète (AMLD)*. Diabetes voice. Vol 51, n°3, septembre 2006.
7 - Besançon. S. Sidibe. A. Beran. D. *Le diabète un nouvel enjeu de santé publique pour les pays en voie de développement : le cas du Mali*. Médecine des maladies métaboliques. Vol 1, n°1, mars 2007.
6 - Besançon S. 2004. *Analysis of food consumption of diabetic patients of the Malian city of Bamako, Sikasso and Timbuktu by the 24 hours recall method*. International report of NGO Santé Diabète Mali. 120 pages, décembre 2006.
7 - Besançon. S. Nientao. I. *Study of the glycemic index of main cereals consumed in Mali*. International report of NGO Santé Diabète Mali. 50 pages, décembre 2006.
8 - Besançon. S. Nientao. I. *Study of the impact of major sauces consumed in Mali on post prandial on blood sugar*. International report of NGO Santé Diabète Mali. 40 pages, décembre 2006.
9 - Besançon. S. Nientao . I. *Impact of new dietary recommendations on the glycemic management of Malian diabetics patients*. International report of NGO Santé Diabète Mali. 45 pages, mars 2007.
10 – Besançon. S. *Diabetes and HIV in west Africa region*. Newsletter of United Nations. November 2007
11 – Besançon. S. *Development of information, education by communication (IEC) methodology, using peer educators from community, for carrying out primary prevention on diabetes risk factors*. International report of NGO Santé Diabète Mali. 60 pages, décembre 2007.
12 – Besançon. S. *Development of information, education by communication (IEC) methodology for carrying out primary prevention on diabetes risk factors with children in schools*. International report of NGO Santé Diabète Mali. 30 pages, décembre 2007.
13 – Besançon. S. Nientao. I. *Developing a model of decentralization of care for the care of diabetes in Mali*. International report of NGO Santé Diabète Mali. 90 pages, décembre 2007.
14 – Besançon. S. Nientao. I. Sanogo. S. *Study of the hypoglycaemic effect of a traditional plant: Sclerocarya Birrea*. International report of NGO Santé Diabète Mali. 50 pages, décembre 2007.

Oral Communications
1 – "*How to develop a programme to improve prevention and management of diabetes mellitus: the example of Mali"*.
International workshop on the emergence of diabetes mellitus in developing countries
25 to 28 April, 2006, Manilla (Philippines)
2 – *"Management of diabetes mellitus in Mali: a global strategy"*
REMED International workshop: How to better engage in the fight against chronic diseases in developing countries
13 novembre 2006, Paris (France)
3 – *"Nutritional management of diabetes in Africa: the example of Mali"*
19th World Diabetes Congress, december 3rd to 9th ,2006, Cape Town, South Africa,
4 – *"Nutritional management of diabetes in Africa"*
Global African Diabetes Summit, n ovember 30th, 2007, Nairobie, Kenya.
5 - "*How and to what extent can coalitions of NGO's influence the emergence of new rights and norms under international law*"
Discussing in the session: NGOs as regulators in global exchanges Example of access to medication and other treatments.
Handicap International Annual Conference, February 13th, 2008, Lyon, France.
6 - "Prevention and management of diabetic foot in Mali"
International workshop on implementation of prevention and management of diabetes mellitus programmes in developing countries, february 13th, 2008, Lyon, France.

NIENTAO Ibrahim Antoine
30 ans
Malien
Hamdallaye ACI 2000
Rue 320, Porte 416 Bamako-Mali
Tel (+223) 6729949
ibnientao@yahoo.fr

Diabétologue Centre national de Lutte contre le Diabète

FORMATION INITIALE

**2007-2008**	**DU de diabétologie à l'université d'Oran en Algérie**
**Octobre 2005**	Soutenance de la **thèse de médecine sur l'index glycémique des principales céréales consommées au Mali** à l'université de Bamako (ancienne université du Mali) **- Mention très honorable,** proposition de prix de thèse et échange avec les autres facultés.
**2001- 2005**	Interne dans le service de Diabétologie et de médecine interne du centre hospitalier universitaire Gabriel Touré.

**1998-2001**	Stages pratiques et obligatoires en service de médecine interne, de pédiatrie, de chirurgie générale, des urgences chirurgicales et de la réanimation dans les hôpitaux nationaux du Mali (Gabriel Touré et du Piont-G).
**1997-2003**	Faculté de médecine de Pharmacie et d'odonto-stomatologie de l'université du Mali (FMPOS)
**1997**	Baccalauréat Malien série science biologique – Mention Assez Bien

EXPERIENCE PROFESSIONNELLE

**Depuis mars 2006**	**Médecin consultant au Centre National de Lutte Contre le Diabète du Mali**<br>**Chef de projet structuration des soins et formateur** à l'ONG Santé Diabète Mali<br>Réalisation des études de l'influence de la consommation des principales céréales et sauces consommées au Mali sur la réponse post prandiale de la glycémie<br>Réalisation de l'étude « Impact de l'éducation thérapeutique et nutritionnelle sur l'équilibre des patients diabétiques »<br>Formateur des médecins et paramédicaux (infirmiers, sages-femmes, laborantins…) des 6 communes du district de Bamako (capitale du Mali), Sikasso (3ème Région du Mali), Tombouctou (6ème région du Mali), Kayes (1ere région du Mali), Ségou (4ème région du Mali) et Mopti (5ème région du Mali) pour l'amélioration de la prise en charge du diabète sucré au Mali.
**Sept. 2006**	Conférencier, représentant de l'ONG Santé Diabète Mali à Mindelo (CAP-VERT) au 10ème forum de nutrition de la CEDEAO. Thème : *Nutrition et maladies chroniques dans le cadre de la double charge de morbidité de la malnutrition.*
**Juillet 2006**	Invité au séminaire FID Afrique/ OMS Afro de Bamako sur les politiques nationales de prévention et de lutte contre le diabète en Afrique, zone francophone.
**Depuis janv. 2006**	Médecin titulaire au Centre de lutte contre le diabète de Bamako.
**Juin 2004-Sept 2005**	Consultations médicales au Centre de lutte contre le diabète de Bamako
**Oct. 2004 & nov. 2005**	Formateur des pairs éducateurs et du personnel soignant (médecins, infirmiers et sages-femmes) de la commune 2 du district de Bamako sur le diabète (formations initiale et recyclage)

AUTRES DOMAINES

Langues

- Bambara : langue maternelle

- Français : parlé, écrit
- Anglais : passable
- Allemand : notions

Informatique Bureautique (Word, Excel, PowerPoint)
Recherches Internet

DEBUSSCHE Xavier, André, François
Né le 15/04/1958. Nationalité française

Docteur en Médecine (Octobre 1986)
Endocrinologie et Maladies métaboliques

Endocrino-Diabétologie, Pôle des Pathologies Chroniques et Maladies Métaboliques -
CHR de la Réunion - 97405 Saint Denis Cedex - La Réunion, France.
Tel : (262)262905610 ; Fax : (262)262907763 **;** Courriel : xavier.debussche@chr-reunion.fr

Titres et fonctions

Responsable du Pôle Pathologies Chroniques et Maladies Métaboliques, CHR de la Réunion (2005-2008)

Coordinateur Médical de la Recherche, Délégation à la Recherche Clinique et l'Innovation, CHR de la Réunion (2008-présent)

Chef de Service (2001-présent) : Diabétologie- Endocrinologie, CH Félix Guyon, Réunion

Praticien Hospitalier (1997-présent : CHR Réunion ; 1991-1996 : CHRU Amiens)

Membre du bureau de la DIRC SO-OM (Direction Inter Régionale de la Recherche Clinique Sud Ouest Outre-Mer ; 2006-présent)

Coordinateur médical du réseau RéuCARE (Réunion Cœur Artères Rein Education : prise en charge éducative des patients à haut risque cardiovasculaire et rénal), 2003-présent

Promoteur et coordinateur du Diplôme Universitaire d'Education pour la Santé/ Maladies Chroniques, La

Réunion.
Ancien CCA (Paris 7 et Amiens, 1986-1990), Endocrinologie et Maladies Métaboliques
Ancien Interne des Hôpitaux (CHRU Amiens, 1981-1986)

Membre de Sociétés Savantes :
ALFEDIAM-SFD (membre du Conseil d'Administration), EASD (European Association for the Study of Diabetes), ADA (American Diabetes Association), SOPHE (Society for Public Health Education), SFSP (Société Française de Santé Publique), DELF (Diabetes Education study group de Langue Française), SEDNMR (Société d'Endocrinologie Diabète Nutrition Métabolisme de la Réunion).

Activités de recherche
- Diplôme d'Etudes Approfondies en Physiologie et Physio-pathologie de la Nutrition Humaine (Pr Picon, Paris 7), septembre 1987.
- Travaux sur l'acidose lactique au cours du traitement par metformine, Amiens, 1984-1990.
- Travaux sur la souris NOD prédisposée au diabète de type 1, laboratoires de Diabétologie du Pr Assan (Faculté Bichat), d'Immunologie du Pr Boitard (Inserm U81, Hôpital Necker), et d'Histo-pathologie du Pr Potet (Hôpital Bichat). Septembre 1986 à Juillet 1993.
- Mise au point de méthodes de mesure de la résistance à l'insuline, clamp euglycémique hyperinsulinémique, CHRU Amiens, 1990-1992.
- Groupe de Recherche Français sur la détection précoce et la prévention du diabète sucré de type 1, 1991-1994
- Particularités individuelles et culturelles et impact sur les actions de prévention et d'éducation dans le diabète de type 2 ; développement d'une approche en Education en Santé mobilisant les nids d'apprentissage en groupe, La Réunion (Collaboration Sciences Humaines : CURAPP Université d'Amiens, LCF-CNRS Université de la Réunion, PAEDI Université de Clermont Ferrand), 2000-présent.
- Membre élu du CORI (Conseil d'Orientation Recherche INSERM) - Réunion, 2000-2003.
- Réseau de Recherche Régional INSERM en Santé Publique de la Réunion, 2001-2004
- Co-investigateur : REDIA-1 (1999-2001), REDIA-2 Cohorte (2006-2009) : épidémiologie du diabète à la Réunion
- Co-investigateur, recherche prospective randomisée REDIA Prev 2 (étude d'intervention de prévention des complications du diabète de type 2 à la Réunion) : 2003-2006
- Investigateur associé et membre du comité médico-technique du CIC-EC INSERM (Centre d'Investigation Clinique et d'Epidémiologie Clinique) de la Réunion, 2004-présent.
- Reviewer : Medical Science Monitor, Diabetes & Metabolism, Open Diabetes Journal.

Travaux, communications, et publications

Balcou-Debussche M., <u>Debussche X</u>. Hospitalisation for type 2 diabetes: the effects of the suspension of reality on patients' subsequent management of their condition. Qualitative Health Research, 2009, 19 :1100-1115.
Mourot L, Boussuges A, Campo P, Maunier S, <u>Debussche X</u>, Blanc P. Cardiovascular rehabilitation increase arterial compliance in type 2 diabetic patients with coronary artery disease. Diabetes Res Clin Pract. 2009
Balcou-Debussche M., <u>Debussche X</u>., Type 2 diabetes patient education on Reunion Island: perceptions and needs of professionals at the initiation of a primary care management network, Diabetes & Metabolism, 2008, 34, 375-381.

Debussche X. Le Diabète à la Réunion. Transitions de société et lenteurs d'adaptation. Diabète & Obésité, 2008, 23, 94-99.

Le Moullec N, Fianu A, Debussche X, Le Pommelet C, Boyer MC, Favier F. Gamma Glutamyl Transférase (GGT) et syndrome métabolique à la Réunion- Données issues de la cohorte REDIA 1. ALFEDIAM, Bruxelles, 2008.

Blanc P, Boussuges A, Maunier S, Benyouma A, Chopra S, Debussche X. Comparaison de la capacité physique de patients coronariens obèses et de patients ayant un poids normal en rééducation cardiaque. ALFEDIAM, Bruxelles, 2008.

Debussche X., Apprendre à gérer les dépenses d'activité physique, Livret pour le formateur et l'apprenant.. Editions des Archives Contemporaines (Paris) 2008.

Debussche X, Balcou-Debussche M, Roddier M. A practical and socio-cultural approach in global cardiovascular risk assessment and preventive self-management by patients. 7th International Symposium on Multiple Risk Factors in cardiovascular Disease: prevention and intervention – Health policy. Italie, octobre 2008.

Debussche X, Balcou-Debussche M. A structured TPE programme for cardiovascular prevention in primary care. 2nd therapeutic patient education congress, Budapest, novembre 2008.

Debussche X., Apprendre à maîtriser le Risque Cardio Vasculaire , Livret pour le formateur et l'apprenant. Editions des Archives Contemporaines (Paris) 2007.

Debussche X., Sabattie O. Apprendre à maîtriser l'apport de graisses dans l'alimentation , Livret pour le formateur et l'apprenant. Editions des Archives Contemporaines (Paris) 2007.

Blanc P, Boussuges A, Maunier S, Chopra S, Debussche X. Prevalence of cardiovascular risk factors and physical capacity in cardiac rehabilitation: Is there a difference between diabetic and non diabetic patients? European Association for the Study of Diabetes, 2007.

Debussche X , Balcou-Debussche M. Évaluer l'impact d'une maladie émergente (le Chikungunya) chez des patients atteints d'une maladie chronique et de milieux sociaux différenciés, afin d'améliorer la prévention des risques en santé en milieu tropical. Colloque InVS-CRVOI Chikungunya et autres arboviroses émergentes en milieu tropical, Saint-Pierre, la Réunion. Décembre 2007.

Debussche X, Roddier M, Fianu A, Lemoullec N, Papoz L, Favier F and The Redia Study Group. Health perceptions of diabetic patients in the REDIA study. Diabetes Metabolism, 2006, 32, 50-55.

Balcou-Debussche M., Debussche X., Implementation of patient-centred group education in primary care aiming at knowledge appropriation in type 2 diabetes. Therapeutic Patient Education, Florence, Italy, 2006, 27-30th april.

Favier F, Jaussent I, Lemoullec N, Debussche X, Boyer MC, Schwager JC, Papoz L. Prevalence of type 2 diabetes and central adiposity in La Réunion Island. Diab Res Clin Pract, 2005, 67, 234-242.

Balcou-Debussche M., Debussche X., Le Moullec N., Favier F., Physical activity : plurality of issues constitutive of its implementation in type 2 diabetes, a qualitative study, 6th IDF-WPR congress, Bangkok, 2005

Debussche X., Balcou-Debussche M., Cardiovascular risk in diabetes : group education focusing on understanding of key concepts and interactions with social context, 6th IDF-WPR congress, Bangkok, 2005

Balcou-Debussche M., Debussche X., Le Moullec N., Favier F., Pratiques alimentaires de patients diabétiques ayant bénéficié de séances d'éducation à l'hôpital , ALFEDIAM 2005, Lyon.

Debussche X., Balcou-Debussche M., Une action de formation en éducation du patient centrée sur des situations d'apprentissage en groupe : l'expérience du Diplôme Universitaire de la Réunion. Éducation du Patient et Enjeux de Santé, 2005, 23, 63-68.

Balcou-Debussche M., Debussche X., Cognitive activities of patients and interactive educational situations in an outpatient hospital setting, 18th International Diabetes Federation Congress, Diabetes Metabolism 2003, 29, 4S382.

Balcou-Debussche M., Debussche X., Changes of perceptions and attitudes of health professionals after a 1-yr long course aiming at the implementation of diabetic patient education network, 18th International Diabetes Federation Congress, Diabetologia, 2003, 46 (suppl 2) A95.

Balcou-Debussche M, Debussche X. From transmission to construction of knowledge and skills : a new paradigm for diabetes education and training. Diabetes Res Clin Pract, 2002, 56 (suppl 1), S34-S35.

Debussche X, Roddier M. Behavorial factors of care in type 2 diabetes mellitus: complementarities of quantitative and qualitative methods. Diabetes Res Clin Pract, 2002, 56 (suppl 1), S82-S83.

Roddier M, Debussche X. La prise en compte des particularités individuelles des diabétiques. Étude anthropologique à l'île de la Réunion., Diabète-Education, 2001, 11, 4-7.

Debussche X, Roddier M, Balcou M, Boyer-Doye MC. Anthropological approach of type 2 Diabetes Mellitus as a prerequisite in adaptative strategies of patient's education : the example of Réunion Island. Diabetes Res Clin Pract, 2000, 50 (suppl 1), S27.
Debussche X, Balcou M, Roddier M. Didactic methods as a tool in the training of diabetes educators in Réunion Island. Diabetes Res Clin Pract, 2000, 50 (suppl 1), S27.
Debussche X, Roddier M, Balcou M, Boyer-Doye MC. Insulin therapy in type 2 diabetic subjects : an anthropological study of patient's perceptions in Reunion Island. Diabetes Res Clin Pract, 2000, 50 (suppl 1)
Debussche X, B Lormeau, C Boitard, M Toublanc, R Assan. Course of pancreatic beta cell destruction in prediabetic NOD mice : a histomorphometric evaluation. Diabetes & Metab., 1994, 20, 282-290
Lalau JD, PF Westeel, F Tennenbaum, X Debussche, J Nussberger, B Tribout, P Fardellone, H Favre, A Fournier. Natriuretic and vasoactive hormones and glomerular hyperfiltration in hyperglycaemic type 2 diabetic patients : effect of insulin treatment. Nephron, 1993, 63, 296-302.
Boitard C, J Timsit, R Assan, A Mogenet, X Debussche, E Kalioustian, JR Attali, P. Chanson, L Chatenoud, T Woodworth, JF Bach Phase I/II trial of DAB 486-IL2 fusion toxin in patients with recent onset, type 1 diabetes mellitus.. 53th ADA Annual Meeting, 1993, (Diabetes, 1993, 42, suppl.1, 205A)
Fendri S, X Debussche, H Puy, O Vincent, JM Marcelli, A Dubreuil, JD Lalau. Metformin effects on peripheral sensitivity to insulin in non diabetic obese subjects. Diab. Metab., 1993, 19, 245-249.
Assan R, J Delaby, D Assan, E Larger, X Debussche, M Toublanc. Pentamidine- induced dysglycaemia : experimental model in the rat. Diab. Metab., 1993, 19, 262-272.
Lormeau B, X Debussche, S Gross, H Puy, S Fendri, J Delobel, J Quichaud. Acute hyperinsulinism has no effect on fibrinolysis in lean and obese subjects. 14th IDF Congress, 1991.
Debussche X, H. Hanaire-Broutin, B. Jacotot, A. Fournier. Physiopathologie de l'athérosclérose. *In* Hypertension artérielle, Hermann eds, collection Sciences et Pratiques Médicales, 1992, pp 113-135.
Lalau JD, A Fournier, M Phlipponneau, X Debussche, PF Westeel.Hypertensions endocriniennes et métaboliques. *In* Hypertension artérielle, Hermann eds, collection Sciences et Pratiques Médicales, 1992, pp 291-340.
Debussche X, JD Lalau, B Jacotot, A Fournier. Traitement des dyslipidémies. *In* Hypertension artérielle, Hermann eds, collection Sciences et Pratiques Médicales, 1992, pp 549-566.
Lalau JD, Debussche X, Fournier A, Quichaud J. Management and treatment of lactic acidosis. Journ Annu Diabetol Hotel Dieu. 1990:89-103. Review.
Debussche X, Lalau JD, Quichaud J. Management and treatment of diabetic keto-acidosis. Journ Annu Diabetol Hotel Dieu. 1990:75-88.

BALCOU-DEBUSSCHE Maryvette

Tél : 02 62 30 21 18
Adresse électronique : maryvette.balcou@wanadoo.fr

Cursus Universitaire, diplômes, concours

2007	Classement au concours de recrutement MCF, Université Bobigny-Paris XIII
2006	**Qualification CNU**, section 19 (Sociologie-Démographie)
2006	**Qualification CNU**, section 70 (Sciences de l'Education)
2006	Classement au concours MCF, IUFM de Nice & Université de la Réunion
2002	**Qualification CNU**, section 70 (Sciences de l'Education)
2001	**Doctorat en Sciences de l'Education**, UPJV, Amiens, 2001.<br>Directeur de recherche : Professeur F. Ropé, UPJV, Amiens.<br>Jury composé de : F. Ropé, E. Bautier, N. Lautier, V. Leclercq , J. Simonin.
	Mention obtenue : *Très honorable, avec félicitations du jury, à l'unanimité.*
1997	**D.E.A.** "Savoirs, Individus, Sociétés", Sciences de l'Education, Mention Bien, UPJV, Amiens
1996	**Maîtrise**, Sciences de l'Education, Mention Bien, Université de la Réunion
1993	**Licence**, Sciences de l'Éducation, Mention Bien, Université de la Réunion
1993	3ème au concours Professorat des Ecoles, Rectorat de la Réunion

2008	Autres : Monitorat premier secours & Monitorat SST

Principales expériences professionnelles

2002-2009	**Formatrice en éducation à la santé et chercheur à l'Université-IUFM de la Réunion**<br>Analyses de pratiques professionnelles - Sociologie de l'Education et de la Formation des adultes - Direction de mémoires professionnels - Conseils pédagogiques, analyses– Formation continue des personnels – Conférences
2008-2009	**Experte éducation thérapeutique / Sciences de l'éducation pour l'INPES**<br>=> Co-direction d'un ouvrage à paraître (2009)<br>=> Co-direction d'un ouvrage sur les compétences en ETP (2008)
2006-2009	**Formation de personnels à la problématique de l'éducation en santé, dans les structures communautaires et ONG**<br>⇨ Burundi : projet financé par la World Diabetes Foundation,<br>⇨ Mise en place d'un réseau d'éducation et développement des nids d'apprentissage (3000 patients diabétiques sur 3 ans).<br>⇨ Ile Maurice, (300 patients en 2007).
2009	**Intervention en éducation thérapeutique** auprès des infirmiers en Centre Hospitalier<br>=> Développement des nids d'apprentissage, Paris & province
2005-2009	**Responsable pédagogique du Diplôme Universitaire** « Education et prévention des maladies chroniques », Université de la Réunion
2004-2009	**Formation à l'éducation thérapeutique** dans le réseau de santé Réucare, La Réunion (2000 patients inclus, 400 professionnels de santé membres du réseau) et au Centre Hospitalier Félix Guyon
2006-2007	**Formation à l'éducation thérapeutique** pour les personnels des Maisons du diabète & Maison DOC à Paris<br>=> Développement des nids d'apprentissage, Paris & province
2005-2007	**Responsable du module** « Didactique de l'écrit », Diplôme Universitaire de prévention et de lutte contre l'illettrisme.
2005-2006	**Formation en didactique pour les moniteurs et doctorants**, dans le cadre du C.I.E.S. Aquitaine-Outremer, Université de la Réunion
2000-2002	**Responsable du module de didactique** dans le Diplôme Universitaire « Prise en charge éducative des patients diabétiques de type 2 », Université de la Réunion
1998-2002	**Chargée de cours en sociolinguistique et didactique**, Université de la Réunion. Collaboration avec Michel Roger, MCF, La Sorbonne
1991-1998	**Formatrice en Centre Lecture-Ecriture**, Rectorat de la Réunion<br>Formation initiale et continue des professionnels (Réunion, Zone Océan Indien)
1981-1991	**Enseignante, 1er degré** (Côtes d'Armor et Réunion)

Profil scientifique : Ethnosociologue de la santé & de la formation des adultes

<u>2008-2009 : Membre du Laboratoire PAEDI</u> (Processus d'Action des Enseignants : Déterminants et impacts), IUFM-Université d'Auvergne .

- ⇨ Équipe d'Accueil (4281).
- ⇨ Unité rattachée à l'école doctorale lettres et SHS de l'Université balise Pascal.
- ⇨ Axe « Education à la santé en milieu scolaire »

**1996-2009 : Membre associée au CURAPP (Centre Universitaire de Recherches sur l'Action Publique et le Politique. Epistémologie et Sciences Sociales), UMR 6054 du CNRS et du laboratoire de recherche SA-SO (Savoirs et Socialisation).**<br>⇨ Recherches dans l'axe 2 (Savoirs), sous-axe 3 (socialisation, trajectoires, identités).<br>⇨ Équipe associée au CNRS (UMR 6054) depuis 1982,.<br><br>**Axes de recherche :**<br>⇨ **Formation et professionnalisation des adultes, didactique, apprentissages en contexte :** professionnalisation des acteurs, analyse des pratiques professionnelles, didactique, influences d'un contexte plurilingue, prise en compte des spécificités socioculturelles, psychologiques et langagières dans les apprentissages.<br>⇨ **Accès différenciés des apprenants aux outils pratiques du travail intellectuel :** difficultés d'accès aux savoirs, difficultés d'apprentissage, problématique de l'illettrisme et connaissance des publics en situation précaire.<br>⇨ **Rapports différenciés aux savoirs en santé (notamment dans le cadre de la chronicité) et variations sociales de ces rapports (adultes en formation et hors structures) :** différences de résultats obtenus auprès de publics ayant bénéficié d'actions d'éducation, prise en compte des contextes, des dispositions individuelles et des configurations sociales. Etude de leurs variations.

Principaux travaux scientifiques

2006-2009	**Responsable scientifique de la recherche** « Évaluer les incidences de la survenue d'une maladie virale émergente chez des malades chroniques de milieux sociaux différenciés afin d'améliorer la prévention des risques en santé en milieu tropical »<br>⇨ Etude des variations sociales de l'impact d'une maladie émergente (le chikungunya) chez des patients atteints d'une maladie chronique (le diabète de type 2) ayant bénéficié de sessions d'éducation (nids d'apprentissage)<br>⇨ Projet financé par le Ministère de l'Outre-mer
2006-2009	**Direction scientifique de la collection** « nids d'apprentissage », Editions des Archives Contemporaines, Paris
2005-2008	**Responsable scientifique de la recherche** « Rapports à l'écriture et aux formes de savoirs vu à travers l'usage des photocopies à l'école », publication à l'INRP.
2003-2006	**Responsable scientifique de la partie ethnosociologique** de la recherche REDIA-PREV2, sous l'égide de l'INSERM : « Évaluation d'un programme d'incitation à la reprise de l'activité physique et d'éducation nutritionnelle sur le contrôle du diabète et les facteurs de risques cardio-vasculaires chez des patients diabétiques hospitalisés à la Réunion ».
2003-2006	**Responsable scientifique de la recherche** : « Les professionnels de santé et l'éducation des patients : analyse de parcours de formation, constantes et différences.»
1998-2001	**Responsable scientifique de la recherche** « Pratiques scripturales en formation professionnelle : construction des modes de socialisation et des stratifications sociales. »<br>=> Analyse et comparaison de 4 structures de formation professionnelle dans le domaine de la santé (ambulanciers, aides-soignants, infirmiers, sages-femmes).
1998-2000	**Responsable scientifique de la recherche :** « Quel apport des emplois-jeunes aux dispositifs de lutte contre l'illettrisme ? CARIF

Publications et communications

Publications d'ouvrages scientifiques

BALCOU-DEBUSSCHE M., (2006), *L'éducation des malades chroniques. Une approche ethnosociologique*. Ouvrage réalisé à partir d'une recherche menée sous l'égide de l'INSERM, Paris, Éditions des Archives Contemporaines, 280 p.
BALCOU-DEBUSSCHE M., (2004), *Écriture et formation professionnelle. L'exemple des formations de la santé*, Lille, Presses Universitaires du Septentrion, Collection Éducation et didactiques. Acquisition et transmission des savoirs. 264 p.

Chapitres d'ouvrages scientifiques

BALCOU-DEBUSSCHE M. (2009). « Une approche ethnosociologique de l'éducation thérapeutique : les nids d'apprentissage dans le diabète de type 2 ». *L'éducation thérapeutique en France : pratiques, modèles, évaluations*. Paris : INPES (à paraître).
BALCOU-DEBUSSCHE M., (2009), « L'alimentation, les soins, les maladies », in *La Réunion : une société en mutations*, Wolff E. & Watin M.. (dir), Univers créoles 7, Paris, Anthropos-Economica (à paraître).

BALCOU-DEBUSSCHE M., avec la collaboration de LEDEGEN G., (2003), "Scripteurs précaires en formation professionnelle : nature des difficultés, analyse de situations et orientations didactiques", *École et Éducation*, Tupin F. (dir), Univers créoles 3, Paris, Anthropos-Economica, pp. 201-230.

Co-direction d'ouvrages et/ou de revues

BURY J., FOUCAUD J., EYMARD Ch., BALCOU-DEBUSSCHE M. (2009). *L'éducation thérapeutique en France : pratiques, modèles, évaluations*. Paris : INPES (à paraître).
BALCOU-DEBUSSCHE M., MARSOLLIER Ch. (2009). Education à la santé. *Revue Expressions*, N° 32, http://www.reunion.iufm.fr/Recherche/Expressions/32.html
FOUCAUD J., BALCOU-DEBUSSCHE M., (2008), « Former à l'éducation du patient : quelles compétences ? Réflexions autour du séminaire de Lille, 11-13 octobre 2006, Paris, Editions INPES, 109 p.

Articles parus dans des revues et collections avec comité de lecture

BALCOU-DEBUSSCHE M., DEBUSSCHE X. (2009). Hospitalisation for type 2 diabetes: the effects of the suspension of reality on patients' subsequent management of their condition. *Qualitative Health Research*, 19, 1100-1115.

BALCOU-DEBUSSCHE M., (2009). « Un passeport santé pour le premier degré », *Revue Expressions*, N° 32, http://www.reunion.iufm.fr/Recherche/Expressions/32.html
BALCOU-DEBUSSCHE M., FOUCAUD J., (2008), « Quelles compétences en éducation du patient ? Analyse de contenu du séminaire de Lille, 11-13 octobre 2006, Paris, Editions INPES, 6 p.
BALCOU-DEBUSSCHE M., DEBUSSCHE X. (2008). « Type 2 diabetes patient education on Reunion Island : perceptions and needs of professionals at the initiation of a primary care management network ». *Diabetes & Metabolism*, vol 34, n°2.

BALCOU-DEBUSSCHE M., (2008), « Rapports des enseignants aux savoirs et à l'écriture vus à travers l'usage des photocopies à l'école », *Revue Française de Pédagogie*, INRP, Paris.

BALCOU-DEBUSSCHE M., (2008), « Apprendre à gérer l'apport de graisses dans l'alimentation » *Revue Expressions*, IUFM de la Réunion.

BALCOU-DEBUSSCHE M., (2007), « Accéder à la production littéraire en écrivant des maux… L'expérience d'une classe à PAC sur le thème de la violence. » *Revue Expressions*, IUFM de la Réunion.

BALCOU-DEBUSSCHE M., (2005), « L'écriture et la socialisation professionnelle des étudiants : l'exemple des infirmiers, sages-femmes et aides-soignants en formation initiale », *Le Sociographe*. *L'écriture, vecteur de lien social ?*, N° 18, Montpellier, IRTS-LR., pp 47-58.

DEBUSSCHE X., BALCOU-DEBUSSCHE M., (2005), « Une action de formation en éducation du patient centrée sur des situations d'apprentissage en groupe : l'expérience du Diplôme Universitaire de la Réunion », *Éducation du patient et enjeux de santé*, Vol 23, N°2, pp. 63-68.

BALCOU-DEBUSSCHE M., (2003), "Inégalités d'accès à une réflexion sur l'action par les pratiques scripturales : l'exemple de 4 formations professionnelles dans le domaine de la santé.", *Recherche et Formation*, *N° 44*, *La professionnalité en milieu difficile*, Paris, INRP, pp. 149-166.

BALCOU M., (1998), "Les professeurs des écoles en formation initiale et l'écriture", *Repères*, *Le français dans la formation des professeurs d'école*, N° 16, Paris, I.N.R.P., pp. 221-239.

Publications dans des actes de colloques et de congrès (nationaux, internationaux)

BALCOU-DEBUSSCHE M., (2009), « Implementation of a structured module for insulin therapy self management diabetes for type 2 in outpatient hospital settings in France», IDF, Montréal, octobre 2009.

BALCOU-DEBUSSCHE M., (2009), « Le passeport santé pour les élèves du premier degré », Communication orale aux journées de l'INPES, Paris, avril 2009.

BALCOU-DEBUSSCHE M., (2004), « Pratiques scripturales en formation initiale et construction des positionnements socioprofessionnels : l'exemple des formations dans le domaine de la santé. », 5ème congrès de l'AECSE, Paris, http://www.aecse.net/

BALCOU-DEBUSSCHE M., (2004), « Rapport aux savoirs des patients : vers des différenciations en éducation thérapeutique », 5ème congrès de AECSE (Association des Enseignants Chercheurs en Sciences de l'Education), Paris, http://www.aecse.net/

BALCOU-DEBUSSCHE M., DEBUSSCHE X., (2003), « Changes of perceptions and attitudes of Health Professionals after a 1-yr long course aiming at the implementation of diabetic patient education networks. », 18th International Diabetes Federation Congress, Paris 24-29 August 2003. Diabetologia, 46 (suppl 2), A95.

BALCOU-DEBUSSCHE M. (2003), « Problematicas sociales actuales y literatura de juventud : la collección Tropicante », Colloque international Lectura 2003, Ibby international, Cuba / La Havane, Octobre 2003, CD-Rom.

BALCOU-DEBUSSCHE M., (2003), « Pratiques scripturales et publics de faible niveau de qualification en formation professionnelle : usage des polycopiés et accès différenciés à la construction de savoirs (chez les ambulanciers en formation) », Lille, *Actes du colloque AECSE*., CD-Rom.

BALCOU-DEBUSSCHE M., DEBUSSCHE X., (2002), "From transmission to construction of knowledge and skills : a new paradigm for diabetes education and training", 5th IDF-WPR congress, Beijing, Diabetes Res Clin Pract, 56 (suppl 1), S34-S35.

BALCOU-DEBUSSCHE M. (2001), « Study of writing processes in situ : a useful tool for the analysis of training institutions.». Colloque Européen EERA (European Educational Research Association), Lille.

DEBUSSCHE X., BALCOU M., RODDIER M. (2000), « Didactic methods as a tool in the training of diabetes educators in Réunion Island. », Diabetes Res Clin Pract, 50 (suppl 1), S27.

DEBUSSCHE X., RODDIER M., BALCOU M., BOYER-DOYE M.C., (2000), « Anthropological approach of type 2 Diabetes Mellitus as a prerequisite in adaptative strategies of patient's education : the example of Réunion Island. », Diabetes Res Clin Pract, 50 (suppl 1), S27.

Publications universitaires (La Réunion, Amiens)

BALCOU-DEBUSSCHE M. (2001), *Pratiques scripturales en formation professionnelle : construction des modes de socialisation et des stratifications sociales. Analyse et comparaison de quatre lieux de formation professionnelle dans le domaine de la santé*. F. Ropé (dir), Thèse de Doctorat, UPJV Amiens, 502 p.
BALCOU M, (1997), *Les enseignants en formation et l'écriture*, F. Ropé (dir), Mémoire de D.E.A, UPJV Amiens.
BALCOU M., (1996), *Lire pour écrire, écrire pour lire*, F. Tupin (dir), Mémoire de maîtrise, Université de la Réunion.

Publication de rapports de recherche

BALCOU-DEBUSSCHE M., DEBUSSCHE X., LE MOULLEC N., YEUNG S., AH-KOON S., FAVIER F., (2005) « Rapports aux savoirs et à la maladie chez des patients diabétiques de type 2 ayant bénéficié d'un module d'éducation en institution hospitalière », Rapport de recherche INSERM.
BALCOU-DEBUSSCHE M. (2000*), Quel apport des emplois-jeunes aux dispositifs de lutte contre l'illettrisme ?*, Rapport d'enquête, St-Denis (Réunion), CARIF-OREF, 39 p.

BIOGRAPHICAL SKETCH

NAME : Jonathan B. Brown | POSITION TITLE : Senior Investigator

EDUCATION/TRAINING *(Begin with baccalaureate or other initial professional education, such as nursing, and include postdoctoral training.)*

INSTITUTION AND LOCATION	DEGREE *(if applicable)*	YEAR(s)	FIELD OF STUDY
Portland State University, Portland, OR	BS	1973	Social Sciences
Kennedy School of Government, Cambridge, MA	MPP	1976	Public Policy
Harvard University, Cambridge, MA	PhD	1981	Public Policy

A. POSITIONS AND HONORS.

1976–1980 Project Director, Executive Programs in Health Policy and Management, Harvard School of Public Health, Cambridge, MA.
1981 Instructor in Health Planning, Harvard School of Public Health, Cambridge, MA.
1981–1987 Assistant Professor of Public Policy and Health Planning, Department of Health Policy and Management, Harvard School of Public Health; Member, Institute for Health Research, Cambridge, MA.
1987–1990 Director of Organization Studies, Center for Health Research, Kaiser Permanente Northwest, Portland, OR.
1987–1998 Investigator, Center for Health Research, Kaiser Permanente Northwest, Portland, OR.
1999–present Senior Investigator, Center for Health Research, Kaiser Permanente Northwest, Portland, OR.

B. SELECTED PEER-REVIEWED PUBLICATIONS (IN CHRONOLOGICAL ORDER).

Books

Brown JB. *Health Capital Financing: Structuring Politics and Markets to Produce Community Health*. Ann Arbor, MI: Health Administration Press Perspectives, 1988.

Brown JB, et al. *Literature review of methods to support long-term strategic planning in health*. Copenhagen: World Health Organization- Europe, 1986.

Brown JB. *Unnecessary Coronary Artery Bypass Graft Surgery in Massachusetts.* Doctoral dissertation, Committee on Higher Degrees in Public Policy, Graduate School of Arts and Sciences, Harvard University, 1981.

Articles

Nichols GA, Brown, JB. Validating the Framingham Offspring Study Equations for predicting incident diabetes. *Am J Manag Care*. (in press)

Nichols GA, Hillier TA, Brown JB. Normal fasting plasma glucose and risk of Type 2 Diabetes diagnosis. *Am J Med* 2008; 121(6):519-24.

The Mount Hood 4 Modeling Group. Computer modeling of diabetes and its complications: A report on the Fourth Mount Hood Challenge Meeting. *Diabetes Care* 2007; 30(6):1638-46.

Nichols GA, Alexander CM, Girman CJ, Kamal-Bahl S, Brown JB. A contemporary analysis of secondary failure of successful sulphonylurea therapy. *Endocr Prac* 2007; 13(1):37-44.

Nichols GA, Hillier TE, Brown JB. Progression from newly acquired impaired fasting glucose to Type 2 Diabetes. *Diabetes Care* 2007; 30(2):228-33.

Brown JB, Vistisen D, Sicree R, Shaw J, Nichols GA, Zhang P. The economic impacts of diabetes. *International Diabetes Federation Diabetes Atlas, 3rd Edition*, 2006:237-65.

Nichols GA, Alexander CM, Girman CJ, Kamal-Bahl S, Brown JB. Treatment escalation and loss of glycemic control rise in HbA1c following successful initial metformin therapy. *Diabetes Care* 2006; 29(3):504-9.

Nichols GA, Brown JB. Higher medical care costs accompany impaired fasting glucose. *Diabetes Care* 2005; 28(9):2223-9.

Nichols GA, Koro CE, Gullion CM, Ephross SA, Brown JB. The incidence of congestive heart failure associated with anti-diabetic therapies. *Diabetes Metab Res Rev* 2005; 21(1):51-7. [Epub 2004 April 26]

Elmer PJ, Brown JB, Nichols GA, Oster G. Effects of weight gain on medical care costs. *Int J Obes Relat Metab Disord* 2004; 28(11):1365-73.

American Diabetes Association Consensus Panel. Guidelines for computer modeling of diabetes and its complications. *Diabetes Care* 2004; 27(9):2262-65.

Nichols GA, Gullion CM, Koro CE, Ephross SA, Brown JB. The incidence of congestive heart failure in Type 2 Diabetes: An update. *Diabetes Care* 2004; 27(8):1879-84.

Nichols GA, Brown JB. Functional status before and after diagnosis of Type 2 Diabetes. *Diabetic Med* 2004; 21(7):793-7.

Brown J, Nichols G, Perry A. The burden of treatment failure in Type 2 Diabetes. *Diabetes Care* 2004; 27(7):1535-40.

Bachman K, Brown JB. A large health care organization confronts obesity: A multifaceted population-based approach. *Group Pract J* 2004; 53(5):29-39.

Smith DH, Gullion CM, Nichols GA, Keith DS, Brown JB. Cost of medical care for chronic kidney disease and comorbidity among enrollees in a large HMO population. *J Am Soc Nephrol* 2004; 15(5):1300-06.

Keith DS, Nichols GA, Gullion CM, Brown JB, Smith DH. Longitudinal follow-up and outcomes among a population with chronic kidney disease in a large managed care organization. *Arch Intern Med* 2004; 164(6):659-63.

Brown J. Computer-simulated modeling in the management of diabetes*. Diabetes Voice* 2003; 48(4):33-3.

Brown J, Pedula K, Summers K. Diabetic retinopathy: Contemporary prevalence in a well-controlled population. *Diabetes Care* 2003; 26(9):2637-42.

Brown J, Nichols G. Slow response to loss of glycemic control in Type 2 Diabetes. *Am J Manag Care* 2003; 9(3):213-7.

Nichols GA, Brown JB. Unadjusted and adjusted prevalence of diagnosed depression in Type 2 Diabetes. *Diabetes Care* 2003; 26(3):744-9.

Shye D, Brown JB, Mullooly JP, Nichols GA. Understanding changes in primary care clinicians' satisfaction from depression care activities during adoption of selective serotonin reuptake inhibitors. *Am J Manag Care* 2002; 8(11):963-74.

Brown J, Rosenstein D, Mullooly J, O'Keeffe-Rosetti M, Robinson S. The impact of intensified dental care on outcomes in Acquired Immune Deficiency Syndrome. *AIDS Patient Care and STDS* 2002; 16(10):477-84.

Barrett PH, Beck A, Schmid K, Fireman B, Brown JB. Treatment decisions about lumbar herniated disc in a shared decision-making program. *Joint Comm J Qual Im* 2002; 28(5):211-9.

Nichols GA, Brown JB. The impact of cardiovascular disease on medical care costs in subjects with and without Type 2 Diabetes. *Diabetes Care* 2002; 25(3):482-6.

Beck A, Brown J, Boles M, Barrett P. Completion of advance directives by older health maintenance organization members: The role of attitudes and beliefs regarding life-sustaining treatment. *Am Geriatrics Society* 2002; 50(2):300-6.

Brown JB, Delea TE, Nichols GA, Edelsberg J, Elmer PJ, Oster G. Use of oral antithrombotic agents among HMO members with atherosclerotic cardiovascular disease. *Arch Int Med* 2002; 162(2):193-9.

Nichols GA, Hillier TA, Erbey J, Brown JB. Congestive heart failure in Type 2 Diabetes: Prevalence, incidence and risk factors. *Diabetes Care* 2001; 24(9):1614-9.

Brown JB, Nichols GA, Glauber HS, Bakst AW, Schaffer M, Kelleher CC. Health care costs associated with the escalation of drug treatment in patients with Type 2 Diabetes. *Am J Health Syst Pharm* 2001; 58(2):151-7.

Thompson D, Brown JB, Nichols G, Elmer PJ, Oster G. Body mass index and future health-care costs: A retrospective cohort study. *Obes Res* 2001; 9(3):210-18.

Nichols GA, Glauber HS, Javor K, Brown JB. Achieving further glycemic control in Type 2 Diabetes mellitus. *West J Med* 2000; 173(3):175-9.

Nichols GA, Brown JB. Following depression in primary care: Do family practice physicians ask about depression at different rates than internal medicine physicians? *Arch Family Med* 2000; 9(5):478-82.

Monsieur Daouda SISSOKO

Né le 14 Juin 1968 à Bamako (MALI)

Nationalité : Française

Adresse : 1, Route de Montgaillard
97400 Saint Denis, La Réunion, France
Tél : 05 47 74 84 48 (jusque fin Août 2009)
Contacts mail : daouda.sissoko@gmail.com

Domaines de compétences

Maladies Infectieuses et tropicales
Santé maternelle et infantile (Nutrition, santé reproductive, organisation et qualité des soins)
Surveillance épidémiologique, systèmes d'alerte précoce et investigations épidémiques
Recherche clinique et enquêtes en population

Formation médicale

2009 Inscrit sur la liste 1 d'aptitude aux fonctions de praticien hospitalier (Concours 2008)

2001 **Diplôme des études spécialisées complémentaires en Pathologie Infectieuse et Tropicale Clinique et Biologique,** 2e niveau de spécialisation (Université Lille 2- France)

1998 **Doctorat d'Etat en médecine et Diplôme des études spécialisées en Santé Publique et Médecine Sociale,** 1er niveau de spécialisation (Université Lille 2- France)

1998-99 Diplôme d'Université : Chimiothérapie anti-infectieuse (Université Lille 2- France)

1998-99 Diplôme Interuniversitaire : Infections nosocomiales (Université Lille 2- France)

1996-97 Diplôme d'Université : Infections à VIH (Université Lille 2- France)

1988-94 Premier et deuxième cycles des études médicales (Université Lille 2- France)

Formation scientifique

2008- Thèse de 3e cycle en Epidémiologie (Université de Bordeaux 2) / PhD Santé Publique (Université de Montréal) [Cotutelle]

Projet : Fardeau et modélisation médico économique des maladies à prévention vaccinale à Mayotte

Co-Directeurs : Pr Helen Trottier (Université de McGill), Pr Mira Johri (Université de Montréal), Pr Denis Malvy (Université de Bordeaux 2)

2002 **Diplôme des Etudes Approfondies en Epidémiologie et intervention en santé publique, option Epidémiologie Clinique** (Université Bordeaux 2- France)

1996-98 Maîtrise de Sciences Biologiques et Médicales : C1 Statistique et Modélisation/ C2 Méthodologie en Recherche Clinique et Epidémiologique (Université Lille 2- France)

Parcours professionnel

2003- 2009 Cellule interrégionale d'épidémiologie la Réunion Mayotte (Institut de veille Sanitaire et Direction régionale des affaires sanitaires et sociales de la Réunion): **Epidémiologiste médical senior**

2002-03 Service de Médecine interne, Centre Hospitalier Territorial de Mayotte : **Praticien hospitalier contractuel**

1998-02 Département des Maladies Infectieuses et du Voyageur du Centre Hospitalier Régional et Universitaire de Lille (CHRU)-CH de Tourcoing :

- Accueilli dans le cadre du Projet de recherche du DEA à l'Institut de Santé Publique, Epidémiologie et Développement (Institut national de la santé et de la recherche médicale ex U 330 - Bordeaux) : Méta-analyse des essais cliniques randomisés trithérapies versus bithérapies anti-rétrovirales (2001-2002)
- **Attaché Spécialiste (mi-temps**), 1999- 2001
- **Médecin de Recherche Clinique (mi-temps),** Agence Nationale de Recherches sur le Sida et les Hépatites (ANRS) affecté au CHRU de Lille, 1999-2001
- **Assistant spécialiste**, 1998-1999

1994-98 CHRU de Lille : **Interne des Hôpitaux**

Enseignement et encadrement

Enseignement pour les médicaux

2008- Faculté de Médecine de Bordeaux : Enseignant associé à la Chaire de Maladies infectieuses et tropicales

1999-2001 Faculté de Médecine de Lille :

Enseignements dirigés d'Infectiologie en 3 ème cycle de médecine générale
Enseignement de Pathologie Infectieuse et Tropicale : Diplôme d'Université Médecine et Logistique de l'Action Humanitaire
Faculté de Chirurgie Dentaire de Lille : Enseignement de pathologie infectieuse

Annexe 2: protocole d'accord avec les pairs éducateurs

Il est convenu entre l'ONG Santé Diabète Mali et le patient pair éducateur ………….. que dans le cadre de cette étude visant à renforcer l'éducation des personnes vivant avec le diabète dans la commune 1 du district de Bamako :

Engagement de l'ONG SDM :

- Assurer la formation et l'évaluation des patients pairs éducateurs.
- Fournir les outils nécessaires pour l'animation (livrets)
- Assurer les frais de déplacement des pairs éducateurs.
- Assurer le suivi global de l'activité

Engagement des patients pairs éducateurs :

- Mobiliser les participants aux éducations.
- Réaliser les animations prévues suivant les calendriers définis
- S'assurer que les animations se font par groupes de 10
- Signaler l'absence ou l'abandon d'un pair éducateur

En connaissance de cause, j'accepte d'être patient pair éducateur durant cette étude.

Fait à ………………………………….   Le ………../……/2011

Signature (ou empreinte)   Le responsable de l'étude

Annexe 3: mode opérationnel appareil Hba1c
