# Supplementary material for: Structured peer-led diabetes self-management and support in a low-income country: The ST2EP randomised controlled trial in Mali
Source: PLoS One. 2018 Jan 22;13(1):e0191262. doi: 10.1371/journal.pone.0191262 (PMC5777645; doi:10.1371/journal.pone.0191262)
Supplement: S4 File — (DOC) [file pone.0191262.s004.doc]

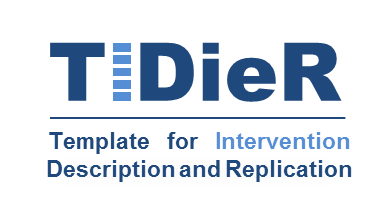
**The TIDieR (Template for Intervention Description and Replication) Checklist*:**

Information to include when describing an intervention and the location of the information

| **Item number** | **Item** | **Where located **** | |
| --- | --- | --- | --- |
|  |  | Primary paper  (page or appendix  number) | Other ^†^ (details) |
|  | **BRIEF NAME** |  |  |
| **1.** | ST2EP (Structured Type 2 diabetes self-management Education by Peers) | Page 4 | ______________ |
|  | **WHY** |  |  |
| 2. | |  |  | | --- | --- |   The ST2EP peer-led structured patient education intervention drew on the ‘Learning Nests’ (*Nids d’apprentissage*) approach: derived from socio-constructivist theory and takes into consideration the context of the illness, the prevailing health practices, and the chronic dimension of the disease. The complex nature of chronic diseases requires that therapeutic education takes into account both its biomedical and psychosocial dimensions. *Learning nests* work on knowledge to be built, and social, individual and contextual dimensions of learning, as well as the required decision of actions on a long-term basis, taking into account the heterogeneity of individuals and of their conditions. The approach promotes the understanding of key concepts and involves interactions with the social environment. Educational sessions are structured around 5 components: patients’ analysis of their own knowledge and practices, recognition of individual contexts and problem-solving, individual heterogeneity as an asset for self-assessment and action, culturally-tailored educational materials, and the long-term dimension of disease management. ‘Learning Nests’ incorporate and emphasise behavioural strategies as opposed to traditional didactic teaching. | P. 4-5_______ | Protocol (as supplementary file);  See also :  Balcou-Debussche M. *Rech Soins Infirm* 2012;110:45-59 (French)  Debussche X, et al.. *Cardiovasc Diab* 2012;91(11) , pages 4-5 |
|  | **WHAT** |  |  |
| **3.** | Materials: Describe any physical or informational materials used in the intervention, including those provided to participants or used in intervention delivery or in training of intervention providers. Provide information on where the materials can be accessed (e.g. online appendix, URL).  Learning nests use education materials that are developed with and for the target audience: local foods, contextual photographs, natural surroundings, occupations, clothing, appropriate reading level, coloured items. The material, process and the design of educational sessions are detailed in specific booklets for learners. Booklets for educators include a curriculum written protocol, available in French and, for some, in the English language (EPMC booklets; Education Prévention des Maladies Chroniques, éditions des archives contemporaines: www.archivescontemporaines.com/), allowing replication of the educational intervention. The content, approach and programme of each group session are detailed in specific booklets for learners and culturally adapted for Mali (food habits, language specificity, occupational and environment issues). Peer educators were also given specific booklets which included the session programme in French (*Education Prévention des Maladies Chroniques* or EPMC booklets^23^), allowing for the reproduction of the educational intervention. | Page 4 | Booklets accessible at www.litteratiesante.net |
| **4.** | Procedures: Describe each of the procedures, activities, and/or processes used in the intervention, including any enabling or support activities.  The ST2EP intervention included 4 quarterly courses delivered in the community by trained peer educators. Each course was composed of 4 different thematic sessions (2 hr-long; 4-10 participants) given over a period of 3 months (months 1-3, 4-6, 7-9, and 10-12). The themes addressed were cardiovascular risk management, food intake, exercise, and blood glucose and insulin management. | Page 4 | See also the protocol (supplementary file) |
|  | **WHO PROVIDED** |  |  |
| **5.** | For each category of intervention provider (e.g. psychologist, nursing assistant), describe their expertise, background and any specific training given.  Peer educators: Peer educators (PEs) were recruited from the local association of diabetic patients. This association provides counselling, education and support services to people with diabetes. It represents a key link between patients, educators and primary care services. The following criteria were used to select 10 PEs from the list of association members: having diabetes, living in the locality, undergoing regular checks by a referent-physician, volunteering to deliver educational sessions, and being fluent in both French and the local Bambara language. The recruited PEs received an initial 4-day training on how to facilitate and structure the different sessions (cardiovascular risk management, food intake, exercise, and blood glucose and insulin management). At the end of this training, a grid test was conducted to assess whether the PEs had understood the methodology and were qualified to facilitate the sessions. All 10 PEs were selected for another round of training, during which a 2^nd^ assessment grid was used to evaluate the facilitation skills of the PEs, under the supervision of the research team. A final evaluation was performed with a 3^rd^ assessment grid to select the 5 best PEs who were then actively involved in the project. | Page 5 | See also the protocol (supplementary file) |
|  | **HOW** |  |  |
| **6.** | Describe the modes of delivery (e.g. face-to-face or by some other mechanism, such as internet or telephone) of the intervention and whether it was provided individually or in a group.  Group sessions (4-10 participants), 2 hours long | Page 4 | _____________ |
|  | **WHERE** |  |  |
| **7.** | Describe the type(s) of location(s) where the intervention occurred, including any necessary infrastructure or relevant features.  Sessions delivered in the community by peer educators: in the facilities of the association of patients, commune 1 of Bamako district | Page 4 | _____________ |
|  | **WHEN and HOW MUCH** |  |  |
| **8.** | Describe the number of times the intervention was delivered and over what period of time including the number of sessions, their schedule, and their duration, intensity or dose.  4 quarterly courses delivered in the community by trained peer educators. Each course was composed of 4 different thematic sessions (2 hr-long; 4-10 participants) given over a period of 3 months (months 1-3, 4-6, 7-9, and 10-12). | Page 4 | _____________ |
|  | **TAILORING** |  |  |
| **9.** | If the intervention was planned to be personalised, titrated or adapted, then describe what, why, when, and how. | NA | _____________ |
|  | **MODIFICATIONS** |  |  |
| **10.^ǂ^** | If the intervention was modified during the course of the study, describe the changes (what, why, when, and how).  See below, point 11 | ____________ | _____________ |
|  | **HOW WELL** |  |  |
| **11.** | Planned: If intervention adherence or fidelity was assessed, describe how and by whom, and if any strategies were used to maintain or improve fidelity, describe them.  During the project 3 problems were encountered:  1 - The difficulty of mobilizing patients to move into the house of diabetes where the animations is taking place. Indeed, this represented an extra financial cost while patients often have difficulty paying for their travel to health facilities for their consultations  2- Many patients asked for more involvement of health care professionals to improve the link between education sessions and diabetes consultations  3 - The political situation in Mali who needed to shift a few weeks the continuation of activities.  Solution during the study :  1. We asked to the association of diabetic patients to support us to strengthen the involvement of patients to overcome the financial cost.  2. A communication was conducted among patients who follow the animations to explain:  - That the work is done in link with the doctor in the diabetes consultation  - That patients peer educators received strong training and validated their training  3. Delay for a short time the animation of education sessions as the political situation allows to resume normal protocol | _____________ | _____________ |
| **12.^ǂ^** | Actual: If intervention adherence or fidelity was assessed, describe the extent to which the intervention was delivered as planned.  Of the 76 participants in the intervention group, 4 were lost to follow-up before the 3^rd^ month, and 2 dropped out at 6 months after having attended 2 courses of 4 sessions each. Overall, the 5 PEs facilitated 177 educational sessions with a mean of 6.4 patients per group session in the intervention group. Among patients in the intervention group, 70 attended the 4 courses. | Page 7 | _____________ |

** **Authors** - use N/A if an item is not applicable for the intervention being described. **Reviewers** – use ‘?’ if information about the element is not reported/not sufficiently reported.

† If the information is not provided in the primary paper, give details of where this information is available. This may include locations such as a published protocol or other published papers (provide citation details) or a website (provide the URL).

ǂ If completing the TIDieR checklist for a protocol, these items are not relevant to the protocol and cannot be described until the study is complete.

* We strongly recommend using this checklist in conjunction with the TIDieR guide (see *BMJ* 2014;348:g1687) which contains an explanation and elaboration for each item.

* The focus of TIDieR is on reporting details of the intervention elements (and where relevant, comparison elements) of a study. Other elements and methodological features of studies are covered by other reporting statements and checklists and have not been duplicated as part of the TIDieR checklist. When a **randomised trial** is being reported, the TIDieR checklist should be used in conjunction with the CONSORT statement (see [www.consort-statement.org](http://www.consort-statement.org/)) as an extension of **Item 5 of the CONSORT 2010 Statement.** When a **clinical trial** **protocol** is being reported, the TIDieR checklist should be used in conjunction with the SPIRIT statement as an extension of **Item 11 of the SPIRIT 2013 Statement** (see [www.spirit-statement.org](http://www.spirit-statement.org/)). For alternate study designs, TIDieR can be used in conjunction with the appropriate checklist for that study design (see [www.equator-network.org](http://www.equator-network.org/)).
